# Applying a Network Approach To Characterize Gender Differences in Conduct Problems and Callous-Unemotional Traits among Children from Two Countries

**DOI:** 10.1007/s10802-025-01399-7

**Published:** 2026-01-16

**Authors:** María Álvarez-Voces, Natalie Goulter, Yael Paz, Beatriz Díaz-Vázquez, Laura López-Romero, Paula Villar, Amy L. Byrd, Samuel W. Hawes, Estrella Romero, Rebecca Waller

**Affiliations:** 1 Underisk Group, Department of Clinical Psychology and Psychobiology, Institute of Psychology (IPsiUS), Faculty of Psychology, University of Santiago de Compostela, Santiago de Compostela, Spain; 2 College of Education, Psychology, & Social Work, Flinders Institute for Mental Health & Wellbeing, Flinders University, Bedford Park, Adelaide, SA Australia; 3 Department of Psychology, University of Pennsylvania, Philadelphia, PA USA; 4 Department of Psychiatry, University of Pittsburgh School of Medicine, Pittsburgh, PA USA; 5 Department of Psychology, Florida International University, Miami, FL USA

**Keywords:** Callous-unemotional traits, Conduct problems, Cross-cultural, Gender differences, Network analysis

## Abstract

Callous-unemotional (CU) traits (i.e., low empathy, restricted guilt, limited prosociality) are associated with severe conduct problems (CP) across development. However, there is heterogeneity in how CP and CU traits manifest at different ages, between boys and girls, in different countries, and different measures. The current study investigated this heterogeneity by applying network analysis to two large mixed-gender samples from the United States (US) and Spain assessed at different ages, with parent ratings of CP, conduct disorder (CD) symptoms, and CU traits. Data were from the ABCD baseline study (US, *N* = 11,874, age *M* = 9.48, *SD* = 0.51, 47.8% girls) and social development sub-study (US, *N* = 2,426, age *M* = 11.52, SD = 0.73, 47.4% girls), as well as two waves of the ELISA study (Spain, *N* = 1,342, age *M* = 10.24, *SD* = 1.07, 50.2% girls; *N* = 1,259, age *M* = 10.92, *SD* = 1.01, 50% girls). There were similar rates of CP risk across countries and genders, with the exception that boys younger than age 12 had higher CP risk rates than girls in US. Boys also had higher CU traits than girls in both countries. Network analysis revealed stronger connectivity between items assessing CU traits than CP symptoms, which was consistent across measures and countries. Disobedience and deceitfulness were central symptoms across all samples. Relational aggression was more central among girls, and property destruction and theft were more central in the US. Results highlight the need for personalized interventions that target specific symptoms of CP and CU traits, which can help reduce the burden of antisocial conduct across the lifespan.

**Supplementary Information:**

The online version contains supplementary material available at 10.1007/s10802-025-01399-7.

Childhood conduct problems (CP) encompass intense anger, oppositionality, aggression, and rule-breaking. CP are one of the most prevalent mental health concerns during childhood, frequently prompting families to seek support from clinical services (Merikangas et al., 2022). If left unaddressed, CP can lead to conduct disorder (CD; repetitive, persistent behavior that violates the rights of others or age-appropriate social norms or rules) (American Psychiatric Association [APA], 2013). CD has a worldwide global prevalence of 2–2.5.5% (Fairchild et al., 2019) and is associated with lasting negative personal, social, and economic costs (Goulter et al., 2024).

An important issue facing prevention and intervention is the significant heterogeneity among children with CP. Thus, research has sought to identify more homogenous subgroups of children with CP with distinct symptom profiles, etiological factors, and treatment needs (Álvarez-Voces & Romero, 2024). For example, the Diagnostic and Statistical Manual of Mental Disorders (DSM-5; APA, 2013) has a CD specifier for the presence of callous-unemotional (CU) traits, which is defined by limited empathy, lack of guilt, and reduced sensitivity to the emotions of others (Frick et al., 2014; Waller et al., 2020). Around 25–30% of children with CP have CU traits, exhibiting distinct behavioral (Díaz-Vázquez et al., 2024), physiological (de Wied et al., 2012), neurobiological (Frick et al., 2014), and environmental profiles (Waller et al., 2018) relative to children with CP without CU traits and/or typically developing children (Frick et al., 2014). CU traits are associated with risk for severe and persistent violence, aggression, and rule-breaking across the lifespan (Cardinale & Marsh, 2020). Thus, research is needed to better characterize different symptom profiles of children with CP and high CU traits, which can inform more effective personalized treatments and intervention (Perlstein et al., 2023).

Importantly, there are well-established gender differences in childhood CP (Gutman et al., 2018), as well as in rates of violence, aggression, and psychopathy in adults (Sanz-García et al., 2021). For example, across childhood, oppositional defiant disorder (ODD) is nearly twice as common among boys than girls (1.59:1) (Demmer et al., 2017). In addition, boys are three times more likely to be diagnosed with CD than girls (Aryano et al., 2024) and are at greater risk for arrest, violence, and other serious forms of delinquency (Homer & Fisher, 2020; Liu & Miller, 2020). Boys are also more likely to receive a diagnosis of childhood-onset CD (i.e., before age 10) than girls, though CD rates are more comparable between boys and girls by adolescence (though see Moffit et al., 2001).

Examining gender differences in prevalence rates can help to characterize the broader impact of CD. However, to uncover distinct mechanisms that may lead to CD differentially in boys and girls, research must explore variations in symptom profiles. Some studies suggest that girls with CD engage in more relational aggression, while boys with CD exhibit relatively more physical aggression (Ackermann et al., 2019). Gender differences in CD symptom profiles are thought to arise from heightened susceptibility of boys to early aggression and disruptive behavior, reflecting greater propensity for language delays and impulsivity (Messer et al., 2006). Studies have also found gender differences in mechanisms linking temperament to psychopathology. For example, negative emotionality has been linked to CP in gender-specific ways, with fussiness more predictive in boys and fear more predictive in girls (Wang et al., 2016).

A similar picture emerges for CU traits, which are less severe among girls in population-based studies (Fanti et al., 2013), as well as in samples of children with CD (Colins et al., 2017). However, girls with CD and high CU traits engage in more bullying, delinquency, and both relational and proactive aggression and show fewer internalizing problems compared to girls with CD and low CU traits (Colins & Andershed, 2015). Moreover, among young children with high CU traits, risk for CP severity is comparable between boys and girls (Longman et al., 2016). It is also important to highlight the “*gender paradox*”, whereby girls with CD and high CU traits may experience greater impairment and higher psychiatric comorbidity than boys, even while girls are overall less likely to exhibit CD and CU traits (Konrad et al., 2022). From a mechanistic perspective, CU traits are associated with difficulties in both empathy and prosocial behavior (Waller et al., 2020). However, these difficulties may present differently across genders, with boys high on CU traits more likely to have difficulties in affective empathy (i.e., congruent emotional reactions with others) and girls more likely to have difficulties in cognitive empathy (i.e., understanding feelings of others) (Dadds et al., 2009).

One contributor to these differences may be societal expectations around gender, which shape emotional expression and interpersonal interactions in distinct ways for boys and girls. For example, boys are often socialized to view emotional vulnerability as a weakness and to value assertiveness and emotional control (Meimoun et al., 2024). These norms may limit engagement in affective empathy, particularly among boys with elevated CU traits, who may already be less sensitive to others’ emotions (Frick & White, 2008). In contrast, girls are typically encouraged to be emotionally expressive and attuned to others (Meimoun et al., 2024). Thus, when girls exhibit high CU traits, the discrepancy with these expectations could confer specific manifestations of impairment, such as greater cognitive empathy difficulty or social dysfunction (Dadds et al., 2009), which may relate to differences in the presentation of CP. Nevertheless, much remains to be understood about how CP and CU traits manifest in girls, especially their core symptoms, profiles, and comorbidities (Konrad et al., 2022; Moffitt, 2018).

One approach to characterize how CP symptoms and CU traits co-occur, including whether they manifest differently for boys and girls, is to leverage network analysis. Network analyses specify a series of nodes (i.e., symptoms) connected by edges (i.e., magnitude of association) (Borsboom & Cramer, 2013), allowing for the quantification of the global importance of symptoms (i.e., network centrality of nodes), while highlighting where symptoms are most important (i.e., individual edges, relative positioning in the network) (Borsboom & Cramer, 2013). By identifying symptom pairs and symptoms that bridge between clusters of nodes, network analysis provides an intuitive explanation of comorbidity (Fried et al., 2017). Network analysis has previously been used to characterize externalizing problems (Goulter et al., 2022), depression and anxiety (McElroy et al., 2018), and psychopathic traits (López-Romero et al., 2024). In one prior study of CP and CU traits (*N =* 608, ages 7–19), boys had lower centrality indices and lower within-sample stability, suggesting greater heterogeneity in CU traits and CD symptoms relative to girls (Goulter & Moretti, 2021). Items assessing callousness and delinquency were the most robust bridges between CU traits and CD symptoms for both genders.

However, no other studies have applied a network analytic approach to characterize specific symptom clusters of CP symptoms and CU traits in boys and girls, which can inform personalized therapeutic targets (Borsboom & Cramer, 2013). Unlike latent models that treat symptoms as indicators of an underlying latent factor (without necessarily having direct relations), network analysis identifies symptom connections and symptoms that are central or serve as bridges within a network. This approach offers greater precision at the symptom level, which can be used in therapeutic settings by targeting key symptoms to enhance treatment efficiency. In addition, by comparing networks across genders, a network approach can reveal structural differences that support individualized and developmentally sensitive interventions (McNally, 2016). Also, no studies have compared networks using different measures, which could identify construct versus measure differences in symptom profiles.

There may also be cross-cultural differences in how CP and CU traits manifest. Cultural factors impact the expression, interpretation, and perceived severity of psychiatric symptoms (Canino et al., 1997). The prevalence and presentation of CD varies across cultures, which is attributed to differences in parenting norms, gender expectations, and access to mental health resources (Wu et al., 2022). Likewise, the expression of CU traits varies between cultures. For example, a network analysis of CU traits among 8–15 years olds conducted in four countries (Australia, UK, US, and China) revealed that the item, *“*apologizes to people*”*, was more central in the UK network, attributed to the significance of apologizing within the British culture, which constitute a fundamental mode of expressing empathy and remorse. That is, an absence of apologies may serve as an indicator of empathy difficulties more strongly among British people (Deng et al., 2024). In a cross-cultural study of adult offenders, callousness/lack of empathy was a central item in the network for two samples from the US, while irresponsibility and parasitic lifestyle was central in a Dutch sample (Verschuere et al., 2018). Network analysis offers a useful method to elucidate the core characteristics of CP and CU traits. However, we lack knowledge about how CP and CU traits manifest differently in boys and girls in different cultures, including other European cultures. For example, in Spain, less emphasis is placed on individualism than in the US, with greater focus on group identity and collaboration. As a result, features of CU traits (e.g., low consideration for others) could be a more prominent network feature in Spanish samples, while similar items in the US could indicate assertiveness or self-confidence, reflecting a greater emphasis on personal success (Hofstede, 2011).

However, cultural influences extend beyond a single dimension of individualism versus collectivism. Additional frameworks offer a more nuanced perspective, including cultural tightness-looseness (Uz, 2015), relational mobility (Yuki & Schug, 2012), and cultural syndromes, such as honor or dignity (Leung & Cohen, 2011). Higher relational mobility and tighter institutional norms in the US could foster greater sensitivity toward early CP. In contrast, more stable social networks and flexible norms in Spain could mean certain behaviors are less readily pathologized or observable. These speculative hypotheses highlight a need for cross-cultural research to examine how cultural dynamics shape the identification, structure, and clinical relevance of CU and CP traits.

Thus, the goal of the current study was to advance understanding of how CU traits and CP are organized within and across genders and cultural contexts. Our network analytic approach aimed to identify which symptoms drive the presentation of others, providing more precise targets for intervention efforts. By applying this method to large, population-based samples from two countries, we aimed to advance cross-cultural research and inform prevention strategies tailored to developmental and sociocultural contexts. Our focus on ages 8–14 supported this goal, since serious acts of delinquency emerge during the period from middle childhood to adolescence (Moffitt, 2018). In our first aim, we compared the severity of CP symptoms and CU traits in boys and girls, as well as comparing differences in CP risk for children from Spain versus the US. We hypothesized lower prevalence of CP risk among girls in both samples. For our second aim, we used a network analytic approach to examine the structure of CP symptoms and CU traits, including testing whether the network structure varied between boys and girls or children from different countries. Based on prior work using traditional methods, we hypothesized physical aggression items would be most central for boys, while relational aggression items would be central for girls, with no hypothesized gender differences for CU traits. We had no directional hypotheses about differences in network structure for CP or CU traits based on country because of a lack of prior research.

## Methods

### Participants and Procedure

The study was pre-registered on the AsPredicted platform on 11/18/2024 (#199889). Participant details can be found in the Supplementary Material (Table S1).

#### Sample 1

Participants were from the baseline visit of the Adolescent Brain and Cognitive Development (ABCD) study, which recruited 11,874 children (47.8% girls; age, *M* = 9.48; *SD =* 0.51, *range* = 8–11; race/ethnicity, 52% White, 15% Black, 20.3% Hispanic, 2.1% Asian, and 10.5% others) to be followed to adulthood. Participants were recruited from 21 sites in the US from public, private, and charter schools. The institutional review boards at participating universities approved all procedures. All participants provided assent, and their legal guardians gave written consent for participation. For more details, see Garavan et al. (2018).

#### Sample 2

We also used data from the ABCD Social Development Substudy (ABCD-SD), conducted 3 years after baseline data collection. The ABCD-SD subsample is from five of the original ABCD sites and includes 2,426 participants that met all original ABCD inclusion criteria and completed additional measures of delinquency, victimization, personality, and social development (47.4% girls; age, *M* = 11.52; *SD =* 0.73, *range* = 9–14; ethnicity, 47.9% White, 28.6% Black, 10.7% Hispanic, 1.6% Asian, and 11.3% others) (see Hoffman et al. (2019) for details).

#### Sample 3

We used data from the longitudinal ELISA Project, which was conducted in Galicia, Spain. The ELISA project was approved by the former Spanish Ministry of Economy and Competitiveness and by the Bioethics Committee of the University of Santiago de Compostela (Reference: USC-21/2020) with seven data collection waves completed from 2017 to 2024. A total of 126 educational institutions (public, charter, and private schools) were contacted. 72 consented to participate in the project, and the families of students were invited to participate, with participation rates ranging from 25% to 50% across schools. The primary caregiver (biological mothers, 87.3%) completed questionnaires at each time point between March and June, with teachers distributing and collecting questionnaires. To ensure confidentiality, a pseudonymization process was implemented, and written informed consent was obtained from primary caregivers. No financial remuneration was provided to participants. In this study, we used data from waves 6 (2023) and 7 (2024), where our focal measures of interest were collected. At wave 6 (Sample 3a), data were available for 1,342 children aged 8–12 (50.2% girls; age, *M* = 10.24; *SD =* 1.07; race, White, 99.5%). At wave 7 (Sample 3b), there was data available for 1,259 children aged 9–13 (50% girls; age, *M* = 10.92; *SD* = 1.01; race, White, 99.6%).

### Measures

Supplementary Table S2 provides details on the measures, their translations, and supporting psychometric evidence.

#### Conduct Problems

In Samples 1, 2, and 3b, we assessed CP using the CP scale from the parent-reported Child Behavior Checklist (CBCL; Achenbach & Rescorla, 2001), rated on a 3-point scale (0 = *not true* to 2 = *very/often true*). To avoid content overlap with CU traits, a single item (lack of guilt) was omitted, resulting in a 16-item scale (Sample 1, ordinal α = 0.95; Sample 2, ordinal α = 0.96, Sample 3b, ordinal α = 0.82). We created screening-based risk groups for CP using the DSM-oriented scale and the normative sample norms from the CBCL manual (Achenbach & Rescorla, 2001) for US samples. For the Spanish sample assessed with the CBCL, we applied the Group 1 multicultural ASEBA norms (Achenbach & Rescorla, 2007). This decision was supported by a mean problem score in our sample that closely aligned with those reported for Group 1 societies, ensuring a culturally appropriate classification. To identify children at risk for CD, we used a threshold of *T* ≥ 65, which includes the borderline (*T* = 65–69) and clinical (*T* ≥ 70) ranges. This approach is consistent with ASEBA guidelines, allowing us to identify children with elevated problems, enhancing sensitivity to risk while maintaining adherence to standardized, norm-referenced procedures (Achenbach & Rescorla, 2007). Likewise, classifications were stratified by age group (6–11 and 12–18 years) and gender to ensure valid interpretability. In Sample 3a, parents completed the 10-item Conduct Problem Scale (Colins et al., 2014) based on DSM-IV criteria for ODD and CD, which includes eight items for CP (e.g., teases others; ordinal α = 0.92), with items rated on a 5-point scale (1 = *never* to 5 = *very often*). Clinical classification was precluded because there are no established clinical cut-offs for this measure.

#### CU TRAITS

In Samples 1, 2, and 3b, we used a 4-item measure used in previous ABCD studies (e.g., Hawes et al., 2020), which includes 3 reverse-scored items from Strengths and Difficulties Questionnaire (SDQ, Goodman, 1997; e.g., helpful) and one CBCL item (Achenbach & Rescorla, 2001; lack of guilt after misbehaving), rated on a 3-point scale (0 = *not true* to 2 = *certainly true*) (Sample 1, ordinal α = 0.86; Sample 2, ordinal α = 0.88, Sample 3b, ordinal α = 0.75). In Sample 2, we assessed CU traits using parent reports on 15 of the 24 original items from the Inventory of Callous-Unemotional Traits (ICU; Frick, 2004) that were available within ABCD-SD, with items rated on a 4-point scale (0 = *not true* to 3 = *definitely true*) (ordinal α = 0.96). To distinguish between the network analyses of Sample 2, we refer to Sample 2-ICU (used the ICU) and Sample 2-CU (used the 4-item CBCL-SDQ). Convergent validity of the 4-item CU traits measure was evaluated by computing its correlation with ICU total scores in Sample 2, yielding a moderate and significant association (*r* =.51, *p* <.001; *N* = 2,426), which falls within the expected range for adequate convergent validity (Hair et al., 2019). In Sample 3a, CU traits were assessed using the 10-item subscale (ordinal α = 0.95) of the Child Problematic Traits Inventory (Colins et al., 2014), with items rated on a 4-point scale (1 = *does not apply at all* to 4 = *applies very well*).

### Analytic Strategy

Items assessing CP and CU traits across measures and samples are listed in the Supplementary Material (Table S3), along with their corresponding abbreviations. All analyses were conducted with R version 4.4.1 using R Studio (R Core Team, 2024). Following guidelines, missing data were handled using multiple imputation, with 20 imputed datasets created and analyzing using a fully conditional specification with the default settings of the *mice* 3.0 package (van Buuren & Groothuis-Oudshoorn, 2011). More information about missing data is in the Supplementary Material (Tables S4-S8). We compared the prevalence of CP risk levels, as well as mean levels of CP and CU traits in boys and girls within and between samples using chi-square and *t*-tests. Second, using *ggraph* (Epskamp et al., 2012), we examined the network structure of CU traits and CP items, with each node representing an item and the edges representing the relationships between items (Epskamp et al., 2012). We estimated associations between items while controlling for all other items using Regularized Gaussian graphical models. These models estimate partial correlations, which represent the unique association between two items after accounting for all other items in the network. To reduce model complexity and avoid overfitting, we applied the Least Absolute Shrinkage and Selection Operator (LASSO). LASSO is a regularization technique that imposes a penalty on the sum of the absolute values of the estimated parameters, which encourages network sparsity. Small or spurious connections are shrunk to zero, resulting in a more parsimonious and interpretable model. Thus, only the strongest and most meaningful associations are retained enhancing the clarity and stability of the network structure (Epskamp et al., 2018). Using *qgraph* (Epskamp et al., 2012), we calculated strength centrality (i.e., sum of weighted number and strength of a node’s connections relative to other nodes) as a *z*-score (Costantini et al., 2015). We chose this metric as the primary indicator of node-level importance because it is more interpretable and stable than alternative centrality measures in psychological networks, particularly in the sparse structures produced by regularization methods (Bringmann et al., 2019). Third, using *bootnet* (Epskamp & Fried, 2015), we conducted bootstrapped difference tests to evaluate significant differences in strength centrality. Fourth, using *networktools* (Jones et al., 2017), we estimated bridge strength, which measures how strongly a node is connected to another syndrome (i.e., set of nodes forming a construct) (Jones et al., 2021). Fifth, we assessed network stability using the correlation stability (CS) coefficient, which indicates the maximum proportion of cases that can be removed while still maintaining ≥ 0.70 correlation between the original and reduced datasets with 95% confidence (acceptable values > 0.50, minimum acceptable threshold ≥ 0.25) (Epskamp et al., 2018). Finally, we re-ran analyses (i.e., network plots, centrality indices, precision analysis, and comparison of core and bridging nodes) separately for boys and girls in each sample. Following prior research (Verschuere et al., 2018), we focused on relative rather than absolute differences. In additional analysis, we conducted a Network Comparison Test (NCT) using networks based on Pearson correlations, as the application of NCT to networks derived from Spearman correlations is not yet validated (van Borkulo et al., 2023). All comparisons were restricted to networks constructed with the same measures. The results revealed no significant differences in global strength either between boys and girls within each sample or between countries for the same gender group (see Supplementary Table S39).

## Results

### Severity of CP Symptoms and CU Traits

Table 1 presents descriptive statistics and gender differences for all variables. Across samples, boys had higher CP scores than girls, though effect sizes were small (*range*,* d =* 0.20–0.31). In Sample 1 (ABCD baseline, US), boys aged ≤ 11 years showed a higher rate of CP risk than girls (7.4% vs. 5.5%; χ²(1) = 17.69, *p* <.001, Φ = 0.12), although the effect size was small. In Sample 2 (ABCD-SD baseline, US), no significant gender difference in CP risk was observed among children aged ≤ 11 (boys: 5.6%, girls: 6%; χ²(1) = 0.08, *p* =.78) or adolescents aged ≥ 12 (boys: 5.04%, girls: 5%; χ²(1) = 0.01, *p* =.95). Similarly, in Sample 3b (ELISA wave 7, Spain), no significant gender differences emerged in CP risk among children aged ≤ 11 (boys: 3.3%, girls: 4.4%; χ²(1) = 1.39, *p* =.24) or adolescents aged ≥ 12 (boys: 8.4%, girls: 5.3%; χ²(1) = 1.39, *p* =.24). Cross-national comparisons revealed significant differences in CP risk among boys aged ≤ 11, with higher rates in the US than in Spain (Sample 1 vs. 3b: χ²(1) = 10.51, *p* =.001, Φ = 0.04), and a marginal difference between Sample 2 and Sample 3b (χ²(1) = 3.11, *p* =.08). No significant cross-national differences were found for girls aged ≤ 11 (Sample 1 vs. 3b: χ²(1) = 1.01, *p* =.32; Sample 2 vs. 3b: χ²(1) = 1.37, *p* =.24), for boys aged ≥ 12 (Sample 2 vs. 3b: χ²(1) = 2.87, *p* =.09), or for girls aged ≥ 12 (Sample 2 vs. 3b: χ²(1) = 0.04, *p* =.85).

**Table 1:** Descriptive statistics and gender differences in study variables

Variable	Full sample	Boys	Girls	t-test_(df)_	d
	M (SD)	M (SD)	M (SD)		
**Sample 1: ABCD baseline**,** US**					
CP (CBCL)	1.28 (2.36)	1.56 (2.63)	0.97 (1.97)	13.89_(11413)_^a^	0.25
^1^CP (CBCL)	1.13 (2.12)	1.39 (2.38)	0.85 (1.75)	14.06_(11334)_^a^	0.26
CU (CBCL/SDQ)	0.90 (1.39)	1.09 (1.49)	0.70 (1.24)	15.57_(11735)_^a^	0.28
**Sample 2: ABCD-SD baseline**,** US**					
CP (CBCL)	1.99 (2.62)	2.38 (2.82)	1.57 (2.31)	7.75_(2390.9)_^a^	0.31
^1^CP (CBCL)	1.11(2.24)	1.33 (2.44)	0.88 (1.98)	5.01_(2386.1)_^a^	0.20
CU (CBCL/SDQ)	1 (1.46)	1.14 (1.56)	0.84 (1.34)	5.02 _(2406.8)_^a^	0.20
CU (ICU)	10.04 (6.35)	11.05 (6.57)	8.92 (5.90)	8.48_(2415.6)_^a^	0.34
**Sample 3a: ELISA wave 6**,** Spain**					
CP (CPs)	10.27 (3.06)	10.63 (3.35)	9.91 (2.70)	4.33_(1277.1)_^a^	0.24
CU (CPTI)	12.34 (3.80)	12.66 (3.93)	12.01 (3.64)	3.13_(1330.1)_^b^	0.17
**Sample 3b: ELISA wave 7**,** Spain**					
CP (CBCL)	1.17 (1.81)	1.37 (1.92)	0.98 (1.68)	3.86_(1237.7)_^a^	0.22
^1^CP (CBCL)	1.06 (1.64)	1.26 (1.76)	0.86 (1.48)	4.33 _(1225.5)_^a^	0.24
CU (CBCL/SDQ)	0.85 (1.14)	0.99 (1.21)	0.71 (1.04)	4.32_(1231.3)_^a^	0.24

*CP* Conduct Problems; *CBCL* Child Behavior Checklist 6/16; *ICU* Inventory of Callous Unemotional Traits; *CPTI* Child Problematic Traits Inventory; *SDQ* Strength and Difficulties Questionnaire. ^a^*p* < 0.001; ^b^*p* < 0.01; ^1^= Without “lack of guilt after misbehaving” item

For CU traits, boys consistently scored higher than girls across countries, though effects sizes were modest (*range*,* d* = 0.17–0.34). No differences were found between younger US and Spanish children, either for boys (Sample 1 vs. 3b: *t*(840.48) = 1.94, *p* =.05) or for girls (Sample 1 vs. 3b: *t*(837.36)=−0.32, *p* =.75). While older US children showed significant differences compared to their Spanish peers (boys: Sample 2-CU vs. 3b: *t*(1572.3) = 2.29, *p* =.02; *d* = 0.01; girls: Sample 2-CU vs. 3b: *t*(1568.2) = 2.24, *p* =.02; *d* = 0.01), the effect sizes were negligible.

### Network Analysis

Correlations and estimated network adjacency matrices are in the Supplementary Material (Tables S9–S38). For Samples 1 and 3b, we present network plots in the main manuscript, with plots for Samples 2-ICU, 2-CU, and 3a in the Supplementary Material (Figure S1-S4). All networks showed sufficient stability (i.e., CS ≥ 0.25) (Figure S5-S9).

#### Sample 1 (ABCD baseline, US, aged 8–11 years old)

**Fig. 1:**
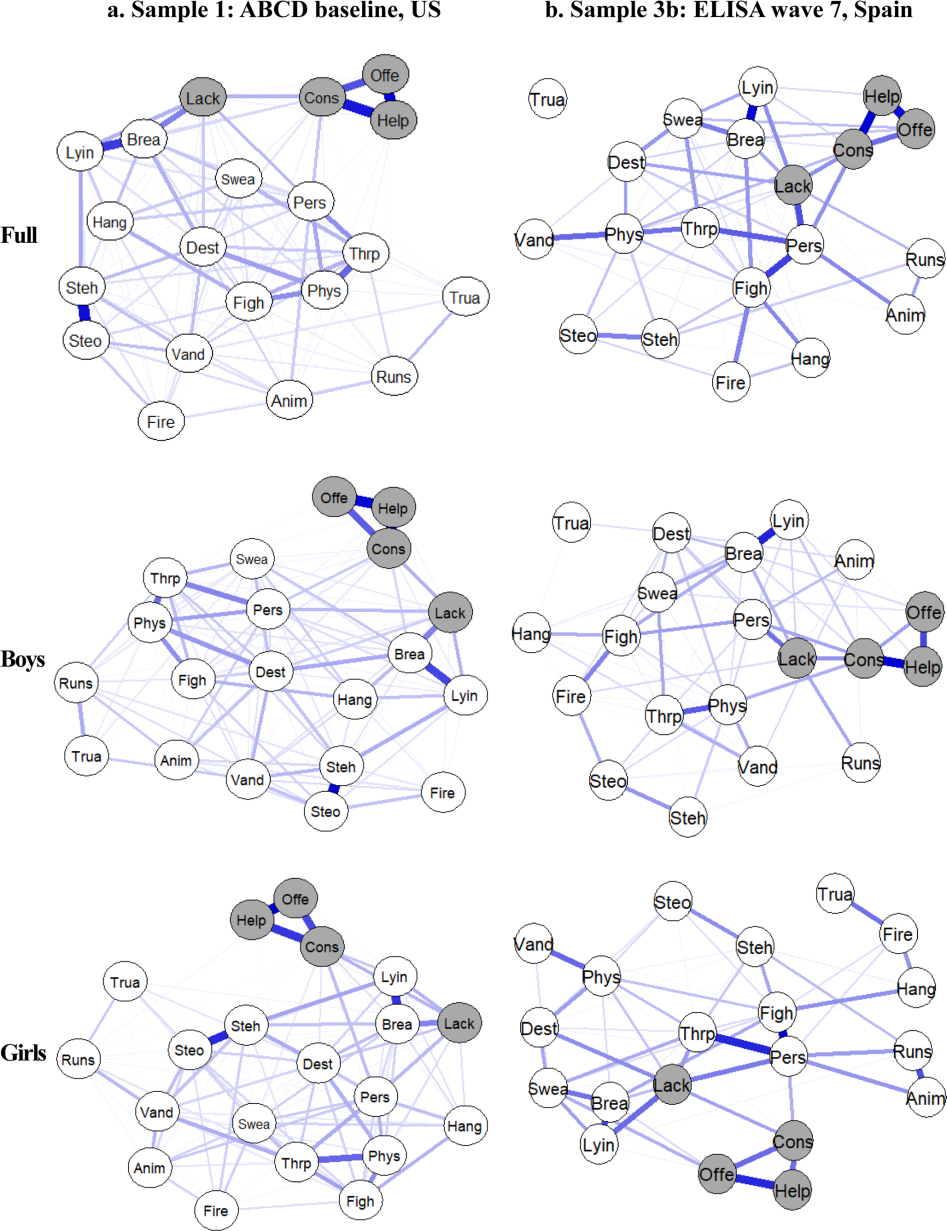
Network structure for (**a**) Sample 1: ABCD baseline, US and (**b**) Sample 3b: ELISA wave 7, Spain. CP items are in white and CU traits in gray

**Fig. 2:**
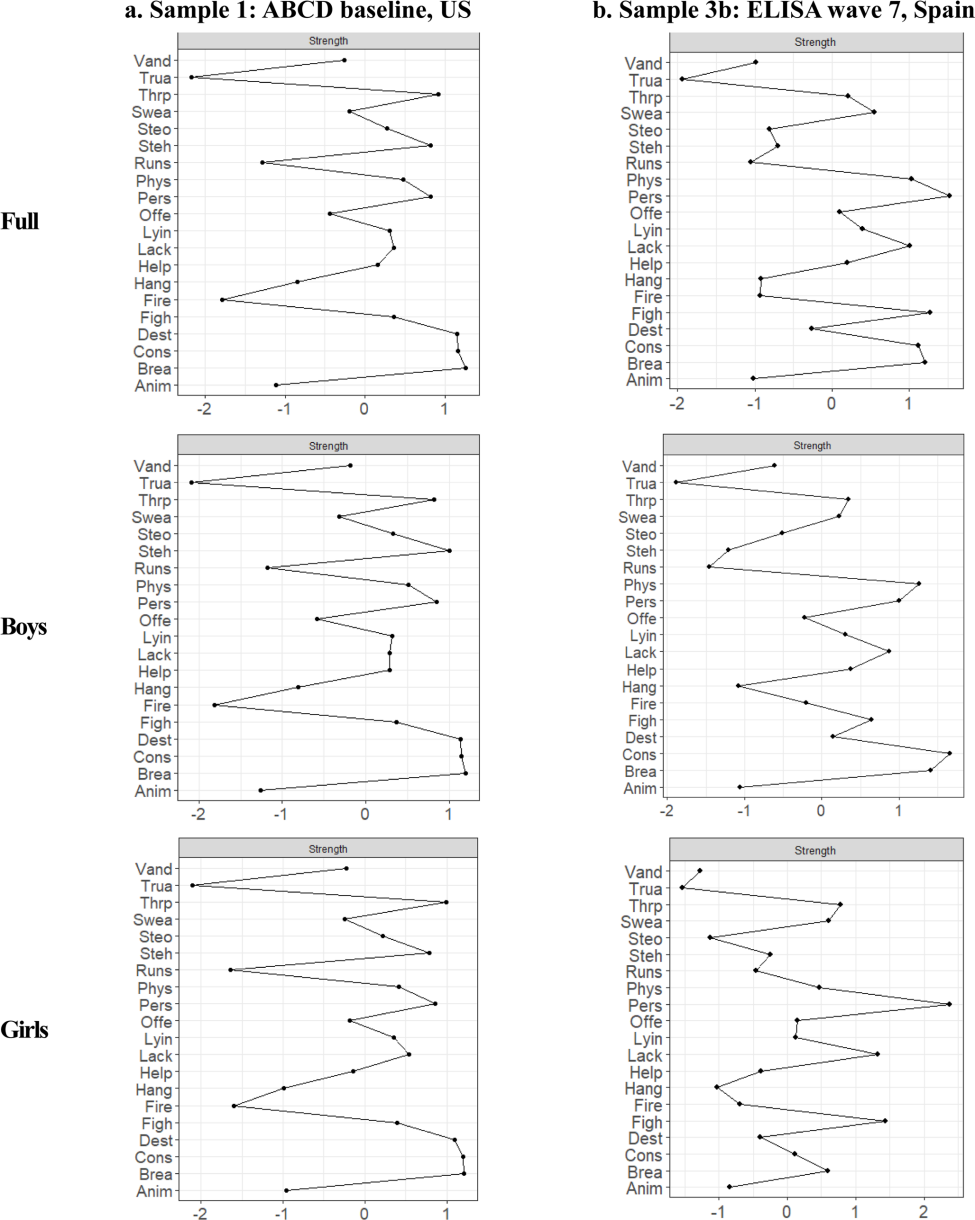
Centrality strength (*Z*-scores) for (**a**) Sample 1: ABCD baseline, US and (**b**) Sample 3b: ELISA wave 7, Spain, presented for the full sample and separately for boys and girls. Strength centrality is the sum of correlations between each node and all other nodes. Items are in descending alphabetical order

**Fig. 3:**
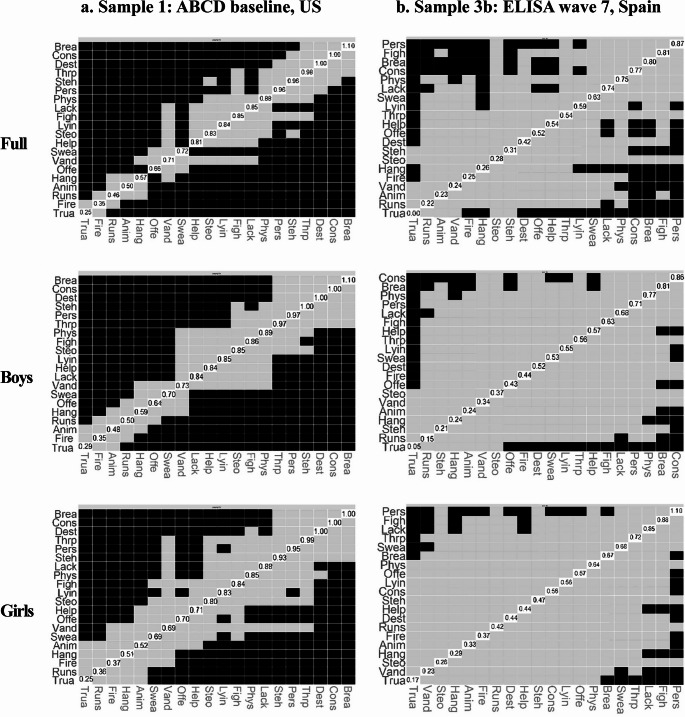
Bootstrapped differences of centrality strength for (**a**) Sample 1: ABCD baseline, US and (**b**) Sample 3b: ELISA wave 7, Spain, shown for the full sample, as well as separately for boys and girls. Black boxes denote significant differences (*p* <.05), gray boxes indicate nonsignificant differences, and white diagonal boxes display the corresponding values. The items are arranged in order of their scores

**Fig. 4:**
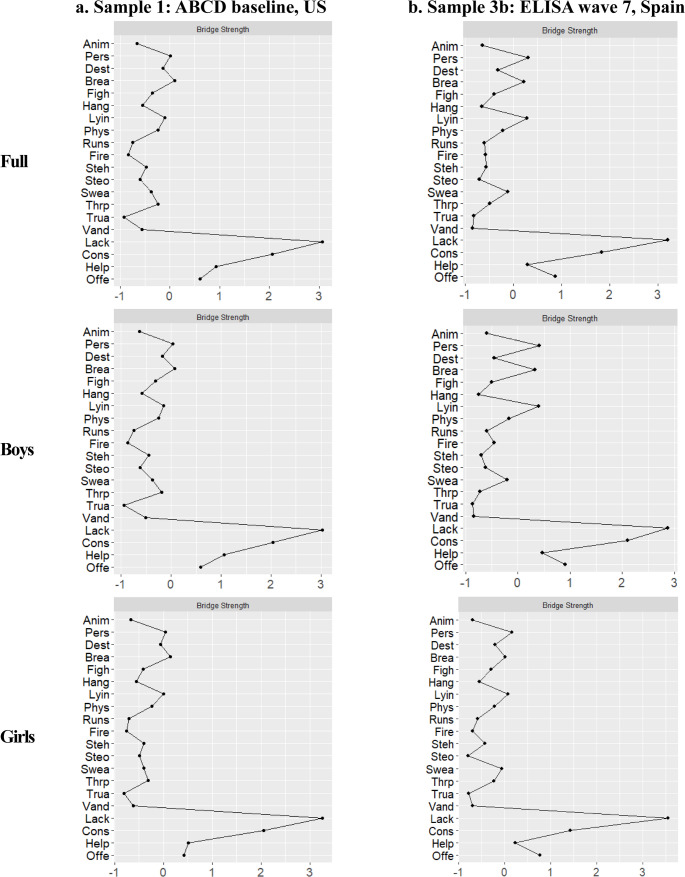
Bridge strength for (**a**) Sample 1: ABCD baseline, US and (**b**) Sample 3b: ELISA wave 7, Spain, for the full sample, as well as separately for boys and girls. The items are listed alphabetically, with those categorized as CP presented first, followed by those classified as CU traits

For network connectivity, the four CU traits items were strongly intercorrelated, as demonstrated by densely connected nodes and high edge weights (i.e., thickness and saturation) (Fig. 1). The item, “considerate of feelings”, had the highest centrality strength (*z* = 1.04) (Fig. 2), further supported by the bootstrapped differences in strength test (Fig. 3**)**. Findings were similar across boys (*z* = 1.04) and girls (*z* = 1.03). For the 16 CP items, the overall intercorrelation of nodes was less dense than for CU traits, with lower edge weights (see Supplemental Materials for adjacency matrices). However, items related to stealing, rule-breaking, and lying/cheating were strongly associated with each other, as reflected by densely connected nodes and relatively high edge weights within the network (Fig. 1). The item *“*breaks rules” demonstrated the highest centrality strength (*z* = 1.07) (Fig. 2), further supported by the bootstrapped differences test (Fig. 3). This test also identified significantly higher centrality of the items “destroys things” for boys (*z =* 1.04) and girls (*z* = 1.01), and “steals” for boys (*z* = 1.01). Finally, across all items, the CU traits item, “lack of guilt after misbehaving” exhibited the highest bridge strength (*z range* = 3.54–3.75) (Fig. 4).

#### Sample 2 (ABCD-SD baseline, US, aged 8–14 years old)

Using the fuller 15-item CU traits measure (Sample 2-ICU), CU traits items were more strongly intercorrelated than the CP items, with the closest connections between the items, “works hard”, “tries his/her best”, *“*apologizes”, and “feels bad or guilty” (Figure S1). The CU traits items, “works hard”, and “always tries his/her best” demonstrated the highest centrality strength in the full sample (*z* = 1.20 and 1.13), and for both boys (*z* = 1.18 and 1.20) and girls (*z* = 1.20 and 1.11) (Figure S2), further supported by the bootstrapped differences in centrality strength test (Figure S3). The CP items, “runs away from home”, “steals”, and “vandalism” showed the strongest associations. These patterns were consistent across boys and girls (Figure S1). The CP item with the highest centrality was “threatens people” (*z* = 1.11) (Figure S2), which was further supported by the bootstrapped difference test for centrality strength (Figure S3). The CP item “breaks rules”, demonstrated the greatest bridge strength (*z range* = 3.27–3.77) (Figure S4).

Using the brief CU traits measure (Sample 2-CU), the CU traits items were most densely connected with the greatest edge weights across all subsamples (Figure S1). The CU traits item with the highest centrality was “considerate of feelings” in all subsamples (*z range* = 1.06–1.08) (Figures S2 and S3). The CP items, “break rules”, “lying/cheating”, “steals from home” and “steals outside” were closely related for all subsamples (Figure S1). CP items with the highest centrality strength were “threatens people” (*z* = 1.08) and “destroys things” (*z* = 1.05) in the full sample, “threatens people” (*z* = 1.24) and “vandalism” (*z* = 1.08) for boys, and “destroys things” (*z* = 1.12) and “physically attacks people” (*z* = 1.10) in girls (Figures S2, S3). The CU item “lack of guilt” had the highest bridge strength for all samples (*z range* = 4.15–4.26) (Figure S4).

#### Sample 3a (ELISA wave 6, Spain, aged 8–12)

Correlations within CU traits and CP items were high across the full sample and for girls and boys. The CU traits items, “never expresses feelings of guilt” and “never shows a conscience” were more strongly related for boys than girls (Figure S1). Across all subsamples of Sample 3a, the CU traits items with the highest strength centrality were “seems indifferent” (*z range* = 1–1.11.11) and “lacks capacity to feel guilt/remorse” (*z range* = 1.03–1.25) (Figures S2 and S3). The CP item “hit without reason” showed the highest centrality strength in the full sample (*z* = 0.96) and for boys (*z* = 0.97), along with “has violated important rules” (*z* = 0.97). For girls the most central item was “has violated important rules” (*z* = 0.87) (Figures S2 and S3). Finally, the CP item “has violated important rules” showed the greatest bridge strength across all subsamples (*z range* = 2.69–2.97) (Figure S4).

#### Sample 3b ***(***ELISA wave 7, Spain, aged 9–13)

CU traits items were highly correlated across all subsamples of Sample 3b (Fig. 1). The item with the highest centrality strength was “considerate of feelings” in the full sample (*z* = 0.87) and for boys (*z* = 0.86), but for girls, it was “lack of guilt” (*z* = 0.85) (Figs. 2 and 3). The CP items, “breaks rules” and “lying/cheating” showed strong intercorrelations across all subsamples. The items “gets in fights”, “threatens people”, and “cruel to others” exhibited denser connections among girls than boys (Fig. 1). The CP items, “cruel to others” and “gets in many fights” demonstrated the highest centrality strength in the full sample (*z* = 0.87 and 0.81) and girls (*z* = 1.10 and 0.88). For boys, the CP items with the highest centrality were “breaks rules” (*z* = 0.81) and “physically attacks people” (*z* = 0.77; Fig. 2), further supported by the bootstrapped test (Fig. 3). Finally, the item with the highest bridge strength was the CU traits item, “lack of guilt after misbehaving” (*z range* = 2.31–2.97; Fig. 4).

#### Summary

We summarize the findings in Table 2. Across samples, CU traits items showed the densest connections, particularly “offers to help”, “considerate of feelings”, and “helpful”. Among CP items, the densest connections were observed for “lying/cheating”, and “breaks rules”. In addition, items with the highest centrality strength were “considerate of feelings” and “lack of guilt after misbehaving” (CU traits), and “breaks rules” and “lying/cheating” (CP). The item, “lack of guilt after misbehaving” (CU traits) demonstrated the highest bridge strength across samples and measures. There were some differences between boys and girls. For boys, the items, “physically attacks people”, “threatens people”, “runs away”, and “vandalism” showed the strongest associations with each other, with “steals”, and “vandalism” items showed greater centrality strength. For girls, the items “fights”, “cruel to others”, and “threatens people” items showed the strongest associations. Across countries, there were overlapping network features, with only stealing (“steals at home”, “steals outside”; US) and property destruction (“vandalism”, “destroys things”; US) items differing. Finally, CU traits items relating to uncaring, available only in Sample 2-ICU, exhibited greater centrality strength.

**Table 2:** Summary of key network analysis Findings, highlighting strongest Correlations, centrality strength, and Bridge strength

*Network metrics*		Sample 1 (ABCD baseline, US; ages 8–11)	Sample 2 (ABCD-SD baseline, US, ages 9–14)		Sample 3a (ELISA wave 6, Spain, ages 8–12)	Sample 3b (ELISA wave 7, Spain, ages 9–13)
			**Sample 2-ICU**	**Sample 2-CU**		
**Full sample**	**Correlations**	▪ offers to help ▪ considerate of feelings ▪ helpful	▪ feels bad/guilty ▪ apologizes ▪ always tries their best ▪ works hard	▪ offers to help ▪ considerate of feelings ▪ helpful	▪ lacks capacity to feel guilt ▪ does not express guilt	▪ offers to help ▪ considerate of feelings ▪ helpful
		▪ steals at home ▪ steals outside ▪ lying/cheating ▪ breaks rules	▪ runs away from home ▪ vandalism	▪ steals at home ▪ steals outside	▪ runs away from home ▪ vandalism	▪ lying/cheating ▪ breaks rules
	**Centrality**	▪ considerate of feelings	▪ works hard ▪ always tries their best	▪ considerate of feelings	▪ seems indifferent ▪ lacks capability to feel guilt	▪ considerate of feelings
		▪ breaks rules	▪ threatens people	▪ threatens people ▪ destroys things	▪ hit without reason	▪ cruel to others ▪ gets in many fights
	**Bridge**	▪ lack of guilt	▪ breaks rules	▪ lack of guilt	▪ violated important rules	▪ lack of guilt
**Boys**	**Correlations**	▪ offers to help ▪ considerate of feelings ▪ helpful	▪ feels bad/guilty ▪ apologizes ▪ always tries their best ▪ works hard	▪ offers to help ▪ considerate of feelings ▪ helpful	▪ seems indifferent ▪ does not care about what other people feel/think ▪ lacks capacity to feel guilt ▪ does not express guilt	▪ considerate of feelings ▪ helpful
		▪ steals at home ▪ steals outside ▪ lying/cheating ▪ breaks rules	▪ runs away from home ▪ vandalism	▪ runs away from home ▪ vandalism ▪ physically attacks people ▪ threatens people		▪ lying/cheating ▪ breaks rules
	**Centrality**	▪ considerate of feelings	▪ works hard ▪ always tries their best	▪ considerate of feelings	▪ seems indifferent ▪ lacks capacity to feel guilt	▪ considerate of feelings
		▪ breaks rules ▪ destroys things ▪ steals outside	▪ threatens people ▪ breaks rules	▪ threatens people ▪ vandalism	▪ has hit without reason ▪ has violated important rules	▪ breaks rules ▪ physically attacks people
	**Bridge**	▪ lack of guilt	▪ breaks rules	▪ lack of guilt	▪ violated important rules	▪ lack of guilt
**Girls**	**Correlations**	▪ offers to help ▪ considerate of feelings ▪ helpful	▪ feels bad/guilty ▪ apologizes ▪ always tries their best ▪ works hard	▪ offers to help, ▪ considerate of feelings ▪ helpful	▪ seems indifferent ▪ does not share others’ joy/sorrow ▪ lacks capacity to feel guilt ▪ does not express guilt	▪ offers to help ▪ considerate of feelings ▪ helpful
		▪ steals at home ▪ steals outside ▪ lying/cheating ▪ breaks rules	▪ gets in many fights ▪ threatens people	▪ gets in many fights ▪ threatens people		▪ gets in many fights ▪ cruel to others ▪ threatens people
	**Centrality**	▪ considerate of feelings	▪ works hard ▪ always tries their best	▪ considerate of feelings	▪ seems indifferent ▪ lacks capacity to feel guilt	▪ lack of guilt
		▪ breaks rules ▪ destroys things	▪ destroys things ▪ physically attacks people	▪ destroys things ▪ physically attacks people	▪ violated important rules	▪ cruel to others ▪ gets in many fights
	**Bridge**	▪ lack of guilt	▪ breaks rules	▪ lack of guilt	▪ violated important rules	▪ lack of guilt

## Discussion

We examined gender and country differences in rates of CP risk and levels of CU traits and CP in late-childhood and early adolescence, as well exploring differential patterns of symptom presentation based on gender and culture. First, consistent with a large prior literature (Aryano et al., 2024), boys in the US exhibited higher rates of CP risk than girls at ages 8–11. However, consistent with evidence that CD rates equalize by adolescence (Moffitt, 2018), there were no gender differences in 12–14 year olds from the US or Spain. CP risk rates were largely similar between the US and Spain, except for relatively higher rates among boys under 11 years old in the US. Despite the small effect size, these findings warrant replication and further investigation. Across samples, consistent with prior research (Fanti et al., 2013), boys had higher CU traits than girls, with little evidence for country differences. Future research is needed to establish clinical cut-offs normed across countries and to investigate different factors that impact assessment, presentation, or interventions for CD and CU traits (Perlstein et al., 2023).

Our findings suggest that socio-cultural mechanisms, such as income inequality, gendered expectations, and developmental transitions, might differentially shape the manifestation of CP across contexts. For example, the higher prevalence of risk for CP among young boys in the US may reflect greater income inequality, a known risk factor for CP (Mills-Koonce et al., 2016; OECD, 2024). By adolescence, this difference appeared to dissipate, possibly due to the growing influence of peer and school environments across countries, which could mitigate earlier community- or family-based disparities (Chen et al., 2015). However, the small effect sizes and use of a single screening instrument (i.e., the CBCL) caution against drawing strong conclusions.

Second, we leveraged network analysis to characterize how different items assessing CU traits and CP cohered together across samples. Consistent with one prior study of 7–19-year-olds (Goulter & Moretti, 2021), CU traits items were strongly inter-related, with densely connected nodes and elevated weight loadings across countries and genders. The intercorrelations of CP items were generally weaker. One possible explanation is that CU traits were developed as a narrower phenotype to reduce heterogeneity in the range of behaviors that produce a CD diagnosis (Frick et al., 2014). We also found stronger associations between relational aggression items in girls (cruel to others, threatens people) in both countries, suggesting that this form of aggression is particularly representative of CP among adolescent girls (Ackermann et al., 2019).

Both disobedience (e.g., breaks rules) and deceitfulness (e.g., lying/cheating) were central symptoms across genders and countries, suggesting that violating social norms and lying are perceived similarly in both contexts and serve as key indicators to assess CP. This finding is supported by prior research, which also identified lying/cheating as a central feature of CP (Goulter et al., 2022). Future research should explore country-specific norms, as perceptions of disobedience may differ across contexts. Conversely, the least central items across countries and genders were those related to severe rule violations (e.g., truancy), setting fires, and animal cruelty. These behaviors are likely more infrequent, more context-specific, or influenced by external factors, making them potentially less useful as CP indicators. Aligning with prior research, we found that relational aggression items were more central for girls in both countries. However, in contrast to prior studies (Ackermann, et al., 2019), physical aggression items showed less network centrality and were similar across genders.

One difference between countries was the higher centrality strength of property destruction/theft items in the US (e.g., vandalism, steals). This finding could reflect a host of differences between the US and Spain in the interplay of cultural, socioeconomic, and ecological factors (Ishoy, 2015; Mocan & Rees, 2005). Moreover, our results align with property crime rates that are 25 times higher in the US than Spain (6,600 vs. 253 per 100,000 residents in 2023; Federal Bureau of Investigation [FBI], 2024, Instituto Nacional de Estadística [INE], 2024). One explanation for these differences is cultural norms, with individual autonomy, assertiveness, and competition emphasized more in the US, potentially fostering greater tolerance of behaviors that transgress social or property boundaries. In contrast, the more collectivist orientation of Southern European cultures, including Spain, may discourage behavior that disrupts group harmony or public order (Hofstede, 2011). Additionally, greater economic inequality in the US may contribute to increased alienation and status anxiety among youth, potentially amplifying the frequency and visibility of property-related CP (Wilkinson & Pickett, 2009). Consistent with our other findings, the predominance of these items among boys could reflect the stronger peer influences and social reinforcement of these behaviors for boys than girls (Moffitt et al., 2001).

For the CU traits items, several uncaring and callousness reverse-scored items (e.g., “works hard”, “considerate of feelings”, “helpful”) were central across genders. Moreover, the emergence of the callousness item “lack of guilt after misbehaving” as the strongest bridge between CP and CU traits across genders, ages, measures, and countries, highlights the crucial role of an absence of guilt or remorse underpinning broader manifestations of CP and CU traits. While shared method bias is a concern, the fact that a conceptually similar item (i.e., “lacks the capacity to feel guilt/remorse”) emerged as a key bridging item in a sample that used a different measure of CU traits strengthens the robustness of this finding. An absence of guilt or remorse may drive the expression of more severe and persistent CP, as children do not experience the usual emotional discomfort or regret that typically inhibits antisocial conduct (Waller & Wagner, 2019). Interestingly, fewer studies have investigated links between CU traits and guilt. This is surprising given the many explicit references to guilt and remorse in measures of CU traits, including the ICU and DSM-5 diagnostic criteria (Waller et al., 2020). The developmental literature has also emphasized the importance of guilt for promoting and eliciting cooperation and prosocial behavior from ages 2–3 years old (Vaish, 2018). Thus, addressing difficulties with moral emotions that are associated with the development of CU traits remains a critical research need to prevent escalation to severe CP (Cardinale & Marsh, 2020).

Study strengths include the application of network analysis to two large independent cohorts from different countries assessed across late-childhood and early adolescence, with a range of measures. We generated an intuitive, graphical visualization of the interplay of CP and CU traits, which supports the detection of clustering patterns and community structures, and estimates unique associations between items (Borsboom & Cramer, 2013; Epskamp et al., 2018). However, findings should be considered alongside several limitations. First, we relied on parent report, which can inflate relationships due to shared-method variance and is susceptible to recall bias or subjective interpretations of items. While parents are considered dependable informants of child CP and CU traits, future research should incorporate multiple informants (Goulter & Moretti, 2021). Second, CP risk rates were estimated using CBCL cut-off scores rather than clinical assessment. Although these cut-offs are widely accepted for identifying children at risk of CD, they do not constitute a formal diagnosis. Consequently, our findings represent probable risk rather than definitive clinical cases and should be interpreted with this distinction in mind. Third, we did not adjust for age and racial/ethnic composition in analyses. Although the sample consisted mainly of children and early adolescents and the age ranges were relatively narrow, some age-related variation may have influenced the results. Moreover, most participants were White or European, which limits generalizability, particularly given that racialized experiences may shape the development and reporting of CP and CU traits. Fourth, the stability of some networks approached the unacceptable threshold (e.g., Sample 3b; Sample 2-ICU girls), indicating heterogeneity in CP symptoms and CU traits, requiring cautious interpretation of findings from these samples. Fifth, concerns about the replicability and stability of network analyses highlights the need for methodological improvements to improve robustness and generalizability (Forbes et al., 2017). Finally, we compared networks for boys versus girls using a gender- versus sex-based definition. However, findings were similar when we excluded children who identified as trans or gender non-conforming from analyses.

Overall, we found similar risk rates of CP across late-childhood and early adolescence in US and Spanish samples (with relatively higher rates observed in US boys ≤ 11). Boys had higher CP risk rates than girls among US children aged ≤ 11. Boys scored higher on CU traits than girls, with similar effects across countries and measures. Based on visual inspection of the network structures and the adjacency matrix values, items assessing CU traits appeared to show stronger associations with each other than CP, perhaps reflective of CU traits being a conceptually narrower construct. Across both countries, disobedience and deceitfulness were central CP items and uncaring and lack of guilt were central CU traits items. Relational aggression was more central for girls, while property destruction/theft was more central in the US. We highlight the utility of targeting specific CP symptoms rather than broader, latent externalizing factors (Borsboom & Cramer, 2013), with the potential that US-based interventions specifically target property destruction/theft, while relational aggression be universally prioritized for girls. Given the link between CU traits and severe CP, interventions that target the central features of CU traits are needed, especially given evidence that such traits are modifiable (Perlstein et al., 2023).

## Supplementary Information

Below is the link to the electronic supplementary material.

Supplementary Material 1 (PDF 2.45 MB)

## Data Availability

The data that support the findings of this study are available from MAV and RW upon reasonable request.
