# Supplementary material for: Applying a Network Approach To Characterize Gender Differences in Conduct Problems and Callous-Unemotional Traits among Children from Two Countries
Source: Res Child Adolesc Psychopathol. 2026 Jan 16;54(1):10. doi: 10.1007/s10802-025-01399-7 (PMC12811331; doi:10.1007/s10802-025-01399-7)
Supplement: Supplementary file 1 — Supplementary Material 1 (PDF 2.45 MB) [file 10802_2025_1399_MOESM1_ESM.pdf]

Supplementary Materials

Applying a Network Approach to Characterize Gender Differences in Conduct Problems and Callous-Unemotional Traits among Children from Two Countries

Sections

1. Demographics **4**
Table S1 Sociodemographic of the Samples 4
2. Measures information **5**
Table S2 Translation Approaches and Psychometric Support for Assessment Instruments 5
Table S3 List of Items Included in the Network Analysis 6
3. Analysis of missing data **8**
Table S4 Missing data in Sample 1: ABCD baseline, US 8
Table S5 Missing data in Sample 2-ICU: ABCD-SD baseline, US 9
Table S6 Missing data in Sample 2-CU: ABCD-SD baseline, US 10
Table S7 Missing data in Sample 3a: ELISA wave 6, Spain 11
Table S8 Missing data in Sample 3b: ELISA wave 7, Spain 12
4. Spearman correlations of the study variables **13**
Table S9 Spearman Correlations Sample 1 Full: ABCD baseline, US 13
Table S10 Spearman Correlations Sample 1 Boys: ABCD baseline, US 14
Table S11 Spearman Correlations Sample 1 Girls: ABCD baseline, US 15
Table S12 Spearman Correlations Sample 2-ICU Full: ABCD-SD baseline, US 16
Table S13 Spearman Correlations Sample 2-ICU Boys: ABCD-SD baseline, US 17
Table S14 Spearman Correlations Sample 2-ICU Girls: ABCD-SD baseline, US 18
Table S15 Spearman Correlations Sample 2-CU Full: ABCD-SD baseline, US 19
Table S16 Spearman Correlations Sample 2-CU Boys: ABCD-SD baseline, US 20
Table S17 Spearman Correlations Sample 2-CU Girls: ABCD-SD baseline, US 21
Table S18 Spearman Correlations Sample 3a Full: ELISA wave 6, Spain 22
Table S19 Spearman Correlations Sample 3a Boys: ELISA wave 6, Spain 23
Table S20 Spearman Correlations Sample 3a Girls: ELISA wave 6, Spain 24
Table S21 Spearman Correlations Sample 3b Full: ELISA wave 7, Spain 25
Table S22 Spearman Correlations Sample 3b Boys: ELISA wave 7, Spain 26
Table S23 Spearman Correlations Sample 3b Girls: ELISA wave 7, Spain 27
5. Adjacency Matrix of the Estimated Psychological Network (EBICglasso, Spearman) **28**
Table S24 Adjacency matrix of the estimated network for Sample 1 Full: ABCD baseline, US 28
Table S25 Adjacency matrix of the estimated network for Sample 1 Boys: ABCD baseline, US 29
Table S26 Adjacency matrix of the estimated network for Sample 1 Girls: ABCD baseline, US 30
Table S27 Adjacency matrix of the estimated network for Sample 2-ICU Full: ABCD-SD baseline, US 31
Table S28 Adjacency matrix of the estimated network for Sample 2-ICU Boys: ABCD-SD baseline, US 32
Table S29 Adjacency matrix of the estimated network for Sample 2-ICU Girls: ABCD-SD baseline, US 33
Table S30 Adjacency matrix of the estimated network for Sample 2-CU Full: ABCD-SD baseline, US 34
Table S31 Adjacency matrix of the estimated network for Sample 2-CU Boys: ABCD-SD baseline, US 35

Table S32 Adjacency matrix of the estimated network for Sample 2-CU Girls: ABCD-SD baseline, US 36
Table S33 Adjacency matrix of the estimated network for Sample 3a Full: ELISA wave 6, Spain 37
Table S34 Adjacency matrix of the estimated network for Sample 3a Boys: ELISA wave 6, Spain 38
Table S35 Adjacency matrix of the estimated network for Sample 3a Girls: ELISA wave 6, Spain 39
Table S36 Adjacency matrix of the estimated network for Sample 3b Full: ELISA wave 7, Spain 40
Table S37 Adjacency matrix of the estimated network for Sample 3b Boys: ELISA wave 7, Spain 41
Table S38 Adjacency matrix of the estimated network for Sample 3b Girls: ELISA wave 7, Spain 42
6. Network Comparisons Test (Pearson Correlations) 43
Table S39 Results of Network Comparisons Tests (Restricted to Networks Estimated with the Same Measures) 43
7. Network Analyses of Samples 2-ICU, 2-CU, and 3a 44
Figure S1 Network structure for Sample 2-ICU, Sample 2-CU, and Sample 3a 44
Figure S2 Centrality strength for Sample 2-ICU, Sample 2-CU, and Sample 3a 46
Figure S3 Bootstrapped differences of centrality strength for Sample 2-ICU, Sample 2-CU, and Sample 3a 48
Figure S4 Bridge strength for Sample 2-ICU, Sample 2-CU, and Sample 3a 50
8. Network Stability Strength across all Samples 52
Figure S5 Stability strength for Sample 1: ABCD baseline, US 52
Figure S6 Stability strength for Sample 2-ICU: ABCD-SD baseline, US 53
Figure S7 Stability strength for Sample 2-CU: ABCD-SD baseline, US 54
Figure S8 Stability strength for Sample 3a: ELISA wave 6, Spain 55
Figure. S9 Stability strength for Sample 3b: ELISA wave 7, Spain 56
References 57

1. Demographics

Table S1

Sociodemographic of the Samples

Variable	Sample 1: ABCD baseline, US				Sample 2 ABCD-SD baseline, US				Sample 3a: ELISA wave 6, Spain				Sample 3b: ELISA wave 7, Spain			
	n	%	*M* (*SD*)	Range	*n*	%	*M* (*SD*)	Range	*n*	%	*M* (*SD*)	Range	*n*	%	*M* (*SD*)	Range
**Age**	11,857		9.48 (0.51)	8-11	2,335		11.52 (0.73)	9-14	1,331		10.24 (1.07)	8-12	1,253		10.92 (1.01)	9-13
**Gender**[1]	11,857				2,426				1,342				1,259			
Boy	6181	52.1			1263	52.1			668	49.8			632	50.2		
Girl	5664	47.8			1150	47.4			673	50.2			626	49.8		
Transgender boy	2	0.02			3	0.1			0	0			0	0		
Transgender girl	4	0.03			2	0.1			1	0			1	0		
Queer	2	0.02			3	0.1			0	0			0	0		
Other	4	0.03			5	0.2			0	0			0	0		
**Ethnicity**	11,866				2,426				1,116				1,003			
White	6173	52			1161	47.9			1,110	99.5			999	99.6		
Black	1784	15.1			694	28.6										
Hispanic	2410	20.3			260	10.7										
Asian	252	2.1			38	1.6										
Other	1247	10.5			273	11.2			6	0.5			4	0.4		

Note. [1]If the gender variable was missing in the ABCD-SD collection, we attempted to recover the most recent response to this question from previous collections to minimize sample loss.

2. Measures information

Table S2

Translation Approaches and Psychometric Support for Assessment Instruments

Measure	Sample	Project and wave	Country	Language of administration	Translation method	Evidence of reliability and validity in target population
Child Behavior Checklist 6/16 [CBCL 6/16] (Achenbach & Rescorla, 2001)	Sample 1	ABCD, BL	US	English and Spanish	English: Original version	Achenbach & Rescorla (2001)
	Sample 2-CU and 2-ICU	ABCD-SD, BL			Spanish: Official translation	Albores-Gallo et al. (2007)
	Sample 3b	ELISA W7	Spain	Spanish/Galician	Spanish: Official translation	Lacalle et al. (2012)
Conduct Problem Scale (Colins et al., 2014)	Sample 3a	ELISA W6	Spain	Spanish/Galician	Spanish: Previously used version[a]	Fanti et al. (2023)<br>López-Romero et al. (2019)
Strengths and Difficulties Questionnaire [SDQ] (Goodman, 1997)	Sample 1	ABCD, BL	US	English and Spanish	English: Original version	He et al (2013)
	Sample 2-CU	ABCD-SD, BL			Spanish: Official translation	Venta et al. (2022)
	Sample 3b	ELISA,W7	Spain	Spanish/Galician	Spanish: Official translation	Rodríguez-Hernández et al. (2012)<br>Ortuño-Sierra et al. (2018)
Inventory of Callous Unemotional Traits (Frick, 2004)	Sample 2-ICU	ABCD-SD, BL	US	English and Spanish	English: Original version	Gao & Zhang (2016)
					Spanish: Official translation	McDonald et al. (2018)
Child Problematic Traits Inventory [CPTI] (Colins et al., 2014)	Sample 3a	ELISA, W6	Spain	Spanish/Galician	Spanish: Official translation	Barrau et al. (2022)<br>López-Romero et al. (2019)

Note. Both data from the present study (see Tables S9-S23) and previous research have shown significant correlations between the measures included. Specifically, ICU scores correlate significantly with CBCL conduct disorder symptoms (e.g., Gao & Zhang, 2016), SDQ prosocial scores (e.g., Yoshida et al., 2019), and the CU subscale of the CPTI (e.g., Figueiredo et al., 2022). Additionally, significant associations have been found between the CU subscale of the CPTI and the Conduct Problems Scale (Fanti et al., 2023), and the SDQ prosocial subscale (Barrau et al., 2022) (see Tables S9–S23 below).The ABCD study's parent questionnaires are available in both English and Spanish. Each instrument includes an initial language-selection item that determines the display language, allowing a single form to be used for both languages. In the ELISA project, parent questionnaires were available in Spanish and Galician. Galician and Spanish are co-official Romance languages in Galicia. Both evolved from Latin and exhibit strong similarities in lexical, phonological, and morphosyntactic structures (Álvarez de la Granja et al., 2025), which facilitates conceptual equivalence in translations. As both languages are used in education and daily life, participants could choose the version they felt more comfortable with. Previous use of the same instruments in both languages has shown robust psychometric properties, regardless of language (Álvarez-Voces & Romero, 2025; García et al., 2000; López-Romero et al., 2019).[a]The Spanish translation of this scale has been employed in previous studies with children of similar age and has demonstrated good psychometric properties.

Table S3

List of Items Included in the Network Analysis

Measure	Item description	Abbr	Sample
CBCL 6/16 Conduct Problems DSM-IV Oriented (Achenbach & Rescorla, 2001)	Cruel to animals	Anim	Sample 1: ABCD baseline, US<br>Sample 2-CU and 2-ICU: ABCD-SD baseline, US<br>Sample 3b: ELISA wave 7, Spain
	Cruelty, bullying or meanness to others	Pers	
	Destroys things belonging to his/her family or others	Dest	
	Breaks rules at home, school, or elsewhere	Brea	
	Gets in many fights	Figh	
	Hangs around with others who get in trouble	Hang	
	Lying or cheating	Lyin	
	Physically attacks people	Phys	
	Runs away from home	Runs	
	Sets fires	Fire	
	Steals at home	Steh	
	Steals outside the home	Steo	
	Swearing or obscene language	Swea	
	Threatens people	Thrp	
	Truancy, skips school	Trua	
	Vandalism	Vand	
Conduct Problem Scale (Colins et al., 2014)	Has teased others	Teas	Sample 3a: ELISA wave 6, Spain
	Has violated important rules	Viol	
	Has threatened someone	Thre	
	Has hit, scratched, shoved, or kicked someone or thrown something at someone, without obvious reasons	Hitw	
	Has hit, scratched, shoved, or kicked someone or thrown something at someone, when he/she has been provoked or stressed	Hitp	
	Has taken things without permission that belongs to someone else	Take	
	Has purposely broken or destroyed something	Brok	
	Has interrupted/destroyed other children's games and activities	Inte	
Previous measure CU traits (SDQ, Goodman, 1997; CBCL, Achenbach & Rescorla, 2001)	SDQ Item: Is considerate of other feelings	Cons	Sample 1: ABCD baseline, US<br>Sample 2-CU: ABCD-SD baseline, US<br>Sample 3b: ELISA wave 7, Spain[a]
	SDQ Item: Is helpful if someone is hurt or upset	Help	
	SDQ Item: Offers to help others	Offe	
	CBCL Item: Lack of guilt after misbehaving	Lack	

Table S3 (Continued)

ICU[b] (Frick, 2004)	Is concerned about schoolwork	Scho	Sample 2-ICU: ABCD-SD baseline, US
	Does not care who he/she hurts to get what he/she wants	Care	
	Feels bad or guilty when he/she has done something wrong	Bad	
	Does not care about being on time	Time	
	Does not care if he/she is in trouble	Trou	
	Does not care about doing things well	Well	
	Seems very cold and uncaring	Unca	
	Easily admits to being wrong	Wron	
	Always tries his/her best	Best	
	Apologizes ("says he/she is sorry") to persons he/she has hurt	Apol	
	Shows no remorse when he/she has done something wrong	Remo	
	Does not like to put the time into doing things well	Effo	
	The feelings of others are unimportant to him/her	Unim	
	Works hard on everything	Work	
	Does things to make others feel good	Good	
CPTI CU traits (Colins et al., 2014)	Seldom expresses sympathy for others	Symp	Sample 3a: ELISA wave 6, Spain
	Usually does not seem to share other's joy and sorrow	Shar	
	Never seems to have bad conscience for things he/she has done	Done	
	Often seems to be completely indifferent when children are upset	Indi	
	Does not become upset when others are being hurt	Upse	
	Seldom remorseful when he/she has done something not allowed	Remo	
	Often does not care about what other people feel and think	Unca	
	Sometimes seems completely lack capability feel guilt/remorse	Capa	
	Never expresses feelings of guilt when done something not allowed	Guil	
	Does not express guilt and remorse to the same extent	Expr	

Note. CBCL = Child Behavior Checklist 6/16; CP = Conduct Problems; ICU = Inventory of Callous Unemotional Traits; CPTI = Child Problematic Traits Inventory. [a]The SDQ items for the Spanish sample were obtained from wave 6. [b]Of the original 24 items in the Inventory of Callous-Unemotional Traits (ICU), only 15 were included in the parent-reported ABCD-SD. Notably, this excluded two items from the ICU callousness subscale, two items from the uncaring subscale, and all five items from the unemotional subscale. The unemotional subscale has been criticized for its comparatively lower predictive and construct validity compared to items assessing callousness or uncaring (Kimonis et al., 2013).

3. Analysis of missing data

Table S4

Missing data in Sample 1: ABCD baseline, US

Total records = 11,868	*n* of missing	Rate (%)
Cruel to animals	7	0.1
Cruelty, bullying or meanness to others	7	0.1
Destroys things belonging to his/her family or others	8	0.1
Breaks rules at home, school, or elsewhere	7	0.1
Gets in many fights	7	0.1
Hangs around with others who get in trouble	7	0.1
Lying or cheating	7	0.1
Physically attacks people	7	0.1
Runs away from home	7	0.1
Sets fires	7	0.1
Steals at home	7	0.1
Steals outside the home	7	0.1
Swearing or obscene language	7	0.1
Threatens people	7	0.1
Truancy, skips school	7	0.1
Vandalism	7	0.1
Lack of guilt after misbehaving	7	0.1
Is considerate of other feelings	32	0.3
Is helpful if someone is hurt or upset	32	0.3
Offers to help others	46	0.4
Incomplete records	73	0.6
Records with missing in all variables	0	0

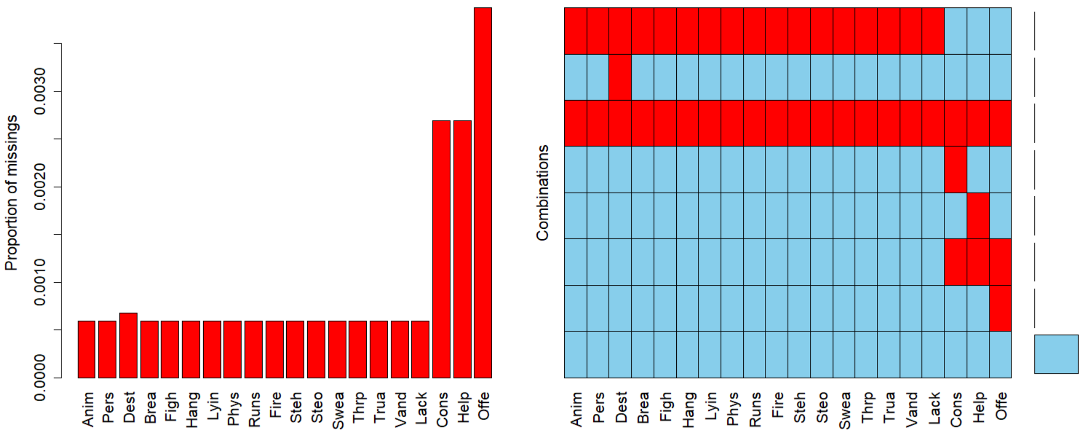

Note. The percentage of missing values across the 20 variables varied between 0 and 0.4%. In total 73 out 11,868 records (0.6%) were incomplete. Little's test (Little, 1988) was performed to assess whether the missing data was completely at random (MCAR). The results indicated that the missing data were not consistent with the MCAR assumption ($\chi^2(132)=210$, $p<.001$). However, the normed χ^2 was 1.59, which is below the recommended threshold of 2 (Ullman et al., 2001), suggesting that the data were at least missing at random (MAR).

Table S5

Missing data in Sample 2-ICU: ABCD-SD baseline, US

Total records = 2,426	*n* of missing	Rate (%)
Cruel to animals	89	3.7
Cruelty, bullying or meanness to others	89	3.7
Destroys things belonging to his/her family or others	89	3.7
Breaks rules at home, school, or elsewhere	89	3.7
Gets in many fights	89	3.7
Hangs around with others who get in trouble	89	3.7
Lying or cheating	89	3.7
Physically attacks people	89	3.7
Runs away from home	89	3.7
Sets fires	89	3.7
Steals at home	89	3.7
Steals outside the home	89	3.7
Swearing or obscene language	89	3.7
Threatens people	89	3.7
Truancy, skips school	89	3.7
Vandalism	89	3.7
Is concerned about schoolwork	4	0.2
Does not care who he/she hurts to get what he/she wants	5	0.2
Feels bad or guilty when he/she has done something wrong	6	0.2
Does not care about being on time	7	0.3
Does not care if he/she is in trouble	6	0.2
Does not care about doing things well	4	0.2
Seems very cold and uncaring	4	0.2
Easily admits to being wrong	6	0.2
Always tries his/her best	5	0.2
Apologizes to persons he/she has hurt	6	0.2
Shows no remorse when he/she has done something wrong	5	0.2
Does not like to put the time into doing things well	8	0.3
The feelings of others are unimportant to him/her	7	0.3
Works hard on everything	4	0.2
Does things to make others feel good	6	0.2
Incomplete records	109	4.5
Records with missing in all variables	0	0

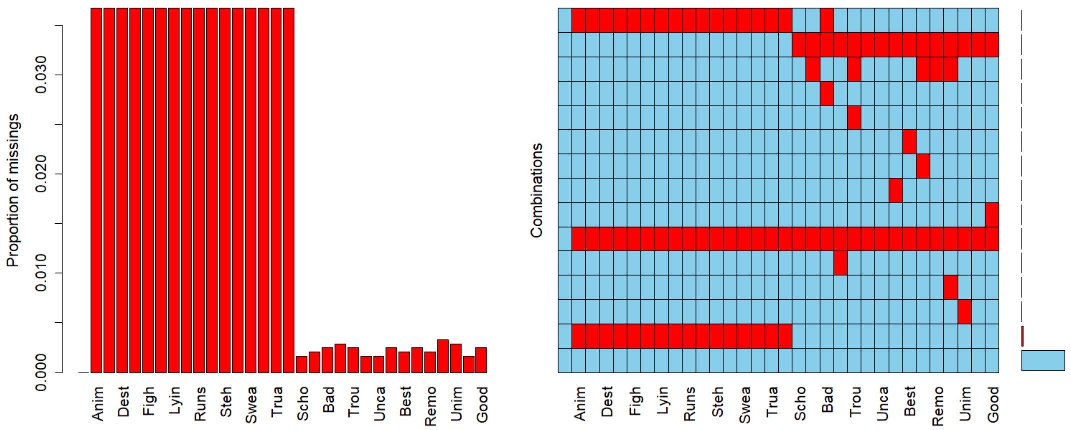

Note. The percentage of missing values across the 31 variables varied between 0.2 and 3.7%. In total 109 out 2,426 records (4.5%) were incomplete. Little's test (Little, 1988) was performed to assess whether the missing data was completely at random (MCAR). The results indicated that the missing data were not consistent with the MCAR assumption ($\chi^2(369)=447$, $p=.01$). However, the normed χ^2 was 1.21, which is below the recommended threshold of 2 (Ullman et al., 2001), suggesting that the data were at least missing at random (MAR).

Table S6

Missing data in Sample 2-CU: ABCD-SD baseline, US

Total records = 2,426	*n* of missing	Rate (%)
Cruel to animals	89	3.7
Cruelty, bullying or meanness to others	89	3.7
Destroys things belonging to his/her family or others	89	3.7
Breaks rules at home, school, or elsewhere	89	3.7
Gets in many fights	89	3.7
Hangs around with others who get in trouble	89	3.7
Lying or cheating	89	3.7
Physically attacks people	89	3.7
Runs away from home	89	3.7
Sets fires	89	3.7
Steals at home	89	3.7
Steals outside the home	89	3.7
Swearing or obscene language	89	3.7
Threatens people	89	3.7
Truancy, skips school	89	3.7
Vandalism	89	3.7
Lack of guilt after misbehaving	89	3.7
Is considerate of other feelings	92	3.8
Is helpful if someone is hurt or upset	92	3.8
Offers to help others	92	3.8
Incomplete records	92	3.8
Records with missing in all variables	0	0

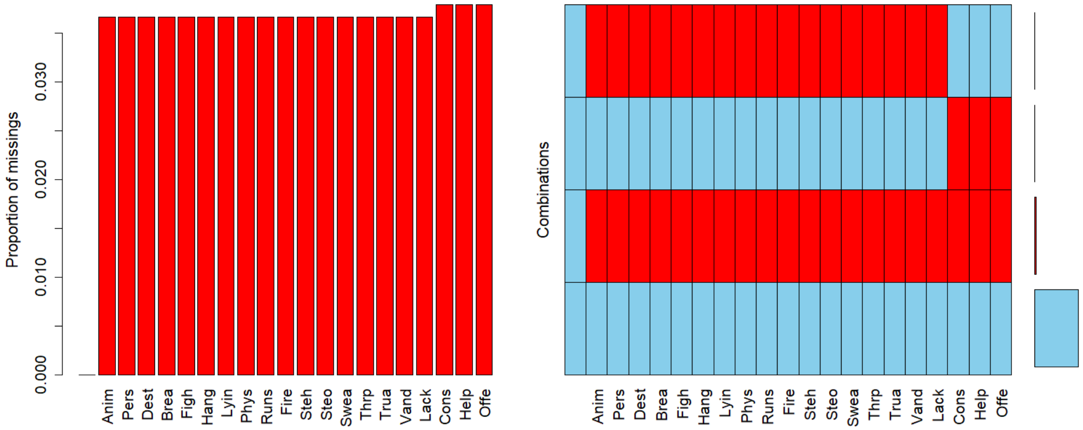

Note. The percentage of missing values across the 20 variables varied between 3.7 and 3.8%. In total 92 out 2,426 records (3.8%) were incomplete. Little's test (Little, 1988) was performed to assess whether the missing data was completely at random (MCAR). The results indicated that the missing data were consistent with the MCAR assumption, ($\chi^2(26)$=27.7, p=.37).

Table S7

Missing data in Sample 3a: ELISA wave 6, Spain

Total records = 1,342	*n* of missing	Rate (%)
Has teased others	36	2.7
Has violated important rules	40	3
Has threatened someone	38	2.8
Has hit, scratched, shoved, or kicked someone or thrown something at someone, without obvious reasons	37	2.8
Has hit, scratched, shoved, or kicked someone or thrown something at someone, when he/she has been provoked or stressed	37	2.8
Has taken things without permission that belongs to someone else	37	2.8
Has purposely broken or destroyed something	38	2.8
Has interrupted/destroyed other children's games and activities	38	2.8
Seldom expresses sympathy for others	28	2.1
Usually does not seem to share other's joy and sorrow	29	2.2
Never seems to have bad conscience for things he/she has done	31	2.3
Often seems to be completely indifferent when children are upset	32	2.4
Does not become upset when others are being hurt	30	2.2
Seldom remorseful when he/she has done something not allowed	30	2.2
Often does not care about what other people feel and think	29	2.2
Sometimes seems completely lack capability feel guilt/remorse	28	2.1
Never expresses feelings of guilt when done something not allowed	29	2.2
Does not express guilt and remorse to the same extent	28	2.1
Incomplete records	85	6.3
Records with missing in all variables	0	0

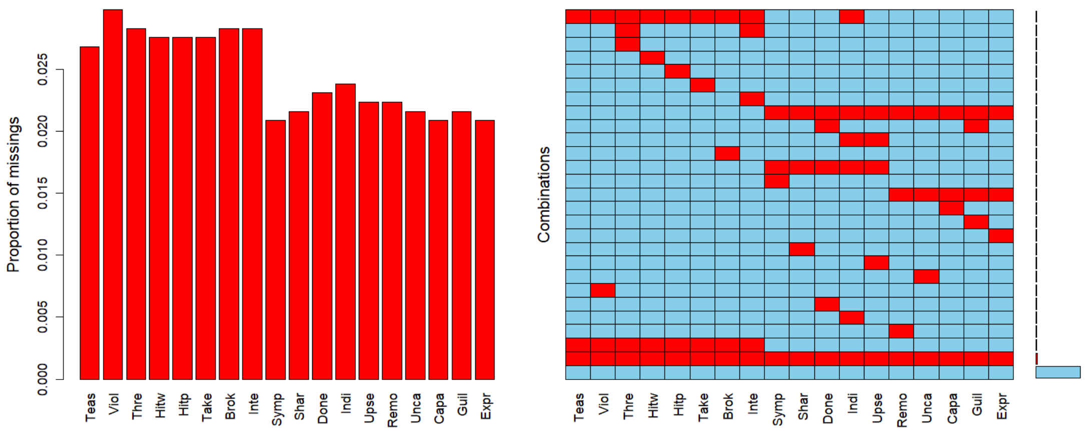

Note. The percentage of missing values across the 18 variables varied between 2.1 and 3%. In total 85 out 1,342 records (6.3%) were incomplete. Little's test (Little, 1988) was performed to assess whether the missing data was completely at random (MCAR). The results indicated that the missing data were consistent with the MCAR assumption, ($\chi^2(442)=344$, $p=1$).

Table S8

Missing data in Sample 3b: ELISA wave 7, Spain

Total records = 1,259	*n* of missing	Rate (%)
Cruel to animals	12	0.9
Cruelty, bullying or meanness to others	6	0.5
Destroys things belonging to his/her family or others	3	0.2
Breaks rules at home, school, or elsewhere	4	0.3
Gets in many fights	13	1
Hangs around with others who get in trouble	8	0.6
Lying or cheating	8	0.6
Physically attacks people	13	1
Runs away from home	13	1
Sets fires	14	1.1
Steals at home	9	0.7
Steals outside the home	15	1.2
Swearing or obscene language	9	0.7
Threatens people	14	1.1
Truancy, skips school	11	0.9
Vandalism	9	0.7
Lack of guilt after misbehaving	4	0.3
Is considerate of other feelings	314	24.9
Is helpful if someone is hurt or upset	312	24.8
Offers to help others	317	25.2
Incomplete records	397	31.5
Records with missing in all variables	0	0

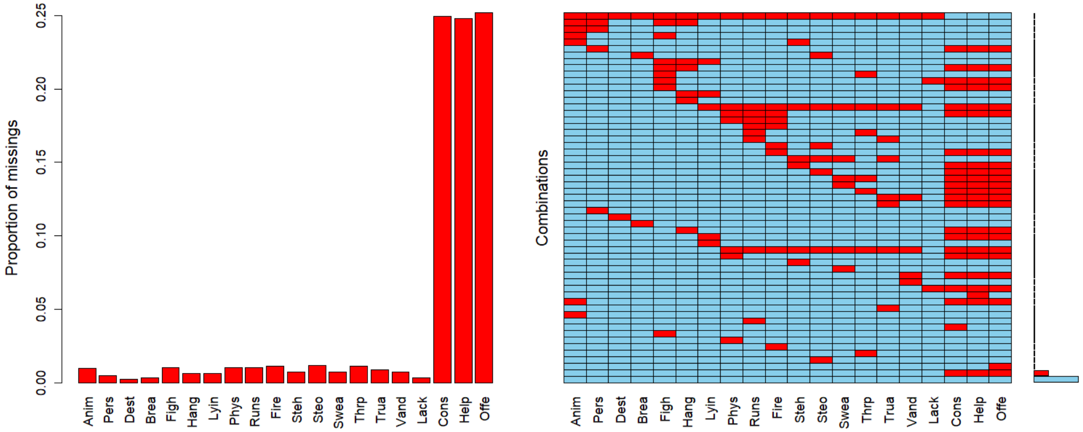

Note. The percentage of missing values across the 20 variables varied between .2 and 25.2%. In total 392 out 1,260 records (31.1%) were incomplete. Little's test (Little, 1988) was performed to assess whether the missing data was completely at random (MCAR). The results indicated that the missing data were consistent with the MCAR assumption, (χ^2(1022)=1170, *p*=1).

4. Spearman correlations of the study variables

Table S9

Spearman Correlations Sample 1 Full: ABCD baseline, US

	Anim	Pers	Dest	Brea	Figh	Hang	Lyin	Phys	Runs	Fire	Steh	Steo	Swea	Thrp	Trua	Vand	Lack	Cons	Help	Offe
Anim																				
Pers	.21[a]																			
Dest	.23[a]	.37[a]																		
Brea	.13[a]	.36[a]	.42[a]																	
Figh	.15[a]	.32[a]	.35[a]	.32[a]																
Hang	.08[a]	.26[a]	.22[a]	.29[a]	.29[a]															
Lyin	.14[a]	.31[a]	.35[a]	.52[a]	.26[a]	.26[a]														
Phys	.17[a]	.40[a]	.40[a]	.33[a]	.40[a]	.16[a]	.26[a]													
Runs	.14[a]	.13[a]	.15[a]	.15[a]	.17[a]	.06[a]	.11[a]	.17[a]												
Fire	.11[a]	.10[a]	.12[a]	.11[a]	.08[a]	.10[a]	.13[a]	.08[a]	.06[a]											
Steh	.19[a]	.26[a]	.37[a]	.35[a]	.23[a]	.18[a]	.37[a]	.21[a]	.15[a]	.16[a]										
Steo	.18[a]	.19[a]	.30[a]	.25[a]	.25[a]	.18[a]	.30[a]	.17[a]	.14[a]	.19[a]	.53[a]									
Swea	.13[a]	.29[a]	.31[a]	.32[a]	.25[a]	.23[a]	.24[a]	.27[a]	.13[a]	.12[a]	.23[a]	.19[a]								
Thrp	.18[a]	.42[a]	.39[a]	.32[a]	.38[a]	.19[a]	.25[a]	.45[a]	.20[a]	.10[a]	.29[a]	.23[a]	.33[a]							
Trua	.02[c]	.09[a]	.10[a]	.07[a]	.08[a]	.03[b]	.06[a]	.11[a]	.12[a]	.03[b]	.05[a]	.06[a]	.11[a]	.11[a]						
Vand	.17[a]	.22[a]	.34[a]	.21[a]	.23[a]	.19[a]	.19[a]	.20[a]	.16[a]	.13[a]	.29[a]	.26[a]	.20[a]	.24[a]	.11[a]					
Lack	.13[a]	.35[a]	.35[a]	.45[a]	.26[a]	.21[a]	.39[a]	.27[a]	.12[a]	.11[a]	.27[a]	.21[a]	.24[a]	.27[a]	.07[a]	.21[a]				
Cons	.13[a]	.31[a]	.26[a]	.31[a]	.21[a]	.15[a]	.27[a]	.23[a]	.09[a]	.05[a]	.17[a]	.14[a]	.20[a]	.23[a]	.04[a]	.13[a]	.34[a]			
Help	.09[a]	.20[a]	.16[a]	.18[a]	.13[a]	.09[a]	.14[a]	.14[a]	.07[a]	.04[a]	.09[a]	.08[a]	.12[a]	.16[a]	.03[b]	.10[a]	.23[a]	.59[a]		
Offe	.07[a]	.17[a]	.13[a]	.18[a]	.10[a]	.06[a]	.14[a]	.14[a]	.06[a]	.03[b]	.08[a]	.05[a]	.13[a]	.14[a]	.03[b]	.08[a]	.21[a]	.54[a]	.59[a]	

Note. [a]$p < .001$; [b]$p < .01$; [c]$p < .05$. There was a significant positive Pearson correlation between the total CBCL conduct problems score and the total CU score, $r = .49$, $p < .001$.

Table S10

Spearman Correlations Sample 1 Boys: ABCD baseline, US

	Anim	Pers	Dest	Brea	Figh	Hang	Lyin	Phys	Runs	Fire	Steh	Steo	Swea	Thrp	Trua	Vand	Lack	Cons	Help	Offe
Anim																				
Pers	.22[a]																			
Dest	.25[a]	.37[a]																		
Brea	.13[a]	.37[a]	.44[a]																	
Figh	.15[a]	.34[a]	.36[a]	.34[a]																
Hang	.08[a]	.26[a]	.22[a]	.31[a]	.29[a]															
Lyin	.15[a]	.32[a]	.35[a]	.51[a]	.28[a]	.29[a]														
Phys	.17[a]	.41[a]	.42[a]	.34[a]	.40[a]	.17[a]	.27[a]													
Runs	.15[a]	.14[a]	.17[a]	.16[a]	.19[a]	.07[a]	.12[a]	.18[a]												
Fire	.11[a]	.10[a]	.13[a]	.12[a]	.07[a]	.12[a]	.14[a]	.11[a]	.07[a]											
Steh	.21[a]	.28[a]	.38[a]	.35[a]	.24[a]	.21[a]	.38[a]	.23[a]	.19[a]	.17[a]										
Steo	.18[a]	.20[a]	.29[a]	.25[a]	.27[a]	.21[a]	.31[a]	.19[a]	.16[a]	.20[a]	.55[a]									
Swea	.10[a]	.29[a]	.34[a]	.33[a]	.25[a]	.24[a]	.26[a]	.28[a]	.15[a]	.11[a]	.26[a]	.19[a]								
Thrp	.18[a]	.45[a]	.41[a]	.34[a]	.38[a]	.20[a]	.28[a]	.47[a]	.22[a]	.12[a]	.33[a]	.26[a]	.34[a]							
Trua	.04[b]	.08[a]	.09[a]	.08[a]	.09[a]	.04[b]	.06[a]	.10[a]	.14[a]	.05[a]	.07[a]	.07[a]	.10[a]	.12[a]						
Vand	.16[a]	.24[a]	.36[a]	.22[a]	.24[a]	.20[a]	.21[a]	.20[a]	.16[a]	.14[a]	.32[a]	.27[a]	.21[a]	.24[a]	.16[a]					
Lack	.14[a]	.35[a]	.35[a]	.45[a]	.27[a]	.21[a]	.38[a]	.27[a]	.14[a]	.11[a]	.26[a]	.20[a]	.24[a]	.28[a]	.06[a]	.23[a]				
Cons	.13[a]	.31[a]	.27[a]	.31[a]	.23[a]	.15[a]	.26[a]	.24[a]	.09[a]	.05[a]	.19[a]	.14[a]	.20[a]	.25[a]	.03[b]	.15[a]	.35[a]			
Help	.11[a]	.22[a]	.16[a]	.19[a]	.15[a]	.10[a]	.15[a]	.15[a]	.07[a]	.03[b]	.12[a]	.09[a]	.13[a]	.19[a]	.02[c]	.12[a]	.25[a]	.60[a]		
Offe	.08[a]	.19[a]	.13[a]	.18[a]	.10[a]	.06[a]	.15[a]	.14[a]	.05[a]	.01	.09[a]	.05[a]	.13[a]	.16[a]	.01	.09[a]	.21[a]	.53[a]	.59[a]	

Note. [a]$p < .001$; [b]$p < .01$; [c]$p < .05$. There was a significant positive Pearson correlation between the total CBCL conduct problems score and the total CU score, $r = .49$, $p < .001$.

Table S11

Spearman Correlations Sample 1 Girls: ABCD baseline, US

	Anim	Pers	Dest	Brea	Figh	Hang	Lyin	Phys	Runs	Fire	Steh	Steo	Swea	Thrp	Trua	Vand	Lack	Cons	Help	Offe
Anim																				
Pers	.20[a]																			
Dest	.19[a]	.35[a]																		
Brea	.12[a]	.34[a]	.39[a]																	
Figh	.14[a]	.29[a]	.31[a]	.28[a]																
Hang	.09[a]	.24[a]	.19[a]	.23[a]	.26[a]															
Lyin	.12[a]	.29[a]	.34[a]	.51[a]	.21[a]	.21[a]														
Phys	.15[a]	.37[a]	.36[a]	.30[a]	.37[a]	.12[a]	.22[a]													
Runs	.09[a]	.11[a]	.10[a]	.11[a]	.11[a]	.02	.08[a]	.14[a]												
Fire	.11[a]	.09[a]	.10[a]	.08[a]	.11[a]	.03c	.11[a]	.02	.06[a]											
Steh	.16[a]	.23[a]	.37[a]	.35[a]	.21[a]	.14[a]	.37[a]	.20[a]	.10[a]	.16[a]										
Steo	.17[a]	.17[a]	.31[a]	.25[a]	.23[a]	.13[a]	.28[a]	.16[a]	.11[a]	.17[a]	.50[a]									
Swea	.18[a]	.29[a]	.25[a]	.27[a]	.24[a]	.18[a]	.20[a]	.21[a]	.07[a]	.13[a]	.21[a]	.22[a]								
Thrp	.16[a]	.36[a]	.35[a]	.27[a]	.37[a]	.15[a]	.21[a]	.41[a]	.12[a]	.05[a]	.24[a]	.20[a]	.29[a]							
Trua	0	.09[a]	.12[a]	.06[a]	.04[b]	.01	.07[a]	.13[a]	.11[a]	0	.03[c]	.05[a]	.13[a]	.10[a]						
Vand	.20[a]	.18[a]	.29[a]	.17[a]	.21[a]	.14[a]	.15[a]	.21[a]	.17[a]	.07[a]	.27[a]	.26[a]	.15[a]	.25[a]	.03[c]					
Lack	.10[a]	.34[a]	.34[a]	.45[a]	.25[a]	.21[a]	.38[a]	.27[a]	.08[a]	.09[a]	.28[a]	.21[a]	.22[a]	.25[a]	.08[a]	.17[a]				
Cons	.10[a]	.29[a]	.24[a]	.28[a]	.16[a]	.14[a]	.28[a]	.20[a]	.07[a]	.05[a]	.16[a]	.15[a]	.18[a]	.18[a]	.04[b]	.10[a]	.32[a]			
Help	.05[b]	.15[a]	.14[a]	.14[a]	.06[a]	.04[b]	.11[a]	.10[a]	.05[a]	.03[c]	.05[a]	.05[a]	.08[a]	.11[a]	.04[b]	.04[b]	.19[a]	.56[a]		
Offe	.05[a]	.12[a]	.11[a]	.14[a]	.07[a]	.03[c]	.12[a]	.11[a]	.07[a]	.03[c]	.05[a]	.05[a]	.09[a]	.08[a]	.05[a]	.06[a]	.18[a]	.53[a]	.58[a]	

Note. [a]*p* <.001; [b]*p* < .01; [c]*p* < .05. There was a significant positive Pearson correlation between the total CBCL conduct problems score and the total CU score, *r* = .46, *p* < .001.

Table S12

Spearman Correlations Sample 2-ICU Full: ABCD-SD baseline, US

	Anim	Pers	Dest	Brea	Figh	Hang	Lyin	Phys	Runs	Fire	Steh	Steo	Swa	Thrp	Trua	Vand	Scho	Care	Bad	Time	Trou	Well	Unca	Wrong	Best	Apol	Remo	Effo	Unim	Work	Good
Anim																															
Pers	.28[a]																														
Dest	.28[a]	.36[a]																													
Brea	.18[a]	.34[a]	.42[a]																												
Figh	.17[a]	.40[a]	.34[a]	.32[a]																											
Hang	.14[a]	.30[a]	.23[a]	.32[a]	.41[a]																										
Lyin	.15[a]	.32[a]	.33[a]	.52[a]	.25[a]	.28[a]																									
Phys	.16[a]	.37[a]	.40[a]	.31[a]	.38[a]	.26[a]	.25[a]																								
Runs	.03	.21[a]	.30[a]	.20[a]	.32[a]	.16[a]	.15[a]	.34[a]																							
Fire	.10[a]	.14[a]	.16[a]	.15[a]	.22[a]	.16[a]	.13[a]	.14[a]	.21[a]																						
Steh	.12[a]	.24[a]	.41[a]	.32[a]	.27[a]	.21[a]	.38[a]	.31[a]	.24[a]	.20[a]																					
Steo	.16[a]	.16[a]	.34[a]	.26[a]	.22[a]	.18[a]	.28[a]	.30[a]	.33[a]	.30[a]	.55[a]																				
Swea	.16[a]	.29[a]	.37[a]	.33[a]	.28[a]	.18[a]	.28[a]	.29[a]	.21[a]	.14[a]	.25[a]	.19[a]																			
Thrp	.18[a]	.42[a]	.41[a]	.36[a]	.50[a]	.27[a]	.28[a]	.51[a]	.26[a]	.20[a]	.32[a]	.31[a]	.37[a]																		
Trua	-.01	.09[a]	.13[a]	.15[a]	.13[a]	.08[a]	.11[a]	.12[a]	.25[a]	.08[a]	.12[a]	.12[a]	.11[a]	.18[a]																	
Vand	.07[b]	.25[a]	.32[a]	.22[a]	.38[a]	.18[a]	.18[a]	.31[a]	.49[a]	.21[a]	.28[a]	.34[a]	.25[a]	.35[a]	.35[a]																
Scho	.04[c]	.13[a]	.15[a]	.28[a]	.13[a]	.13[a]	.26[a]	.14[a]	.10[a]	.08[a]	.11[a]	.11[a]	.19[a]	.14[a]	.08[a]	.11[a]															
Care	.11[a]	.27[a]	.26[a]	.28[a]	.19[a]	.18[a]	.26[a]	.22[a]	.15[a]	.10[a]	.19[a]	.16[a]	.19[a]	.25[a]	.12[a]	.22[a]	.22[a]														
Bad	.07[a]	.23[a]	.22[a]	.29[a]	.16[a]	.16[a]	.26[a]	.18[a]	.12[a]	.09[a]	.13[a]	.13[a]	.17[a]	.17[a]	.07[a]	.12[a]	.41[a]	.32[a]													
Time	.04	.10[a]	.14[a]	.17[a]	.09[a]	.09[a]	.20[a]	.12[a]	.13[a]	.08[a]	.14[a]	.13[a]	.15[a]	.11[a]	.10[a]	.11[a]	.32[a]	.15[a]	.16[a]												
Trou	.09[a]	.21[a]	.26[a]	.34[a]	.20[a]	.22[a]	.27[a]	.19[a]	.13[a]	.08[a]	.20[a]	.16[a]	.23[a]	.22[a]	.12[a]	.14[a]	.29[a]	.35[a]	.39[a]	.26[a]											
Well	.04	.13[a]	.15[a]	.24[a]	.12[a]	.15[a]	.25[a]	.12[a]	.11[a]	.10[a]	.13[a]	.13[a]	.16[a]	.13[a]	.11[a]	.09[a]	.47[a]	.24[a]	.27[a]	.30[a]	.35[a]										
Unca	.11[a]	.25[a]	.20[a]	.24[a]	.19[a]	.14[a]	.21[a]	.18[a]	.11[a]	.02	.16[a]	.10[a]	.17[a]	.22[a]	.10[a]	.16[a]	.21[a]	.48[a]	.28[a]	.19[a]	.36[a]	.26[a]									
Wron	.07[b]	.19[a]	.19[a]	.28[a]	.09[a]	.12[a]	.28[a]	.14[a]	.07[a]	.06[b]	.15[a]	.12[a]	.14[a]	.11[a]	.05[c]	.07[a]	.36[a]	.25[a]	.54[a]	.20[a]	.31[a]	.23[a]	.24[a]								
Best	.03	.15[a]	.17[a]	.29[a]	.11[a]	.11[a]	.28[a]	.13[a]	.07[b]	.06[b]	.13[a]	.08[a]	.19[a]	.13[a]	.08[a]	.10[a]	.69[a]	.19[a]	.40[a]	.31[a]	.25[a]	.47[a]	.21[a]	.40[a]							
Apol	.09[a]	.22[a]	.17[a]	.23[a]	.12[a]	.12[a]	.20[a]	.16[a]	.11[a]	.08[a]	.11[a]	.09[a]	.13[a]	.15[a]	.06[b]	.10[a]	.37[a]	.28[a]	.69[a]	.18[a]	.28[a]	.25[a]	.27[a]	.55[a]	.40[a]						
Remo	.07[b]	.16[a]	.17[a]	.22[a]	.15[a]	.15[a]	.19[a]	.16[a]	.09[a]	.01	.12[a]	.08[a]	.10[a]	.13[a]	.07[b]	.10[a]	.18[a]	.28[a]	.31[a]	.16[a]	.37[a]	.23[a]	.27[a]	.21[a]	.16[a]	.22[a]					
Effo	.05[c]	.16[a]	.17[a]	.27[a]	.11[a]	.13[a]	.30[a]	.15[a]	.09[a]	.07[b]	.14[a]	.12[a]	.17[a]	.15[a]	.09[a]	.08[a]	.47[a]	.21[a]	.25[a]	.36[a]	.29[a]	.54[a]	.25[a]	.31[a]	.56[a]	.27[a]	.21[a]				
Unim	.07[b]	.13[a]	.13[a]	.12[a]	.10[a]	.11[a]	.10[a]	.10[a]	.08[a]	.02	.08[a]	.08[a]	.08[a]	.14[a]	.04[c]	.11[a]	.12[a]	.24[a]	.12[a]	.09[a]	.19[a]	.16[a]	.30[a]	.08[a]	.10[a]	.12[a]	.18[a]	.14[a]			
Work	.06[b]	.17[a]	.17[a]	.30[a]	.11[a]	.13[a]	.30[a]	.14[a]	.08[a]	.06[b]	.12[a]	.10[a]	.19[a]	.13[a]	.08[a]	.10[a]	.69[a]	.20[a]	.41[a]	.32[a]	.25[a]	.45[a]	.21[a]	.41[a]	.85[a]	.40[a]	.17[a]	.55[a]	.11[a]		
Good	.08[a]	.21[a]	.18[a]	.21[a]	.13[a]	.13[a]	.18[a]	.15[a]	.08[a]	.08[a]	.08[a]	.08[a]	.16[a]	.14[a]	.05[b]	.09[a]	.36[a]	.23[a]	.52[a]	.16[a]	.26[a]	.23[a]	.26[a]	.44[a]	.41[a]	.55[a]	.17[a]	.25[a]	.12[a]	.41[a]	

Note. [a]p <.001; [b]p < .01; [c]p < .05. There was a significant positive Pearson correlation between the total CBCL conduct problems score and the total ICU score, r = .63, p < .001.

Table S13

Spearman Correlations Sample 2-ICU Boys: ABCD-SD baseline, US

	Anim	Pers	Dest	Brea	Figh	Hang	Lyin	Phys	Runs	Fire	Steh	Steo	Swa	Thrp	Trua	Vand	Scho	Care	Bad	Time	Trou	Well	Unca	Wrong	Best	Apol	Remo	Effo	Unim	Work	Good
Anim																															
Pers	.33[a]																														
Dest	.26[a]	.38[a]																													
Brea	.15[a]	.32[a]	.41[a]																												
Figh	.13[a]	.40[a]	.33[a]	.30[a]																											
Hang	.06[c]	.25[a]	.20[a]	.30[a]	.30[a]																										
Lyin	.12[a]	.32[a]	.29[a]	.50[a]	.24[a]	.27[a]																									
Phys	.15[a]	.40[a]	.40[a]	.30[a]	.37[a]	.23[a]	.26[a]																								
Runs	-.01	.30[a]	.29[a]	.20[a]	.40[a]	.16[a]	.15[a]	.29[a]																							
Fire	.04	.12[a]	.18[a]	.17[a]	.22[a]	.15[a]	.15[a]	.19[a]	.18[a]																						
Steh	.08[b]	.26[a]	.39[a]	.31[a]	.29[a]	.17[a]	.36[a]	.29[a]	.21[a]	.28[a]																					
Steo	.07[b]	.20[a]	.30[a]	.26[a]	.26[a]	.18[a]	.27[a]	.29[a]	.30[a]	.43[a]	.56[a]																				
Swea	.17[a]	.35[a]	.38[a]	.35[a]	.30[a]	.17[a]	.31[a]	.29[a]	.26[a]	.16[a]	.30[a]	.18[a]																			
Thrp	.18[a]	.47[a]	.45[a]	.37[a]	.47[a]	.25[a]	.30[a]	.55[a]	.36[a]	.21[a]	.36[a]	.40[a]	.39[a]																		
Trua	-.01	.11[a]	.14[a]	.16[a]	.14[a]	.02	.11[a]	.10[a]	.36[a]	.11[a]	.14[a]	.14[a]	.12[a]	.22[a]																	
Vand	.04	.33[a]	.36[a]	.25[a]	.47[a]	.22[a]	.19[a]	.33[a]	.65[a]	.26[a]	.31[a]	.35[a]	.30[a]	.41[a]	.37[a]																
Scho	.01	.15[a]	.14[a]	.28[a]	.15[a]	.15[a]	.26[a]	.15[a]	.08[b]	.10[a]	.11[a]	.08[b]	.19[a]	.17[a]	.07[c]	.10[a]															
Care	.12[a]	.32[a]	.28[a]	.27[a]	.22[a]	.18[a]	.28[a]	.25[a]	.20[a]	.12[a]	.16[a]	.17[a]	.19[a]	.30[a]	.15[a]	.26[a]	.21[a]														
Bad	.07[c]	.23[a]	.20[a]	.29[a]	.15[a]	.17[a]	.25[a]	.19[a]	.14[a]	.10[b]	.11[a]	.13[a]	.17[a]	.19[a]	.08[b]	.14[a]	.38[a]	.34[a]													
Time	.01	.13[a]	.15[a]	.16[a]	.11[a]	.09[b]	.22[a]	.12[a]	.14[a]	.10[a]	.17[a]	.13[a]	.17[a]	.14[a]	.11[a]	.12[a]	.33[a]	.18[a]	.19[a]												
Trou	.09[b]	.22[a]	.26[a]	.34[a]	.19[a]	.22[a]	.26[a]	.19[a]	.14[a]	.12[a]	.16[a]	.15[a]	.23[a]	.25[a]	.13[a]	.15[a]	.28[a]	.37[a]	.42[a]	.28[a]											
Well	.01	.12[a]	.14[a]	.24[a]	.12[a]	.15[a]	.26[a]	.11[a]	.09[b]	.10[a]	.11[a]	.11[a]	.16[a]	.14[a]	.10[a]	.08[b]	.48[a]	.26[a]	.27[a]	.34[a]	.36[a]										
Unca	.14[a]	.29[a]	.26[a]	.26[a]	.23[a]	.17[a]	.22[a]	.20[a]	.18[a]	.04	.15[a]	.10[a]	.21[a]	.26[a]	.11[a]	.18[a]	.21[a]	.49[a]	.32[a]	.20[a]	.37[a]	.28[a]									
Wron	.07[c]	.19[a]	.19[a]	.29[a]	.09[b]	.12[a]	.30[a]	.14[a]	.08[b]	.08[b]	.14[a]	.12[a]	.14[a]	.14[a]	.04	.08[b]	.35[a]	.28[a]	.53[a]	.23[a]	.33[a]	.25[a]	.25[a]								
Best	.02	.17[a]	.17[a]	.27[a]	.09[b]	.11[a]	.30[a]	.14[a]	.08[b]	.08[b]	.12[a]	.07[c]	.19[a]	.14[a]	.07[b]	.10[a]	.68[a]	.18[a]	.37[a]	.32[a]	.25[a]	.47[a]	.21[a]	.41[a]							
Apol	.09[b]	.23[a]	.17[a]	.24[a]	.11[a]	.14[a]	.21[a]	.17[a]	.11[a]	.10[b]	.11[a]	.09[b]	.12[a]	.16[a]	.07[c]	.12[a]	.37[a]	.29[a]	.68[a]	.20[a]	.31[a]	.26[a]	.29[a]	.53[a]	.40[a]						
Remo	.08[b]	.19[a]	.18[a]	.24[a]	.14[a]	.17[a]	.20[a]	.15[a]	.10[a]	.04	.08[b]	.07[c]	.11[a]	.13[a]	.09[b]	.12[a]	.23[a]	.35[a]	.36[a]	.21[a]	.43[a]	.29[a]	.33[a]	.27[a]	.20[a]	.27[a]					
Effo	.01	.16[a]	.16[a]	.27[a]	.10[a]	.13[a]	.31[a]	.15[a]	.08[b]	.07[c]	.15[a]	.10[a]	.18[a]	.17[a]	.09[b]	.06[c]	.49[a]	.20[a]	.24[a]	.37[a]	.30[a]	.57[a]	.29[a]	.34[a]	.58[a]	.29[a]	.26[a]				
Unim	.06[c]	.16[a]	.13[a]	.12[a]	.11[a]	.11[a]	.10[a]	.11[a]	.11[a]	.04	.08[b]	.09[b]	.10[b]	.18[a]	.06[c]	.11[a]	.13[a]	.27[a]	.13[a]	.08[b]	.17[a]	.17[a]	.29[a]	.07[c]	.11[a]	.12[a]	.20[a]	.15[a]			
Work	.05	.19[a]	.16[a]	.29[a]	.11[a]	.14[a]	.32[a]	.17[a]	.09[b]	.07[c]	.13[a]	.09[b]	.21[a]	.16[a]	.07[b]	.11[a]	.68[a]	.19[a]	.39[a]	.34[a]	.25[a]	.44[a]	.20[a]	.42[a]	.84[a]	.40[a]	.20[a]	.56[a]	.10[a]		
Good	.10[a]	.23[a]	.20[a]	.25[a]	.11[a]	.14[a]	.20[a]	.18[a]	.10[a]	.09[b]	.11[a]	.12[a]	.17[a]	.17[a]	.05	.13[a]	.37[a]	.25[a]	.51[a]	.20[a]	.29[a]	.22[a]	.28[a]	.46[a]	.41[a]	.56[a]	.20[a]	.27[a]	.13[a]	.40[a]	

Note. $^{a}p < .001$; $^{b}p < .01$; $^{c}p < .05$. There was a significant positive Pearson correlation between the total CBCL conduct problems score and the total ICU score, $r = .63$, $p < .001$.

Table S14

Spearman Correlations Sample 2-ICU Girls: ABCD-SD baseline, US

	Anim	Pers	Dest	Brea	Figh	Hang	Lyin	Phys	Runs	Fire	Steh	Steo	Swa	Thrp	Trua	Vand	Scho	Care	Bad	Time	Trou	Well	Unca	Wrong	Best	Apol	Remo	Effo	Unim	Work	Good
Anim																															
Pers	.20[a]																														
Dest	.32[a]	.31[a]																													
Brea	.22[a]	.35[a]	.42[a]																												
Figh	.22[a]	.41[a]	.35[a]	.34[a]																											
Hang	.26[a]	.38[a]	.30[a]	.37[a]	.56[a]																										
Lyin	.19[a]	.32[a]	.39[a]	.55[a]	.27[a]	.30[a]																									
Phys	.18[a]	.31[a]	.38[a]	.32[a]	.42[a]	.31[a]	.21[a]																								
Runs	.08[b]	.09[b]	.32[a]	.21[a]	.21[a]	.15[a]	.14[a]	.42[a]																							
Fire	.26[a]	.19[a]	.10[b]	.11[a]	.24[a]	.18[a]	.09[b]	-.01	.28[a]																						
Steh	.17[a]	.22[a]	.46[a]	.36[a]	.26[a]	.25[a]	.43[a]	.37[a]	.27[a]	.09[b]																					
Steo	.28[a]	.12[a]	.40[a]	.28[a]	.19[a]	.19[a]	.30[a]	.32[a]	.36[a]	.15[a]	.54[a]																				
Swea	.15[a]	.19[a]	.34[a]	.28[a]	.24[a]	.20[a]	.22[a]	.29[a]	.14[a]	.08[b]	.22[a]	.24[a]																			
Thrp	.19[a]	.32[a]	.32[a]	.33[a]	.57[a]	.33[a]	.24[a]	.40[a]	.10[b]	.15[a]	.29[a]	.22[a]	.32[a]																		
Trua	-.01	.05	.10[b]	.12[a]	.12[a]	.16[a]	.10[a]	.16[a]	.10[b]	0	.09[b]	.10[b]	.08[b]	.11[a]																	
Vand	.14[a]	.04	.22[a]	.15[a]	.20[a]	.09[b]	.16[a]	.25[a]	.15[a]	0	.27[a]	.41[a]	.09[b]	.17[a]	.33[a]																
Scho	.08[b]	.10[b]	.15[a]	.24[a]	.10[a]	.09[b]	.24[a]	.09[b]	.11[a]	.03	.12[a]	.16[a]	.15[a]	.06[c]	.08[b]	.12[a]															
Care	.10[b]	.20[a]	.21[a]	.28[a]	.14[a]	.18[a]	.22[a]	.16[a]	.09[b]	.05	.24[a]	.16[a]	.17[a]	.15[a]	.07[c]	.13[a]	.21[a]														
Bad	.08[b]	.21[a]	.25[a]	.26[a]	.17[a]	.14[a]	.25[a]	.16[a]	.08[b]	.06[c]	.17[a]	.14[a]	.16[a]	.14[a]	.06[c]	.07[c]	.44[a]	.27[a]													
Time	.07[c]	.04	.12[a]	.17[a]	.07[b]	.08[b]	.15[a]	.10[b]	.12[a]	.02	.10[a]	.12[a]	.10[a]	.03	.07[c]	.08[b]	.29[a]	.11[a]	.13[a]												
Trou	.08[b]	.19[a]	.25[a]	.32[a]	.21[a]	.22[a]	.29[a]	.19[a]	.11[a]	-.02	.26[a]	.18[a]	.22[a]	.17[a]	.11[a]	.13[a]	.29[a]	.32[a]	.35[a]	.24[a]											
Well	.07[c]	.13[a]	.15[a]	.21[a]	.12[a]	.13[a]	.22[a]	.12[a]	.13[a]	.08[b]	.16[a]	.15[a]	.14[a]	.08[b]	.11[a]	.12[a]	.41[a]	.19[a]	.25[a]	.24[a]	.32[a]										
Unca	.07[b]	.18[a]	.11[a]	.22[a]	.13[a]	.11[a]	.19[a]	.15[a]	.01	-.01	.17[a]	.10[b]	.11[a]	.16[a]	.09[b]	.11[a]	.20[a]	.45[a]	.23[a]	.18[a]	.33[a]	.23[a]									
Wron	.07[c]	.19[a]	.19[a]	.25[a]	.09[b]	.11[a]	.26[a]	.14[a]	.07[c]	.02	.17[a]	.11[a]	.12[a]	.06[c]	.06	.08[b]	.36[a]	.22[a]	.56[a]	.17[a]	.27[a]	.20[a]	.22[a]								
Best	.03	.11[a]	.14[a]	.26[a]	.12[a]	.09[b]	.24[a]	.08[b]	.05	.02	.15[a]	.11[a]	.15[a]	.08[b]	.10[b]	.10[b]	.66[a]	.19[a]	.42[a]	.29[a]	.23[a]	.43[a]	.20[a]	.40[a]							
Apol	.08[b]	.20[a]	.16[a]	.19[a]	.13[a]	.09[b]	.18[a]	.15[a]	.10[b]	.05	.12[a]	.10[b]	.12[a]	.13[a]	.04	.06[c]	.36[a]	.27[a]	.70[a]	.14[a]	.24[a]	.22[a]	.25[a]	.56[a]	.39[a]						
Remo	.06[c]	.12[a]	.17[a]	.21[a]	.16[a]	.14[a]	.18[a]	.19[a]	.09[b]	-.02	.16[a]	.09[b]	.09[b]	.15[a]	.04	.08[b]	.15[a]	.21[a]	.25[a]	.11[a]	.31[a]	.18[a]	.21[a]	.16[a]	.14[a]	.18[a]					
Effo	.11[a]	.15[a]	.16[a]	.24[a]	.12[a]	.11[a]	.27[a]	.11[a]	.10[a]	.05	.14[a]	.14[a]	.12[a]	.10[b]	.10[b]	.09[b]	.39[a]	.19[a]	.23[a]	.32[a]	.25[a]	.49[a]	.21[a]	.27[a]	.51[a]	.23[a]	.17[a]				
Unim	.07[c]	.08[b]	.12[a]	.10[a]	.08[b]	.11[a]	.08[b]	.07[b]	.04	-.02	.07[b]	.08[b]	.04	.07[c]	.02	.11[a]	.10[b]	.19[a]	.10[b]	.11[a]	.20[a]	.13[a]	.31[a]	.08[b]	.07[c]	.10[b]	.17[a]	.11[a]			
Work	.06[c]	.13[a]	.15[a]	.27[a]	.10[a]	.12[a]	.25[a]	.08[b]	.05	.01	.12[a]	.12[a]	.13[a]	.06[c]	.09[b]	.09[b]	.68[a]	.19[a]	.43[a]	.29[a]	.25[a]	.42[a]	.21[a]	.42[a]	.85[a]	.38[a]	.15[a]	.50[a]	.10[a]		
Good	.05	.17[a]	.13[a]	.14[a]	.14[a]	.11[a]	.13[a]	.11[a]	.05	.06	.06[c]	.05	.11[a]	.08[b]	.05	.01	.32[a]	.17[a]	.52[a]	.11[a]	.19[a]	.20[a]	.22[a]	.41[a]	.37[a]	.54[a]	.14[a]	.18[a]	.09[b]	.38[a]	

Note. [a]p <.001; [b]p < .01; [c]p < .05. There was a significant positive Pearson correlation between the total CBCL conduct problems score and the total ICU score, r = .60, p < .001.

Table S15

Spearman Correlations Sample 2-CU Full: ABCD-SD baseline, US

	Anim	Pers	Dest	Brea	Figh	Hang	Lyin	Phys	Runs	Fire	Steh	Steo	Swa	Thrp	Trua	Vand	Lack	cons	Help	Offe
Anim																				
Pers	.28[a]																			
Dest	.29[a]	.35[a]																		
Brea	.18[a]	.33[a]	.41[a]																	
Figh	.17[a]	.40[a]	.35[a]	.33[a]																
Hang	.14[a]	.30[a]	.23[a]	.32[a]	.42[a]															
Lyin	.15[a]	.31[a]	.32[a]	.52[a]	.26[a]	.28[a]														
Phys	.16[a]	.35[a]	.38[a]	.33[a]	.38[a]	.26[a]	.25[a]													
Runs	.03	.21[a]	.30[a]	.20[a]	.32[a]	.16[a]	.15[a]	.33[a]												
Fire	.10[a]	.14[a]	.16[a]	.15[a]	.22[a]	.16[a]	.13[a]	.14[a]	.21[a]											
Steh	.13[a]	.24[a]	.41[a]	.33[a]	.27[a]	.23[a]	.39[a]	.29[a]	.24[a]	.20[a]										
Steo	.17[a]	.15[a]	.34[a]	.26[a]	.23[a]	.19[a]	.27[a]	.28[a]	.33[a]	.30[a]	.55[a]									
Swea	.16[a]	.30[a]	.36[a]	.32[a]	.27[a]	.18[a]	.28[a]	.29[a]	.21[a]	.15[a]	.26[a]	.19[a]								
Thrp	.19[a]	.42[a]	.41[a]	.36[a]	.50[a]	.29[a]	.28[a]	.49[a]	.27[a]	.20[a]	.31[a]	.31[a]	.36[a]							
Trua	-.01	.09[a]	.13[a]	.15[a]	.13[a]	.08[a]	.10[a]	.11[a]	.25[a]	.08[a]	.12[a]	.12[a]	.10[a]	.18[a]						
Vand	.07[b]	.25[a]	.33[a]	.22[a]	.39[a]	.18[a]	.18[a]	.30[a]	.49[a]	.21[a]	.28[a]	.34[a]	.26[a]	.36[a]	.34[a]					
Lack	.17[a]	.34[a]	.39[a]	.54[a]	.34[a]	.30[a]	.41[a]	.25[a]	.20[a]	.13[a]	.31[a]	.25[a]	.30[a]	.33[a]	.10[a]	.25[a]				
cons	.10[a]	.32[a]	.28[a]	.31[a]	.20[a]	.20[a]	.28[a]	.20[a]	.12[a]	.09[a]	.17[a]	.13[a]	.19[a]	.24[a]	.06[b]	.16[a]	.38[a]			
Help	.10[a]	.21[a]	.15[a]	.19[a]	.14[a]	.10[a]	.18[a]	.13[a]	.09[a]	.08[a]	.08[a]	.06[b]	.13[a]	.17[a]	.05[c]	.13[a]	.24[a]	.62[a]		
Offe	.07[a]	.18[a]	.15[a]	.19[a]	.11[a]	.09[a]	.19[a]	.12[a]	.08[a]	.09[a]	.09[a]	.06[b]	.13[a]	.15[a]	.06[b]	.11[a]	.26[a]	.56[a]	.62[a]	

Note. $^{a}p < .001$; $^{b}p < .01$; $^{c}p < .05$. There was a significant positive Pearson correlation between the total CBCL conduct problems score and the total CU score, $r = .47$, $p < .001$.

Table S16

Spearman Correlations Sample 2-CU Boys: ABCD-SD baseline, US

	Anim	Pers	Dest	Brea	Figh	Hang	Lyin	Phys	Runs	Fire	Steh	Steo	Swa	Thrp	Trua	Vand	Lack	cons	Help	Offe
Anim																				
Pers	.33^{a}																			
Dest	.27^{a}	.37^{a}																		
Brea	.15^{a}	.31^{a}	.39^{a}																	
Figh	.14^{a}	.40^{a}	.34^{a}	.31^{a}																
Hang	.06^{c}	.25^{a}	.19^{a}	.28^{a}	.33^{a}															
Lyin	.12^{a}	.30^{a}	.27^{a}	.50^{a}	.26^{a}	.26^{a}														
Phys	.15^{a}	.37^{a}	.38^{a}	.30^{a}	.35^{a}	.23^{a}	.26^{a}													
Runs	-.01	.30^{a}	.30^{a}	.20^{a}	.41^{a}	.16^{a}	.15^{a}	.29^{a}												
Fire	.04	.12^{a}	.18^{a}	.17^{a}	.23^{a}	.15^{a}	.15^{a}	.18^{a}	.18^{a}											
Steh	.08^{b}	.25^{a}	.39^{a}	.30^{a}	.29^{a}	.19^{a}	.35^{a}	.27^{a}	.21^{a}	.28^{a}										
Steo	.07^{b}	.19^{a}	.30^{a}	.26^{a}	.27^{a}	.19^{a}	.26^{a}	.27^{a}	.31^{a}	.43^{a}	.55^{a}									
Swea	.17^{a}	.34^{a}	.37^{a}	.33^{a}	.29^{a}	.17^{a}	.30^{a}	.28^{a}	.26^{a}	.16^{a}	.29^{a}	.17^{a}								
Thrp	.18^{a}	.47^{a}	.45^{a}	.37^{a}	.48^{a}	.27^{a}	.30^{a}	.53^{a}	.36^{a}	.22^{a}	.35^{a}	.40^{a}	.37^{a}							
Trua	-.01	.11^{a}	.14^{a}	.16^{a}	.13^{a}	.02	.10^{a}	.09^{b}	.35^{a}	.11^{a}	.13^{a}	.14^{a}	.12^{a}	.22^{a}						
Vand	.04	.33^{a}	.37^{a}	.25^{a}	.48^{a}	.23^{a}	.19^{a}	.32^{a}	.65^{a}	.26^{a}	.32^{a}	.35^{a}	.31^{a}	.41^{a}	.36^{a}					
Lack	.11^{a}	.35^{a}	.39^{a}	.52^{a}	.31^{a}	.26^{a}	.39^{a}	.22^{a}	.24^{a}	.13^{a}	.32^{a}	.26^{a}	.28^{a}	.32^{a}	.12^{a}	.28^{a}				
cons	.10^{a}	.31^{a}	.30^{a}	.34^{a}	.20^{a}	.22^{a}	.30^{a}	.21^{a}	.15^{a}	.11^{a}	.19^{a}	.17^{a}	.21^{a}	.28^{a}	.07^{b}	.20^{a}	.41^{a}			
Help	.14^{a}	.24^{a}	.19^{a}	.24^{a}	.18^{a}	.13^{a}	.21^{a}	.17^{a}	.11^{a}	.09^{b}	.13^{a}	.11^{a}	.17^{a}	.23^{a}	.07^{b}	.18^{a}	.27^{a}	.61^{a}		
Offe	.07^{c}	.20^{a}	.17^{a}	.22^{a}	.12^{a}	.10^{b}	.21^{a}	.14^{a}	.08^{b}	.08^{b}	.15^{a}	.09^{b}	.15^{a}	.19^{a}	.06^{c}	.13^{a}	.28^{a}	.56^{a}	.63^{a}	

Note. ^{a}p <.001; ^{b}p < .01; ^{c}p < .05. There was a significant positive Pearson correlation between the total CBCL conduct problems score and the total CU score, r = .49, p < .001.

Table S17

Spearman Correlations Sample 2-CU Girls: ABCD-SD baseline, US

	Anim	Pers	Dest	Brea	Figh	Hang	Lyin	Phys	Runs	Fire	Steh	Steo	Swa	Thrp	Trua	Vand	Lack	cons	Help	Offe
Anim																				
Pers	.20^{a}																			
Dest	.32^{a}	.31^{a}																		
Brea	.22^{a}	.36^{a}	.43^{a}																	
Figh	.22^{a}	.40^{a}	.36^{a}	.36^{a}																
Hang	.26^{a}	.38^{a}	.30^{a}	.38^{a}	.55^{a}															
Lyin	.18^{a}	.32^{a}	.39^{a}	.55^{a}	.27^{a}	.32^{a}														
Phys	.19^{a}	.31^{a}	.39^{a}	.36^{a}	.45^{a}	.32^{a}	.23^{a}													
Runs	.08^{b}	.09^{b}	.32^{a}	.21^{a}	.20^{a}	.15^{a}	.14^{a}	.43^{a}												
Fire	.26^{a}	.19^{a}	.10^{b}	.11^{a}	.24^{a}	.18^{a}	.09^{b}	-.01	.28^{a}											
Steh	.18^{a}	.23^{a}	.45^{a}	.39^{a}	.26^{a}	.27^{a}	.44^{a}	.35^{a}	.28^{a}	.10^{b}										
Steo	.28^{a}	.12^{a}	.41^{a}	.28^{a}	.19^{a}	.19^{a}	.29^{a}	.33^{a}	.36^{a}	.15^{a}	.56^{a}									
Swea	.15^{a}	.22^{a}	.34^{a}	.28^{a}	.25^{a}	.21^{a}	.23^{a}	.31^{a}	.14^{a}	.08^{b}	.24^{a}	.24^{a}								
Thrp	.20^{a}	.32^{a}	.31^{a}	.34^{a}	.57^{a}	.33^{a}	.24^{a}	.39^{a}	.10^{a}	.16^{a}	.26^{a}	.22^{a}	.33							
Trua	-.01	.05	.10^{b}	.12^{a}	.12^{a}	.16^{a}	.10^{b}	.16^{a}	.10^{b}	0	.10^{b}	.10^{b}	.08^{b}	.11^{a}						
Vand	.14^{a}	.04	.22^{a}	.15^{a}	.20^{a}	.09^{b}	.16^{a}	.25^{a}	.15^{a}	0	.28^{a}	.41^{a}	.09^{b}	.17^{a}	.33					
Lack	.28^{a}	.32^{a}	.38^{a}	.55^{a}	.38^{a}	.36^{a}	.44^{a}	.31^{a}	.15^{a}	.12^{a}	.31^{a}	.25^{a}	.33^{a}	.35^{a}	.07^{c}	.17^{a}				
cons	.11^{a}	.32^{a}	.25^{a}	.26^{a}	.20^{a}	.17^{a}	.24^{a}	.16^{a}	.08^{b}	.03	.16^{a}	.11^{a}	.15^{a}	.16^{a}	.03	.06^{c}	.34^{a}			
Help	.04	.15^{a}	.08^{b}	.08^{b}	.08^{b}	.06^{c}	.13^{a}	.04	.06^{c}	.05	.03	.01	.04	.05	.01	.01	.18^{a}	.61^{a}		
Offe	.08^{b}	.14^{a}	.09^{b}	.14^{a}	.09^{b}	.09^{b}	.15^{a}	.07^{c}	.07^{c}	.08^{b}	.02	.04	.07	.06	.07^{c}	.06	.21^{a}	.56^{a}	.60^{a}	

Note. ^{a}p <.001; ^{b}p < .01; ^{c}p < .05. There was a significant positive Pearson correlation between the total CBCL conduct problems score and the total CU score, r = .40, p < .001.

Table S18

Spearman Correlations Sample 3a Full: ELISA wave 6, Spain

	Symp	Shar	Done	Indi	Upse	Remo	Unca	Capa	Guil	Expr	Teas	Viol	Thre	Hitw	Hitp	Take	Brok	Inte
Symp																		
Shar	.54[a]																	
Done	.36[a]	.37[a]																
Indi	.50[a]	.58[a]	.40[a]															
Upse	.39[a]	.43[a]	.38[a]	.49[a]														
Remo	.33[a]	.36[a]	.46[a]	.44[a]	.41[a]													
Unca	.47[a]	.49[a]	.39[a]	.58[a]	.43[a]	.44[a]												
Capa	.43[a]	.45[a]	.45[a]	.53[a]	.44[a]	.51[a]	.50[a]											
Guil	.38[a]	.42[a]	.49[a]	.43[a]	.37[a]	.54[a]	.44[a]	.57[a]										
Expr	.42[a]	.42[a]	.42[a]	.50[a]	.41[a]	.48[a]	.46[a]	.64[a]	.53[a]									
Teas	.23[a]	.21[a]	.22[a]	.27[a]	.33[a]	.29[a]	.34[a]	.29[a]	.25[a]	.26[a]								
Viol	.22[a]	.22[a]	.27[a]	.28[a]	.26[a]	.35[a]	.28[a]	.34[a]	.33[a]	.29[a]	.39[a]							
Thre	.14[a]	.16[a]	.14[a]	.19[a]	.28[a]	.23[a]	.24[a]	.24[a]	.20[a]	.22[a]	.44[a]	.46[a]						
Hitw	.16[a]	.22[a]	.17[a]	.22[a]	.30[a]	.27[a]	.20[a]	.31[a]	.23[a]	.28[a]	.35[a]	.38[a]	.42[a]					
Hitp	.15[a]	.19[a]	.16[a]	.20[a]	.22[a]	.24[a]	.22[a]	.24[a]	.20[a]	.24[a]	.38[a]	.36[a]	.39[a]	.45[a]				
Take	.18[a]	.17[a]	.20[a]	.22[aa]	.26[a]	.29[a]	.22[a]	.24[a]	.24[a]	.25[a]	.35[a]	.40[a]	.32[a]	.31[a]	.27[a]			
Brok	.13[a]	.15[a]	.16[a]	.14	.28[a]	.25[a]	.22[a]	.26[a]	.24[a]	.25[a]	.28[a]	.32[a]	.33[a]	.45[a]	.33[a]	.37[a]		
Inte	.15[a]	.14[a]	.15[a]	.21[a]	.23[a]	.20[a]	.22[a]	.23[a]	.22[a]	.23[a]	.36[a]	.36[a]	.37[a]	.38[a]	.36[a]	.40[a]	.41[a]	

Note. [a]$p < .001$. There was a significant positive Pearson correlation between the total CP score and the CU subscale of the CPTI, $r = .51$, $p < .001$.

Table S19

Spearman Correlations Sample 3a Boys: ELISA wave 6, Spain

	Symp	Shar	Done	Indi	Upse	Remo	Unca	Capa	Guil	Expr	Teas	Viol	Thre	Hitw	Hitp	Take	Brok	Inte
Symp																		
Shar	.56[a]																	
Done	.36[a]	.32[a]																
Indi	.54[a]	.57[a]	.39[a]															
Upse	.45[a]	.43[a]	.40[a]	.52[a]														
Remo	.37[a]	.38[a]	.42[a]	.46[a]	.44[a]													
Unca	.51[a]	.52[a]	.39[a]	.61[a]	.46[a]	.44[a]												
Capa	.44[a]	.45[a]	.43[a]	.55[a]	.45[a]	.48[a]	.49[a]											
Guil	.36[a]	.43[a]	.49[a]	.40[a]	.38[a]	.52[aa]	.40[a]	.54[a]										
Expr	.45[a]	.42[a]	.43[a]	.49[a]	.43[a]	.50[a]	.47[a]	.63[a]	.53[a]									
Teas	.24[a]	.20[a]	.23[a]	.28[a]	.35[a]	.31[a]	.33[a]	.29[a]	.24[a]	.28[a]								
Viol	.25[a]	.20[a]	.26[a]	.30[a]	.30[a]	.38[a]	.29[a]	.34[a]	.32[a]	.32[a]	.42[a]							
Thre	.18[a]	.15[a]	.15[a]	.21[a]	.33[a]	.26[a]	.25[a]	.24[a]	.20[a]	.27[a]	.45[a]	.48[a]						
Hitw	.21[a]	.21[a]	.16[a]	.19[a]	.32[a]	.26[a]	.23[a]	.29[a]	.22[a]	.30[a]	.39[a]	.40[a]	.49[a]					
Hitp	.16[a]	.14[a]	.13[b]	.18[a]	.21[a]	.24[a]	.21[a]	.18[a]	.17[a]	.24[a]	.42[a]	.37[a]	.42[a]	.43[a]				
Take	.22[a]	.16[a]	.16[a]	.22[a]	.26[a]	.30[a]	.21[a]	.23[a]	.23[a]	.25[a]	.35[a]	.43[a]	.37[a]	.36[a]	.31[a]			
Brok	.15[a]	.10[c]	.13[b]	.10[c]	.28[a]	.23[a]	.20[a]	.23[a]	.20[a]	.24[a]	.29[a]	.35[a]	.38[a]	.47[a]	.33[a]	.38[a]		
Inte	.16[a]	.11[b]	.12[b]	.18[a]	.25[a]	.21[a]	.20[a]	.20[a]	.21[a]	.22[a]	.34[a]	.38[a]	.38[a]	.42[a]	.36[a]	.41[a]	.40[a]	

Note. [a]$p < .001$; [b]$p < .01$; [c]$p < .05$. There was a significant positive Pearson correlation between the total CP score and the CU subscale of the CPTI, $r = .48$, $p < .001$.

Table S20

Spearman Correlations Sample 3a Girls: ELISA wave 6, Spain

	Symp	Shar	Done	Indi	Upse	Remo	Unca	Capa	Guil	Expr	Teas	Viol	Thre	Hitw	Hitp	Take	Brok	Inte
Symp																		
Shar	.51[a]																	
Done	.36[a]	.42[a]																
Indi	.44[a]	.60[a]	.40[a]															
Upse	.31[a]	.41[a]	.35[a]	.43[a]														
Remo	.29[a]	.32[a]	.49[a]	.41[a]	.37[a]													
Unca	.40[a]	.44[a]	.38[a]	.53[a]	.38[a]	.44[a]												
Capa	.42[a]	.44[a]	.45[a]	.50[a]	.42[a]	.53[a]	.51[a]											
Guil	.38[a]	.41[a]	.47[a]	.46[a]	.35[a]	.56[a]	.47[a]	.61[a]										
Expr	.37[a]	.42[a]	.41[a]	.51[a]	.38[a]	.45[a]	.43[a]	.65[a]	.51[a]									
Teas	.21[a]	.21[a]	.21[a]	.25[a]	.29[a]	.26[a]	.34[a]	.28[a]	.25[a]	.23[a]								
Viol	.18[a]	.23[a]	.28[a]	.25[a]	.19[a]	.31[a]	.27[a]	.34[a]	.33[a]	.26[a]	.36[a]							
Thre	.07	.16[a]	.10[b]	.16[a]	.21[a]	.20[a]	.23[a]	.22[a]	.18[a]	.14[a]	.41[a]	.42[a]						
Hitw	.09[c]	.24[a]	.17[a]	.24[a]	.26[a]	.28[a]	.15[a]	.33[a]	.25[a]	.24[a]	.31[a]	.36[a]	.33[a]					
Hitp	.11[b]	.22[a]	.17[a]	.21[a]	.22[a]	.23[a]	.22[a]	.29[a]	.22[a]	.23[a]	.31[a]	.32[a]	.34[a]	.48[a]				
Take	.12[b]	.16[a]	.23[a]	.21[a]	.26[a]	.27[a]	.22[a]	.24[a]	.24[a]	.24[a]	.34[a]	.36[a]	.26[a]	.25[a]	.22[a]			
Brok	.11[b]	.22[a]	.20[a]	.19[a]	.27[a]	.26[a]	.24[a]	.30[a]	.29[a]	.25[a]	.26[a]	.30[a]	.27[a]	.42[a]	.34[a]	.35[a]		
Inte	.12[b]	.17[a]	.16[a]	.23[a]	.18[a]	.19[a]	.25[a]	.25[a]	.21[a]	.24[a]	.37[a]	.32[a]	.35[a]	.33[a]	.34[a]	.39[a]	.44[a]	

Note. [a]$p < .001$; [b]$p < .01$. There was a significant positive Pearson correlation between the total CP score and the CU subscale of the CPTI, $r = .54$, $p < .001$.

Table S21

Spearman Correlations Sample 3b Full: ELISA wave 7, Spain

	Anim	Pers	Dest	Brea	Figh	Hang	Lyin	Phys	Runs	Fire	Steh	Steo	Swea	Thrp	Trua	Vand	Lack	Cons	Help	Offe
Anim																				
Pers	.23^{a}																			
Dest	.11^{a}	.21^{a}																		
Brea	.10^{a}	.19^{a}	.21^{a}																	
Figh	.08^{b}	.34^{a}	.18^{a}	.26^{a}																
Hang	.01	.11^{a}	.11^{a}	.14^{a}	.24^{a}															
Lyin	.07^{b}	.19^{a}	.12^{a}	.40^{a}	.14^{a}	.11^{a}														
Phys	.03	.20^{a}	.22^{a}	.20^{a}	.22^{a}	.11^{a}	.15^{a}													
Runs	.19^{a}	.13^{a}	.11^{a}	.10^{a}	.08^{b}	.04	.07^{b}	.03												
Fire	.07^{c}	.09^{b}	.10^{a}	.12^{a}	.24^{a}	.18^{a}	.06^{c}	.09^{b}	.07^{c}											
Steh	-.01	.10^{b}	.08^{b}	.09^{b}	.17^{a}	.10^{a}	.13^{a}	.17^{a}	.15^{a}	.05										
Steo	.09^{b}	-.02	.11^{a}	.09^{b}	.13^{a}	.11^{a}	.03	.18^{a}	.09^{b}	.14^{a}	.23^{a}									
Swea	.06^{c}	.19^{a}	.23^{a}	.30^{a}	.19^{a}	.14^{a}	.25^{a}	.17^{a}	.06^{c}	.12^{a}	.10^{b}	.12^{a}								
Thrp	-.02	.31^{a}	.14^{a}	.18^{a}	.23^{a}	.10^{b}	.10^{a}	.28^{a}	.04	.10^{a}	.11^{a}	.15^{a}	.23^{a}							
Trua	-.01	-.01	.09^{b}	.03	-.01	.07^{c}	.01	.05	-.01	.09^{b}	-.01	-.01	.05	-.01						
Vand	.11^{a}	.04	.08^{b}	.05	.15^{a}	.13^{a}	.02	.26^{a}	-.01	.08^{b}	-.01	.10^{a}	.14^{a}	.11^{a}	-.01					
Lack	.15^{a}	.34^{a}	.23^{a}	.27^{a}	.16^{a}	.07^{c}	.28^{a}	.19^{a}	.18^{a}	.12^{a}	.10^{a}	.09^{b}	.18^{a}	.20^{a}	-.02	.04				
Cons	.05	.27^{a}	.12^{a}	.20^{a}	.13^{a}	.06^{c}	.21^{a}	.21^{a}	.05	.11^{a}	.10^{b}	.03	.07^{b}	.10^{a}	.03	.04	.28^{a}			
Help	-.01	.08^{b}	.04	.10^{a}	-.02	-.02	.18^{a}	.09^{b}	-.01	-.03	.01	-.04	.12^{a}	-.02	-.03	-.01	.08^{b}	.42^{a}		
Offe	.03	.14^{a}	.05	.20^{a}	.08^{b}	.08^{b}	.14^{a}	.02	.02	-.02	.07^{b}	.05	.19^{a}	.02	.04	.03	.15^{a}	.33^{a}	.40^{a}	

Note. $^{a}p < .001$; $^{b}p < .01$; $^{c}p < .05$. There was a significant positive Pearson correlation between the total CBCL conduct problems score and the total CU score, $r = .43$, $p < .001$.

Table S22

Spearman Correlations Sample 3b Boys: ELISA wave 7, Spain

	Anim	Pers	Dest	Brea	Figh	Hang	Lyin	Phys	Runs	Fire	Steh	Steo	Swea	Thrp	Trua	Vand	Lack	Cons	Help	Offe
Anim																				
Pers	.21[a]																			
Dest	.17[a]	.21[a]																		
Brea	.10[c]	.16[a]	.23[a]																	
Figh	.05	.26[a]	.20[a]	.25[a]																
Hang	.02	.10[c]	.11[b]	.16[a]	.22[a]															
Lyin	.07	.21[a]	.10[c]	.41[a]	.13[b]	.11[b]														
Phys	.05	.22[a]	.21[a]	.20[a]	.21[a]	.15[a]	.16[a]													
Runs	.13[b]	.05	.10[c]	.11[b]	.05	.02	.03	.05												
Fire	.11[b]	.04	.18[a]	.14[a]	.28[a]	.14[b]	.05	.16[a]	.11[b]											
Steh	-.01	.05	.03	.08[c]	.11[b]	.06	.11[b]	.18[a]	.12[b]	.10[c]										
Steo	.13[b]	-.02	.09[c]	.11[b]	.11[b]	.11[b]	.01	.18[a]	.12[b]	.21[a]	.24[a]									
Swea	.05	.17[a]	.23[a]	.26[a]	.18[a]	.14[b]	.22[a]	.19[a]	.09[c]	.18[a]	.12[b]	.13[b]								
Thrp	-.02	.19[a]	.10[b]	.17[a]	.18[a]	.07	.06	.34[a]	-.02	.16[a]	-.02	.18[a]	.24[a]							
Trua	-.01	-.02	.15[a]	.07	-.02	.12[b]	.03	.08[c]	-.01	-.01	-.01	-.01	.08[c]	-.01						
Vand	.16[a]	.07	.05	.06	.15[a]	.16[a]	.03	.24[a]	-.01	.13[b]	-.01	.15[a]	.13[b]	.24[a]	-.01					
Lack	.16[a]	.33[a]	.20[a]	.26[a]	.13[b]	.04	.24[a]	.16[a]	.22[a]	.17[a]	.06	.11[b]	.15[a]	.03	-.03	.03				
Cons	.06	.28[a]	.11[b]	.23[a]	.13[b]	.03	.25[a]	.24[a]	.02	.12[b]	.08[c]	.04	.08	.14[a]	.01	.04	.30[a]			
Help	-.01	.10[b]	.02	.13[b]	-.01	-.01	.20[a]	.09[c]	-.01	-.03	.02	-.05	.12[b]	.02	-.04	-.04	.12[b]	.47[a]		
Offe	.04	.15[a]	.02	.19[a]	.07	.11[b]	.18[a]	-.02	.02	-.04	.05	.05	.16[a]	-.01	.04	-.01	.16[a]	.32[a]	.39[a]	

Note. $^a p < .001$; $^b p < .01$; $^c p < .05$. There was a significant positive Pearson correlation between the total CBCL conduct problems score and the total CU score, $r = .42$, $p < .001$.

Table S23

Spearman Correlations Sample 3b Girls: ELISA wave 7, Spain

	Anim	Pers	Dest	Brea	Figh	Hang	Lyin	Phys	Runs	Fire	Steh	Steo	Swea	Thrp	Trua	Vand	Lack	Cons	Help	Offe
Anim																				
Pers	.27[a]																			
Dest	-.01	.19[a]																		
Brea	.10[c]	.21[a]	.16[a]																	
Figh	.13[b]	.47[a]	.14[b]	.26[a]																
Hang	-.02	.12[b]	.10[c]	.10[c]	.25[a]															
Lyin	.08[c]	.16[a]	.13[b]	.38[a]	.15[a]	.10[c]														
Phys	-.01	.16[a]	.25[a]	.19[a]	.23[a]	.04	.13[b]													
Runs	.33[a]	.27[a]	.12[b]	.08[c]	.13[b]	.08[c]	.14[b]	-.01												
Fire	-.01	.17[a]	-.02	.09[c]	.17[a]	.23[a]	.06	-.02	-.01											
Steh	-.01	.16[a]	.14[b]	.11[b]	.25[a]	.15[a]	.14[a]	.16[a]	.20[a]	-.01										
Steo	.00	-.01	.15[a]	.04	.17[a]	.10[c]	.05	.17[a]	.00	-.01	.24[a]									
Swea	.06	.20[a]	.24[a]	.33[a]	.21[a]	.12[b]	.28[a]	.14[b]	-.02	.02	.07	.09[c]								
Thrp	-.01	.45[a]	.20[a]	.21[a]	.30[a]	.13[b]	.15[a]	.24[a]	.11[b]	.06	.21[a]	.14[a]	.26[a]							
Trua	.00	-.01	-.01	-.03	-.01	-.01	-.03	-.01	.00	.27[a]	-.01	.00	-.02	-.01						
Vand	.00	-.01	.12[b]	.03	.13[b]	.08[c]	.02	.28[a]	.00	-.01	-.01	.00	.15[a]	-.01	.00					
Lack	.13[b]	.36[a]	.28[a]	.28[a]	.21[a]	.11[b]	.33[a]	.24[a]	.13[b]	.06	.15[a]	.07	.22[a]	.33[a]	-.02	.05				
Cons	.04	.25[a]	.11[b]	.14[a]	.11[a]	.09[c]	.15[a]	.14[a]	.11[b]	.09[c]	.12[b]	-.02	.05	.07	.06	.04	.26[a]			
Help	-.03	.03	.06	.06	-.06	-.06	.14[b]	.06	-.03	-.04	.00	-.02	.12[b]	-.05	-.02	.04	.04	.33[a]		
Offe	.00	.12[b]	.07	.18[a]	.08[c]	.04	.08[c]	.07	.00	.00	.10[c]	.02	.22[a]	.05	.04	.07	.15[a]	.34[a]	.39[a]	

Note. [a]p <.001; [b]p < .01; [c]p < .05. There was a significant positive Pearson correlation between the total CBCL conduct problems score and the total CU score, r = .42, p < .001.

5. Adjacency Matrix of the Estimated Psychological Network (EBICglasso, Spearman)

Table S24

Adjacency matrix of the estimated network for Sample 1 Full: ABCD baseline, US

	Anim	Pers	Dest	Brea	Figh	Hang	Lyin	Phys	Runs	Fire	Steh	Steo	Swea	Thrp	Trua	Vand	Lack	Cons	Help	Offe
Anim																				
Pers	.08																			
Dest	.07	.07																		
Brea	0	.05	.12																	
Figh	.02	.06	.07	.05																
Hang	0	.08	0	.09	.12															
Lyin	0	.05	.05	.29	.01	.08														
Phys	.01	.13	.14	.06	.17	0	0													
Runs	.07	0	0	.01	.04	0	0	.03												
Fire	.05	.01	0	0	0	.02	.01	0	.01											
Steh	.04	.01	.10	.06	0	0	.13	0	.03	.05										
Steo	.05	0	.04	0	.07	.03	.04	0	.04	.08	.36									
Swea	.02	.05	.07	.09	.04	.08	.02	.03	.02	.05	.02	.02								
Thrp	.02	.16	.08	0	.12	0	0	.23	.06	0	.05	.01	.11							
Trua	0	0	.02	0	0	0	0	.03	.10	0	0	0	.03	.03						
Vand	.05	.03	.09	0	.05	.05	0	0	.04	.04	.10	.09	.03	.07	.04					
Lack	0	.11	.08	.19	.03	.02	.11	.03	.01	.02	.02	0	.02	.02	0	.03				
Cons	0	.09	.04	.06	.02	0	.05	.02	0	0	0	0	.03	0	0	0	.11			
Help	.02	.01	0	0	0	0	0	0	0	0	0	0	0	.02	0	0	.03	.35		
Offe	0	0	0	0	0	0	0	0	0	0	0	0	0	0	0	0	.01	.26	.38	

Note. It is a symmetric matrix where each entry represents the strength of the connection between two nodes (variables). Global network strength (i.e., the sum of absolute edge values for each network) = 7.68.

Table S25

Adjacency matrix of the estimated network for Sample 1 Boys: ABCD baseline, US

	Anim	Pers	Dest	Brea	Figh	Hang	Lyin	Phys	Runs	Fire	Steh	Steo	Swea	Thrp	Trua	Vand	Lack	Cons	Help	Offe
Anim																				
Pers	.07																			
Dest	.09	.05																		
Brea	0	.05	.13																	
Figh	.02	.05	.07	.05																
Hang	0	.07	0	.10	.11															
Lyin	0	.05	.03	.28	.01	.09														
Phys	.01	.14	.15	.04	.16	0	.02													
Runs	.09	0	0	.01	.04	0	0	.03												
Fire	.04	0	0	0	0	.03	.02	.01												
Steh	.06	.01	.09	.04	0	0	.12	0	.04	.04										
Steo	.04	0	.03	0	.07	.05	.05	0	.05	.09	.38									
Swea	0	.03	.09	.09	.04	.07	.03	.03	.03	.02	.04	0								
Thrp	.01	.18	.08	.01	.10	0	0	.22	.07	.01	.06	.01	.11							
Trua	0	0	0	0	.01	0	0	.03	.12	.01	0	0	.03	0						
Vand	.02	.04	.11	0	.05	.05	0	0	0	.06	.11	.09	.03	.04	.09					
Lack	0	.10	.07	.19	.03	.01	.11	.02	.02	.02	.02	0	.03	.02	0	.04				
Cons	0	.08	.03	.06	.03	0	.03	.02	0	0	0	0	.03	.02	0	0	.11			
Help	.02	.02	0	0	0	0	0	0	0	0	0	0	0	.02	0	.01	.03	.38		
Offe	0	0	0	0	0	0	0	0	0	0	0	0	0	.01	0	0	.01	.24	.37	

Note. It is a symmetric matrix where each entry represents the strength of the connection between two nodes (variables). Global network strength (i.e., the sum of absolute edge values for each network) = 7.69.

Table S26

Adjacency matrix of the estimated network for Sample 1 Girls: ABCD baseline, US

	Anim	Pers	Dest	Brea	Figh	Hang	Lyin	Phys	Runs	Fire	Steh	Steo	Swea	Thrp	Trua	Vand	Lack	Cons	Help	Offe
Anim																				
Pers	.08																			
Dest	.02	.09																		
Brea	0	.06	.10																	
Figh	0	.06	.07	.04																
Hang	0	.09	.02	.06	.11															
Lyin	0	.04	.07	.31	0	.05														
Phys	.01	.11	.12	.07	.17	0	0													
Runs	.02	.01	.01	.02	.01	0	0	.01												
Fire	.06	0	0	0	.06	0	0	-.02	.01											
Steh	.03	0	.12	.09	.01	0	.13	0	0	.07										
Steo	.06	0	.07	0	.06	0	.04	0	.01	.05	.32									
Swea	.07	.08	.02	.07	.05	.07	.01	0	0	.07	.01	.07								
Thrp	.04	.12	.08	0	.14	0	0	.23	.04	0	.04	0	.09							
Trua	-.01	0	.04	0	0	0	0	.01	.08	0	0	0	.03	.05						
Vand	.11	0	.06	0	.03	.03	0	.03	.10	0	.09	.10	0	.12	0					
Lack	0	.11	.09	.19	.03	.05	.11	.06	0	.01	.01	.01	.02	.03	.02	0				
Cons	0	.10	.04	.05	0	.01	.08	.01	0	0	0	.01	.03	0	0	0	.11			
Help	0	0	0	0	0	0	0	0	0	0	0	0	0	.01	0	0	.03	.30		
Offe	0	0	0	0	0	-.02	0	0	.02	0	0	0	0	0	0	0	.01	.27	.38	

Note. It is a symmetric matrix where each entry represents the strength of the connection between two nodes (variables). Global network strength (i.e., the sum of absolute edge values for each network) = 7.45.

Table S27

Adjacency matrix of the estimated network for Sample 2-ICU Full: ABCD-SD baseline, US

	Anim	Pers	Dest	Brea	Figh	Hang	Lyin	Phys	Runs	Fire	Steh	Steo	Swa	Thrp	Trua	Vand	Scho	Care	Bad	Time	Trou	Well	Unca	Wrong	Best	Apol	Remo	Effo	Unim	Work	Good
Anim																															
Pers	.11																														
Dest	.10	.03																													
Brea	.02	.05	.14																												
Figh	.01	.11	0	.04																											
Hang	.02	.07	0	.10	.18																										
Lyin	0	.07	.03	.29	0	.07																									
Phys	.02	.13	.09	0	.09	.04	0																								
Runs	-.02	0	.03	0	.09	0	0	.09																							
Fire	0	0	0	0	.01	.01	0	0	.03																						
Steh	0	.01	.19	.02	.02	.02	.15	.05	.03	.04																					
Steo	.07	0	.04	.03	0	0	.01	.01	.07	.17	.32																				
Swea	.04	.06	.13	.06	.01	.01	.06	.02	.01	.03	.01	0																			
Thrp	0	.07	.11	.05	.22	0	0	.27	0	.09	0	.05	.14																		
Trua	-.01	0	0	0	0	0	0	0	.11	0	0	0	0	0																	
Vand	0	0	.07	0	.12	0	0	.06	.34	0	0	.13	.02	.03	.20																
Scho	0	0	0	.03	0	0	0	0	0	0	0	0	.01	0	0	0															
Care	.01	.08	.06	.02	0	0	.04	.01	.01	0	0	0	0	.03	.02	0	0														
Bad	0	0	0	.01	0	0	0	0	0	0	0	0	0	0	0	0	.10	.03													
Time	0	0	0	0	0	0	.01	0	.01	0	.02	.01	.01	0	.02	0	.07	0	0												
Trou	0	0	.05	.08	.02	.04	.02	0	0	0	.01	0	.03	0	.01	0	0	.09	.10	.07											
Well	0	0	0	.01	0	0	.02	0	0	.02	0	0	.01	0	.02	0	.15	.01	0	.06	.12										
Unca	0	.06	0	.01	0	0	0	0	0	0	0	0	0	.01	0	0	0	.24	0	.03	.07	.04									
Wron	0	0	0	.04	0	0	.06	0	0	0	0	0	0	0	0	0	0	.04	.16	.01	.04	0	0								
Best	0	0	0	0	0	0	0	0	0	0	0	0	.01	0	0	0	.21	0	0	0	0	.07	0	.01							
Apol	0	.02	0	0	0	0	0	0	0	0	0	0	0	0	0	0	0	.04	.42	0	0	0	.02	.21	.03						
Remo	0	0	0	.01	0	.01	0	.02	0	0	0	0	0	0	0	0	0	.10	.11	.01	.24	.03	.10	.06	0	0					
Effo	0	0	0	0	0	0	.06	0	0	0	0	0	0	0	0	0	.02	0	-.01	.14	0	.28	.01	.04	.14	0	.02				
Unim	0	0	.02	0	0	.01	0	0	0	0	0	0	0	.02	0	.02	0	.13	0	0	.04	.04	.28	0	0	0	.09	.01			
Work	0	0	0	.02	0	0	.04	0	0	0	0	0	0	0	0	0	.24	0	.01	.04	0	.01	0	.06	.59	0	0	.13	0		
Good	0	.03	0	0	0	0	0	.01	0	0	0	0	0	0	0	0	.01	0	.14	0	0	0	.04	.08	.06	.24	0	0	.05	.05	

Note. It is a symmetric matrix where each entry represents the strength of the connection between two nodes (variables). Global network strength (i.e., the sum of absolute edge values for each network) = 13.19.

Table S28

Adjacency matrix of the estimated network for Sample 2-ICU Boys: ABCD-SD baseline, US

	Anim	Pers	Dest	Brea	Figh	Hang	Lyin	Phys	Runs	Fire	Steh	Steo	Swa	Thrp	Trua	Vand	Scho	Care	Bad	Time	Trou	Well	Unca	Wrong	Best	Apol	Remo	Effo	Unim	Work	Good
Anim																															
Pers	.17																														
Dest	.07	.04																													
Brea	.02	.03	.15																												
Figh	0	.13	0	.04																											
Hang	0	.04	0	.09	.14																										
Lyin	0	.06	0	.27	0	.09																									
Phys	.01	.14	.09	0	.05	.03	.01																								
Runs	-.04	.02	0	0	.08	0	0	.03																							
Fire	0	0	0	0	.01	.01	0	.03	0																						
Steh	0	0	.15	.02	.02	0	.11	.04	0	.09																					
Steo	0	0	0	.01	0	0	.02	.02	.08	.22	.31																				
Swea	.04	.10	.10	.08	.01	0	.10	0	.02	.02	.04	0																			
Thrp	.01	.07	.13	.04	.17	.04	0	.32	.02	.07	0	.12	.13																		
Trua	0	0	.01	0	0	-.02	0	0	.18	0	.03	0	0	0																	
Vand	0	.02	.08	0	.14	0	0	.05	.47	0	.05	.08	.03	.03	.12																
Scho	-.01	0	0	.04	0	0	0	0	0	0	0	0	0	0	0	0															
Care	0	.11	.06	0	0	0	.04	.01	.01	0	0	0	0	.04	.04	0	0														
Bad	0	0	0	.01	0	0	0	.01	0	0	0	0	0	0	0	0	.07	.06													
Time	0	0	0	0	0	0	.01	0	.01	0	.05	0	.01	0	.02	0	.08	0	0												
Trou	0	0	.06	.09	.01	.04	0	0	0	.02	0	0	.02	.02	.02	0	0	.09	.11	.06											
Well	0	0	0	.01	0	.01	.01	0	0	.01	0	0	0	0	0	0	.18	.01	0	.08	.13										
Unca	.01	.06	0	.01	0	.01	0	0	0	0	0	0	0	0	0	0	0	.23	0	.01	.07	.02									
Wron	0	0	0	.04	0	0	.07	0	0	0	0	0	0	0	0	0	0	.05	.14	0	.05	0	0								
Best	0	0	0	0	0	0	0	0	0	0	0	0	0	0	0	0	.22	0	0	0	0	.07	0	.02							
Apol	0	.01	0	0	0	0	0	0	0	0	0	0	0	0	0	0	0	.01	.41	0	0	0	.04	.19	.03						
Remo	0	0	0	0	0	.01	0	0	0	0	0	0	0	0	0	0	0	.12	.12	.04	.26	.03	.09	.07	0	0					
Effo	0	0	0	0	0	0	.04	0	0	0	0	0	0	0	0	0	.04	0	-.01	.12	0	.29	.03	.06	.14	0	.03				
Unim	0	0	.02	0	0	0	0	0	0	0	0	0	0	.04	0	.01	0	.17	0	0	.01	.05	.24	0	0	0	.08	.01			
Work	0	0	0	.01	0	0	.07	0	0	0	0	0	.01	0	0	0	.22	0	.01	.05	0	0	0	.05	.58	.03	0	.13	0		
Good	.01	.04	0	.02	0	0	0	.01	0	0	0	0	0	0	0	0	.03	0	.13	0	.03	0	.03	.11	.06	.25	0	0	.05	.03	

Note. It is a symmetric matrix where each entry represents the strength of the connection between two nodes (variables). Global network strength (i.e., the sum of absolute edge values for each network) = 13.45.

Table S29

Adjacency matrix of the estimated network for Sample 2-ICU Girls: ABCD-SD baseline, US

	Anim	Pers	Dest	Brea	Figh	Hang	Lyin	Phys	Runs	Fire	Steh	Steo	Swa	Thrp	Trua	Vand	Scho	Care	Bad	Time	Trou	Well	Unca	Wrong	Best	Apol	Remo	Effo	Unim	Work	Good
Anim																															
Pers	.01																														
Dest	.13	.02																													
Brea	.01	.08	.10																												
Figh	0	.08	.02	.03																											
Hang	.10	.11	0	.12	.22																										
Lyin	0	.06	.09	.31	0	.03																									
Phys	.03	.10	.07	.01	.21	.04	0																								
Runs	0	0	.06	0	.05	0	0	.19																							
Fire	.12	0	0	0	.01	0	0	-.06	.20																						
Steh	0	.02	.22	.03	0	.02	.18	.06	.09	0																					
Steo	.15	0	.10	.03	0	0	0	0	.05	.03	.28																				
Swea	.02	.01	.17	.04	0	.02	0	.04	0	0	0	.04																			
Thrp	0	.04	.07	.04	.31	0	0	.13	0	.04	.03	0	.14																		
Trua	-.01	0	0	0	.02	.04	0	.04	.01	0	0	0	0	0																	
Vand	0	-.01	.04	0	.08	0	0	.09	.02	0	0	.19	0	0	.25																
Scho	0	0	0	.01	0	0	.01	0	0	0	0	0	.01	0	0	0															
Care	.01	.04	.03	.06	0	0	.02	0	0	0	.03	0	0	0	0	.01	0														
Bad	0	0	.02	0	0	0	.01	0	0	0	0	0	0	0	0	0	.13	0													
Time	0	0	0	.02	0	0	0	0	0	0	0	.01	0	0	0	0	.06	0	0												
Trou	0	0	.01	.05	.03	.02	.05	0	0	0	.02	0	.03	0	0	0	0	.09	.08	.07											
Well	0	0	0	0	0	0	.02	0	0	0	0	.01	.03	0	.02	.01	.12	0	0	.04	.11										
Unca	0	.06	0	.01	0	0	0	.02	-.01	0	0	0	0	.02	.01	.01	0	.26	0	.03	.08	.07									
Wron	0	.03	0	.03	0	0	.04	0	0	0	0	0	0	0	0	0	.01	.03	.18	0	.03	0	.01								
Best	0	0	0	0	0	0	0	0	0	0	0	0	.01	0	0	0	.19	0	.01	.01	0	.08	0	.01							
Apol	0	.02	0	0	0	0	0	0	0	0	0	0	0	0	0	0	0	.06	.42	0	0	0	.01	.23	.02						
Remo	0	0	0	.01	0	.01	.01	.04	0	0	0	0	0	0	0	0	0	.07	.10	0	.21	.03	.10	.04	0	0					
Effo	0	0	0	0	0	0	.07	0	0	0	0	0	0	0	.01	0	0	.02	0	.15	0	.26	0	.03	.12	0	.01				
Unim	0	0	.02	0	0	.02	0	0	0	0	0	0	0	0	0	.02	0	.07	0	.02	.08	.02	.32	0	0	.01	.10	0			
Work	0	0	0	.03	0	0	0	0	0	0	0	0	0	0	0	0	.25	0	.01	.04	0	.03	0	.08	.60	0	0	.12	0		
Good	0	.01	0	0	0	0	0	0	0	0	0	0	0	0	0	0	0	0	.16	0	0	0	.04	.05	.05	.24	0	0	.03	.05	

Note. It is a symmetric matrix where each entry represents the strength of the connection between two nodes (variables). Global network strength (i.e., the sum of absolute edge values for each network) = 12.93.

Table S30

Adjacency matrix of the estimated network for Sample 2-CU Full: ABCD-SD baseline, US

	Anim	Pers	Dest	Brea	Figh	Hang	Lyin	Phys	Runs	Fire	Steh	Steo	Swa	Thrp	Trua	Vand	Lack	cons	Help	Offe
Anim																				
Pers	.11																			
Dest	.10	.02																		
Brea	.02	.03	.10																	
Figh	0	.11	0	.02																
Hang	.01	.07	0	.08	.18															
Lyin	0	.06	.01	.29	0	.06														
Phys	.02	.11	.09	.03	.11	.03	.01													
Runs	0	0	.03	0	.08	0	0	.09												
Fire	0	0	0	0	0	0	0	0	.03											
Steh	0	.01	.19	.01	.02	.03	.14	.03	.03	.04										
Steo	.06	0	.04	.02	0	0	.01	.01	.07	.17	.32									
Swea	.03	.07	.12	.05	.01	.01	.06	.03	.01	.02	.02	0								
Thrp	0	.08	.11	.04	.22	.01	0	.25	0	.10	0	.05	.13							
Trua	0	0	.01	.02	0	0	0	0	.10	0	0	0	0	0						
Vand	0	0	.08	0	.11	0	0	.05	.33	0	0	.13	.02	.05	.19					
Lack	0	.06	.10	.25	.05	.05	.08	0	0	0	.04	0	.05	.03	0	.01				
cons	0	.12	.06	.04	0	.02	.05	0	0	0	0	0	0	.01	0	0	.14			
Help	.01	0	0	0	0	0	0	0	0	0	0	0	0	0	0	0	0	.37		
Offe	0	0	0	0	0	0	.01	0	0	0	0	0	0	0	0	0	.02	.26	.37	

Note. It is a symmetric matrix where each entry represents the strength of the connection between two nodes (variables). Global network strength (i.e., the sum of absolute edge values for each network) = 7.81.

Table S31

Adjacency matrix of the estimated network for Sample 2-CU Boys: ABCD-SD baseline, US

	Anim	Pers	Dest	Brea	Figh	Hang	Lyin	Phys	Runs	Fire	Steh	Steo	Swa	Thrp	Trua	Vand	Lack	cons	Help	Offe
Anim																				
Pers	.17																			
Dest	.07	.03																		
Brea	.01	0	.09																	
Figh	0	.14	0	.03																
Hang	0	.03	0	.07	.14															
Lyin	0	.05	0	.28	.01	.07														
Phys	0	.11	.08	.02	.05	.01	.03													
Runs	-.02	.02	0	0	.08	0	0	.03												
Fire	0	0	.01	0	.01	0	0	.03	0											
Steh	0	0	.15	0	.02	.01	.09	.02	0	.09										
Steo	0	0	0	.02	0	0	.02	.01	.09	.23	.30									
Swea	.03	.10	.11	.08	.01	0	.09	.01	.02	.02	.04	0								
Thrp	.01	.10	.14	.04	.17	.06	0	.31	.02	.08	0	.11	.11							
Trua	0	0	.01	.01	0	0	0	0	.17	0	.02	0	0	0						
Vand	0	.02	.09	0	.13	0	0	.05	.46	0	.05	.09	.03	.03	.11					
Lack	0	.09	.13	.25	.04	.03	.07	0	.01	0	.07	0	0	.02	0	.02				
cons	0	.07	.06	.05	0	.06	.04	0	0	0	0	0	0	.02	0	0	.16			
Help	.03	.03	0	.01	0	0	0	0	0	0	0	0	0	.02	0	0	0	.35		
Offe	0	0	0	0	0	0	.02	0	0	0	0	0	0	0	0	0	.02	.26	.38	

Note. It is a symmetric matrix where each entry represents the strength of the connection between two nodes (variables). Global network strength (i.e., the sum of absolute edge values for each network) = 7.98.

Table S32

Adjacency matrix of the estimated network for Sample 2-CU Girls: ABCD-SD baseline, US

	Anim	Pers	Dest	Brea	Figh	Hang	Lyin	Phys	Runs	Fire	Steh	Steo	Swa	Thrp	Trua	Vand	Lack	cons	Help	Offe
Anim																				
Pers	0																			
Dest	.12	0																		
Brea	0	.07	.09																	
Figh	0	.05	.03	.01																
Hang	.09	.12	0	.08	.20															
Lyin	0	.06	.08	.28	0	.05														
Phys	.03	.10	.08	.03	.27	.03	0													
Runs	0	0	.06	0	.03	0	0	.19												
Fire	.11	0	0	0	.01	0	0	-.05	.19											
Steh	0	.02	.21	.07	0	.03	.17	.04	.10	0										
Steo	.15	0	.11	.02	0	0	0	0	.04	.03	.29									
Swea	0	.01	.15	.01	0	0	0	.06	0	0	0	.04								
Thrp	0	.05	.05	.04	.31	0	0	.09	0	.04	.02	0	.14							
Trua	0	0	0	0	.01	.04	0	.04	.01	0	0	0	0	0						
Vand	0	0	.04	0	.07	0	0	.09	.02	0	0	.19	0	0	.24					
Lack	.07	.03	.04	.25	.05	.07	.11	0	0	0	.01	0	.14	.03	0	0				
cons	0	.15	.05	.02	0	0	.05	0	0	0	0	0	0	0	0	0	.14			
Help	0	0	0	0	0	0	0	0	0	0	0	0	0	0	0	0	0	.38		
Offe	0	0	0	0	0	0	0	0	0	0	0	0	0	0	0	0	.01	.26	.35	

Note. It is a symmetric matrix where each entry represents the strength of the connection between two nodes (variables). Global network strength (i.e., the sum of absolute edge values for each network) = 7.76.

Table S33

Adjacency matrix of the estimated network for Sample 3a Full: ELISA wave 6, Spain

	Symp	Shar	Done	Indi	Upse	Remo	Unca	Capa	Guil	Expr	Teas	Viol	Thre	Hitw	Hitp	Take	Brok	Inte
Symp																		
Shar	.26																	
Done	.07	.04																
Indi	.11	.26	.03															
Upse	.07	.09	.09	.15														
Remo	0	0	.14	.05	.07													
Unca	.12	.09	.04	.24	.06	.09												
Capa	.05	.02	.06	.11	.06	.09	.09											
Guil	.01	.08	.18	0	0	.21	.05	.20										
Expr	.06	.02	.06	.10	.04	.09	.04	.33	.13									
Teas	0	0	0	0	.08	.02	.12	0	0	0								
Viol	0	0	.04	.02	0	.08	0	.05	.06	0	.09							
Thre	-.01	0	-.02	0	.05	0	.01	0	0	0	.19	.21						
Hitw	0	.01	0	0	.04	.01	-.01	.06	0	.01	.04	.07	.14					
Hitp	0	0	0	0	0	.01	0	0	0	.03	.14	.08	.11	.22				
Take	0	0	0	0	.03	.07	0	0	0	.02	.10	.17	.03	.02	0			
Brok	0	0	0	-.04	.07	.01	.01	.02	.02	.02	0	.03	.04	.22	.06	.14		
Inte	0	0	0	.01	0	0	.01	0	0	.01	.10	.06	.09	.09	.10	.18	.18	

Note. It is a symmetric matrix where each entry represents the strength of the connection between two nodes (variables). Global network strength (i.e., the sum of absolute edge values for each network) = 8.13.

Table S34

Adjacency matrix of the estimated network for Sample 3a Boys: ELISA wave 6, Spain

	Symp	Shar	Done	Indi	Upse	Remo	Unca	Capa	Guil	Expr	Teas	Viol	Thre	Hitw	Hitp	Take	Brok	Inte
Symp																		
Shar	0																	
Done	.25	.02																
Indi	.09	.11	.18															
Upse	.01	.09	.07	.09														
Remo	.18	.08	.29	.07	.08													
Unca	.06	.07	.16	.06	.05	.07												
Capa	.12	.22	0	0	.20	0	.17											
Guil	.04	.07	.06	.04	.13	.08	.31	.16										
Expr	0	0	0	.08	.04	.10	0	0	0									
Teas	0	.01	.03	0	.11	0	.05	.05	0	.10								
Viol	0	0	0	.06	0	0	0	0	0	.16	.20							
Thre	0	0	0	.04	0	0	.03	0	.05	.06	.04	.20						
Hitw	0	0	0	0	0	0	0	0	.02	.18	.08	.12	.15					
Hitp	0	0	0	0	.07	0	0	0	.01	.08	.17	.05	.04	.02				
Take	0	0	-.04	.08	0	0	.01	0	.02	0	.05	.07	.22	.05	.14			
Brok	0	0	0	.02	0	0	0	.01	0	.06	.09	.06	.13	.10	.17	.14		
Inte	0	0	.25	.09	.01	.18	.06	.12	.04	0	0	0	0	0	0	0	0	

Note. It is a symmetric matrix where each entry represents the strength of the connection between two nodes (variables). Global network strength (i.e., the sum of absolute edge values for each network) = 8.29.

Table S35

Adjacency matrix of the estimated network for Sample 3a Girls: ELISA wave 6, Spain

	Symp	Shar	Done	Indi	Upse	Remo	Unca	Capa	Guil	Expr	Teas	Viol	Thre	Hitw	Hitp	Take	Brok	Inte
Symp																		
Shar	.24																	
Done	.08	.11																
Indi	.08	.31	.02															
Upse	.01	.12	.05	.11														
Remo	0	0	.20	.02	.06													
Unca	.09	.05	.03	.20	.06	.09												
Capa	.07	.01	.04	.05	.08	.12	.12											
Guil	.05	.03	.11	.05	0	.23	.09	.22										
Expr	.02	.04	.06	.15	.05	.04	.02	.35	.09									
Teas	.01	0	0	0	.07	0	.13	0	0	0								
Viol	0	0	.05	0	0	.04	0	.05	.06	0	.09							
Thre	0	0	0	0	.01	0	0	0	0	0	.20	.21						
Hitw	0	.02	0	0	.03	.03	0	.06	0	0	.04	.10	.07					
Hitp	0	.02	0	0	0	0	0	.05	0	0	.06	.06	.10	.28				
Take	0	0	.02	0	.05	.05	0	0	0	.02	.12	.15	0	0	0			
Brok	0	0	0	0	.06	.01	0	.02	.05	0	0	.02	.01	.20	.07	.13		
Inte	0	0	0	.02	0	0	.02	0	0	.02	.13	.03	.12	.04	.10	.17	.22	

Note. It is a symmetric matrix where each entry represents the strength of the connection between two nodes (variables). Global network strength (i.e., the sum of absolute edge values for each network) = 7.71.

Table S36

Adjacency matrix of the estimated network for Sample 3b Full: ELISA wave 7, Spain

	Anim	Pers	Dest	Brea	Figh	Hang	Lyin	Phys	Runs	Fire	Steh	Steo	Swea	Thrp	Trua	Vand	Lack	Cons	Help	Offe
Anim																				
Pers	.08																			
Dest	.05	.06																		
Brea	0	0	.08																	
Figh	0	.12	.04	.10																
Hang	0	0	0	.03	.10															
Lyin	0	.05	0	.27	0	0														
Phys	0	.06	.06	.04	.05	.02	.01													
Runs	.02	0	0	0	0	0	0	0												
Fire	0	0	.05	0	.15	.01	0	.01	0											
Steh	0	0	0	0	0	0	0	.06	.01	0										
Steo	.01	0	0	0	0	0	0	.04	.01	.10	.13									
Swea	0	.02	.09	.10	.02	.02	.06	.03	0	.05	.01	0								
Thrp	0	.05	0	.01	.03	0	0	.20	0	.02	0	.05	.11							
Trua	0	0	.04	0	0	.01	0	0	0	0	0	0	0	0						
Vand	.05	0	0	0	.02	.04	0	.10	0	.01	0	.02	0	.10	0					
Lack	.03	.17	.05	.09	0	0	.06	0	.10	.05	0	0	0	0	0	0				
Cons	0	.11	0	.04	0	0	.07	.10	0	0	0	0	0	0	0	0	.13			
Help	0	0	0	0	0	0	.04	0	0	0	0	0	0	0	0	0	0	.31		
Offe	0	0	0	.05	0	0	.01	0	0	0	0	0	.03	0	0	0	0	.11	.22	

Note. It is a symmetric matrix where each entry represents the strength of the connection between two nodes (variables). Global network strength (i.e., the sum of absolute edge values for each network) = 4.77.

Table S37

Adjacency matrix of the estimated network for Sample 3b Boys: ELISA wave 7, Spain

	Anim	Pers	Dest	Brea	Figh	Hang	Lyin	Phys	Runs	Fire	Steh	Steo	Swea	Thrp	Trua	Vand	Lack	Cons	Help	Offe
Anim																				
Pers	.12																			
Dest	0	.05																		
Brea	0	0	.05																	
Figh	0	.19	.03	.11																
Hang	0	0	0	.02	.12															
Lyin	0	.02	0	.26	0	0														
Phys	0	.01	.09	.05	.06	0	0													
Runs	.09	0	0	0	0	0	0	0												
Fire	0	0	0	0	.12	.06	0	0	0											
Steh	0	0	0	0	.06	0	.02	.05	.05	0										
Steo	0	0	0	0	0	.01	0	.06	0	.04	.13									
Swea	0	.02	.10	.13	.03	.03	.09	.01	0	.01	0	.01								
Thrp	0	.16	0	.02	.05	0	0	.14	0	0	0	.03	.10							
Trua	0	0	0	0	0	0	0	0	0	0	0	0	0	0						
Vand	.01	0	0	0	.03	.02	0	.15	0	0	0	0	.03	0	0					
Lack	.01	.18	.09	.09	0	0	.11	.03	.07	.01	0	0	0	.03	0	0				
Cons	0	.11	0	.03	0	0	.04	.07	0	0	0	0	0	0	0	0	.11			
Help	0	0	0	0	0	0	.04	0	0	0	0	0	0	0	0	0	0	.26		
Offe	0	0	0	.06	0	0	0	0	0	0	0	0	.07	0	0	0	0	.15	.24	

Note. It is a symmetric matrix where each entry represents the strength of the connection between two nodes (variables). Global network strength (i.e., the sum of absolute edge values for each network) = 4.72.

Table S38

Adjacency matrix of the estimated network for Sample 3b Girls: ELISA wave 7, Spain

	Anim	Pers	Dest	Brea	Figh	Hang	Lyin	Phys	Runs	Fire	Steh	Steo	Swea	Thrp	Trua	Vand	Lack	Cons	Help	Offe
Anim																				
Pers	.12																			
Dest	0	.01																		
Brea	0	0	0																	
Figh	0	.30	0	.10																
Hang	0	0	0	0	.13															
Lyin	0	0	0	.22	0	0														
Phys	0	0	.11	.04	.08	0	0													
Runs	.21	.11	0	0	0	0	.02	0												
Fire	0	.04	0	0	.04	.12	0	0	0											
Steh	0	0	.01	0	.11	.03	.02	.02	.08	0										
Steo	0	0	.03	0	.04	0	0	.05	0	0	.13									
Swea	0	0	.10	.16	.04	0	.11	0	0	0	0	0								
Thrp	0	.25	.03	.02	.04	0	0	.08	0	0	.07	.01	.10							
Trua	0	0	0	0	0	0	0	0	0	.17	0	0	0	0						
Vand	0	0	0	0	.01	0	0	.18	0	0	0	0	.04	0	0					
Lack	0	.15	.13	.08	0	0	.17	.07	0	0	0	0	.01	.12	0	0				
Cons	0	.09	0	0	0	0	0	.01	0	0	0	0	0	0	0	0	.11			
Help	0	0	0	0	0	0	.02	0	0	0	0	0	0	0	0	0	0	.17		
Offe	0	0	0	.04	0	0	0	0	0	0	0	0	.09	0	0	0	0	.17	.25	

Note. It is a symmetric matrix where each entry represents the strength of the connection between two nodes (variables). Global network strength (i.e., the sum of absolute edge values for each network) = 5.14.

6. Network Comparisons Test (Pearson Correlations)

Table S39

Results of Network Comparisons Tests (Restricted to Networks Estimated with the Same Measures)

a) Between Boys and Girls Within Each Sample

Sample	Networks Comparisons		Network structure invariance		Global strength invariance	
			M	***p*-value**	***S***	***p*-value**
Sample 1	Boys	Girls	0.10	.921	0.31	.277
Sample 2-ICU	Boys	Girls	0.45	.030	0.36	.452
Sample 2-CU	Boys	Girls	0.46	.030	0.50	.307
Sample 3a	Boys	Girls	0.18	.515	0.25	.287
Sample 3b	Boys	Girls	0.18	.515	0.25	.287

b) Between Countries Within Each Gender Group

Gender	Networks Comparisons		Network structure invariance		Global strength invariance	
			M	***p*-value**	***S***	***p*-value**
Boys	Sample 1	Sample 2-CU	0.42	.010	0.69	.119
	Sample 1	Sample 3b	0.39	.273	3.24	.091
	Sample 2-CU	Sample 3b	0.46	.091	3.93	.091
Girls	Sample 1	Sample 2-CU	0.24	.454	0.51	.454
	Sample 1	Sample 3b	0.35	.501	3.13	.501
	Sample 2-CU	Sample 3b	0.34	.545	3.64	.091

Note. As an additional analysis, we applied the Network Comparison Test (NCT; van Borkulo et al., 2019) to investigate differences between boys and girls within each sample, as well as between boys across samples and girls across samples. These comparisons were based on networks estimated from Pearson correlation matrices, since the NCT has been validated primarily for cross-sectional continuous data and, to a lesser extent, for binary data (van Borkulo et al., 2023). Prior to this, we confirmed that the Spearman correlation matrices were highly correlated with the Pearson correlation matrices in all cases ($r > .90$). The NCT employs a permutation-based procedure to assess two key aspects: network structure invariance (whether the overall configuration of edges is equivalent) and global strength invariance (whether the total connectivity, i.e., sum of absolute edge weights, is similar across networks). For methodological rigor, we did not conduct the NCT on our networks based on Spearman correlations. The results from the Pearson-based sensitivity analyses are presented below and should be interpreted with these methodological considerations in mind.

7. Network Analyses of Samples 2-ICU, 2-CU, and 3a

a. Sample 2-ICU: ABCD-SD baseline, US **b. Sample 2-CU: ABCD-SD baseline, US**

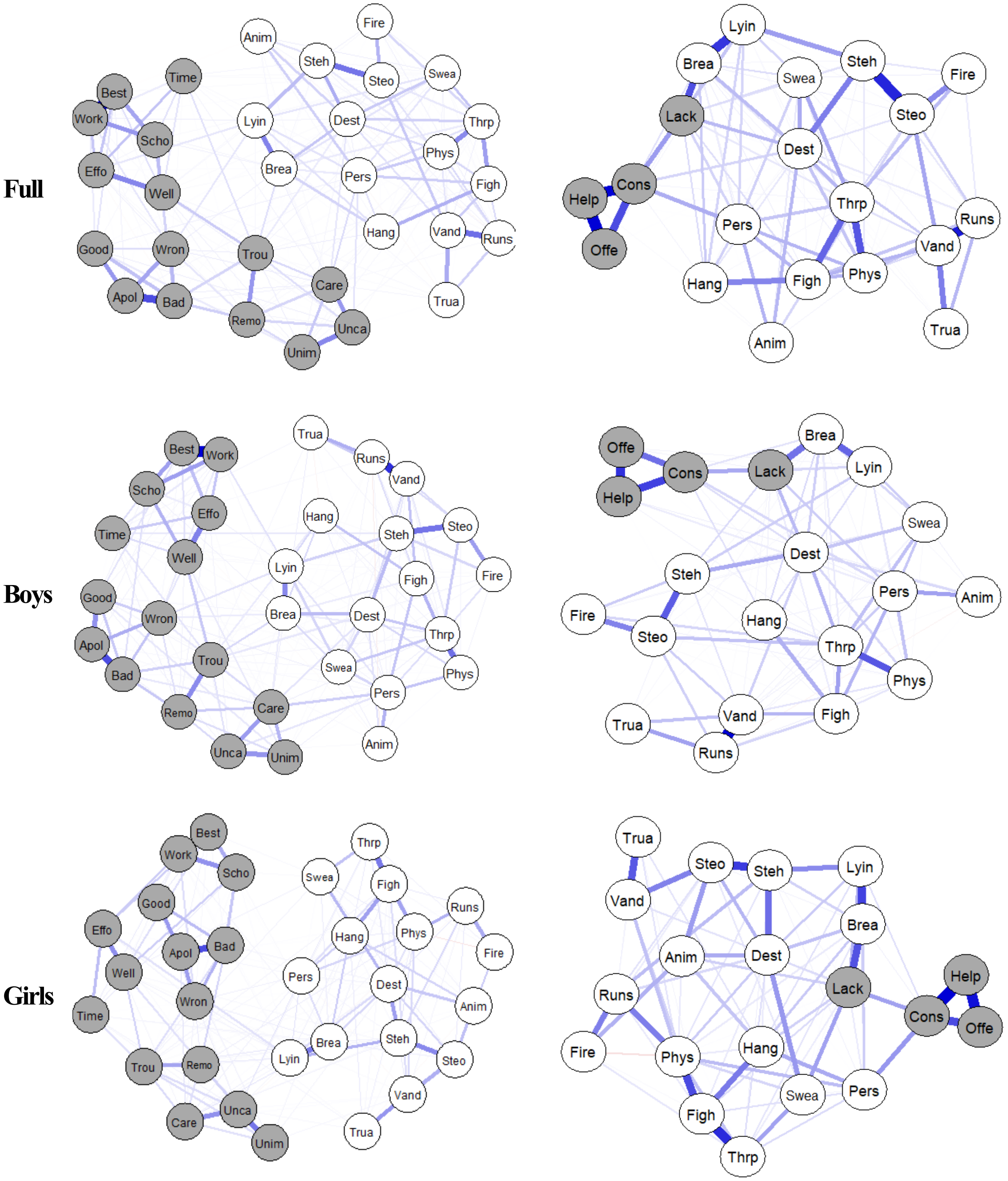

Figure. S1 Network structure for (a) Sample 2-ICU: ABCD-SD baseline, US, (b) Sample 2-CU: ABCD-SD baseline, US, and (c) Sample 3a: ELISA wave 6, Spain. CP items are in white and CU traits in gray.

c. Sample 3a: ELISA wave 6, Spain

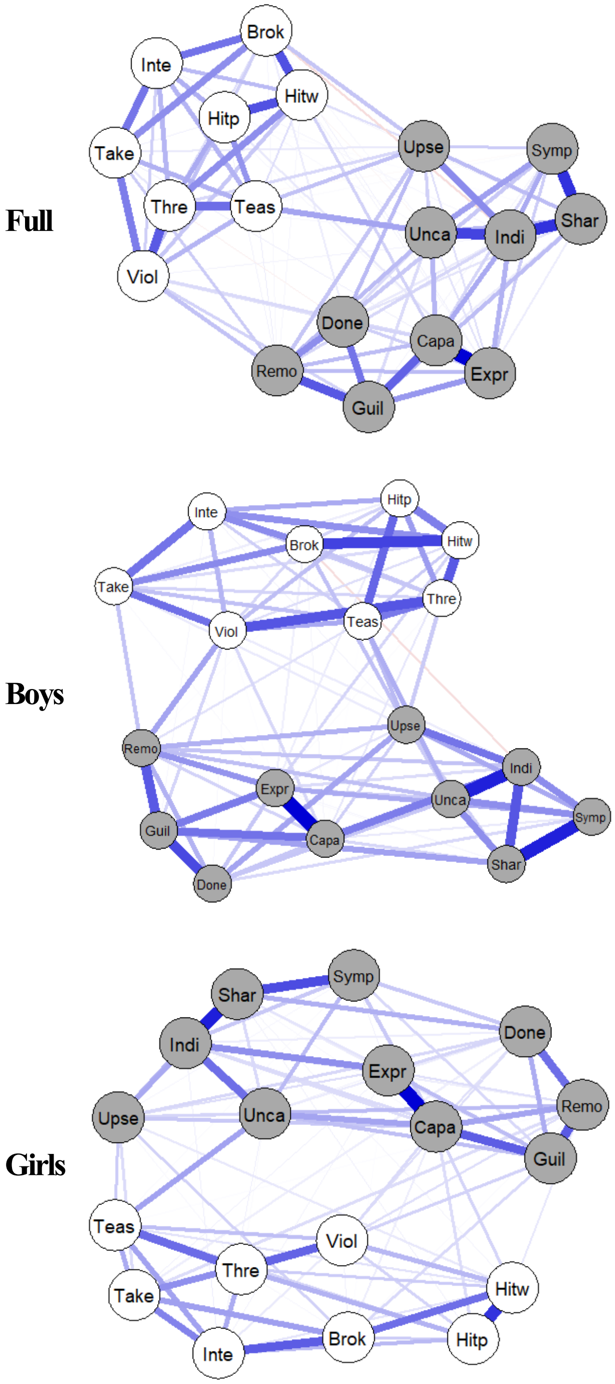

Figure. S1 (Continued).

a. Sample 2-ICU: ABCD-SD baseline, US **b. Sample 2-CU: ABCD-SD baseline, US**

Full

Boys

Girls

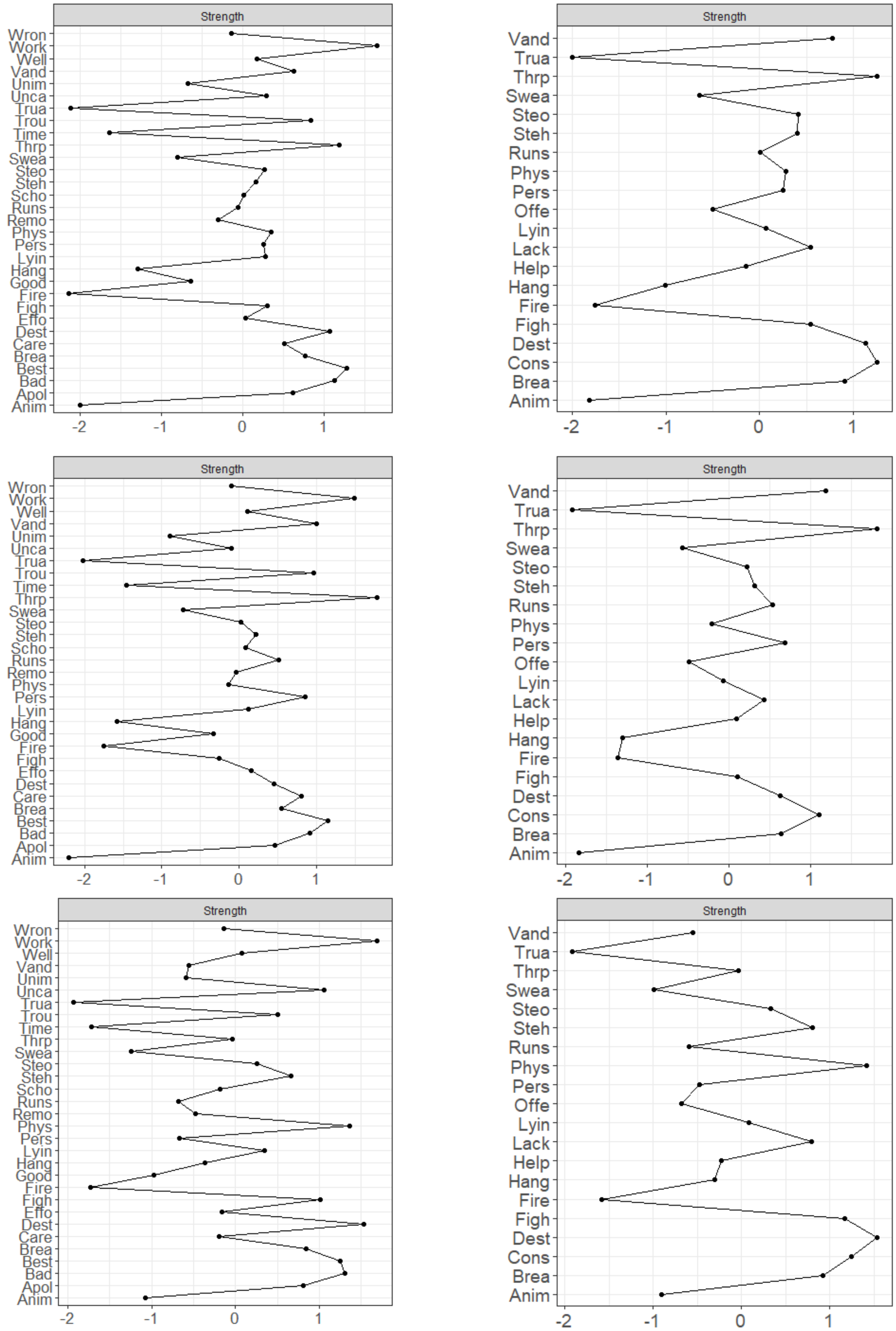

Figure. S2 Centrality strength (*Z*-scores) for (a) Sample 2-ICU: ABCD-SD baseline, US, (b) Sample 2-CU: ABCD-SD baseline, US, and (c) Sample 3a: ELISA wave 6, Spain, presented for the full sample, as well as separately for boys and girls. Strength centrality is the sum of correlations between each node and all other nodes. Items are in descending alphabetical order.

c. Sample 3a: ELISA wave 6, Spain

Full

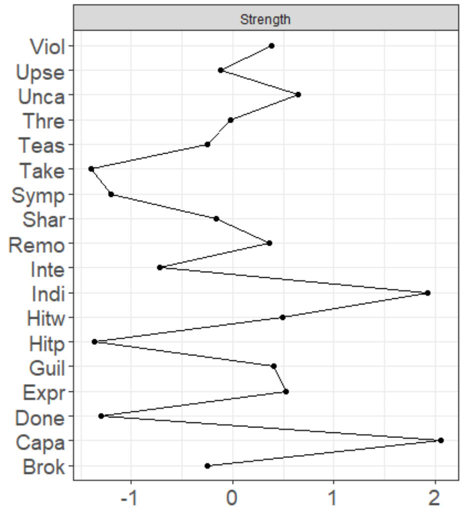

Boys

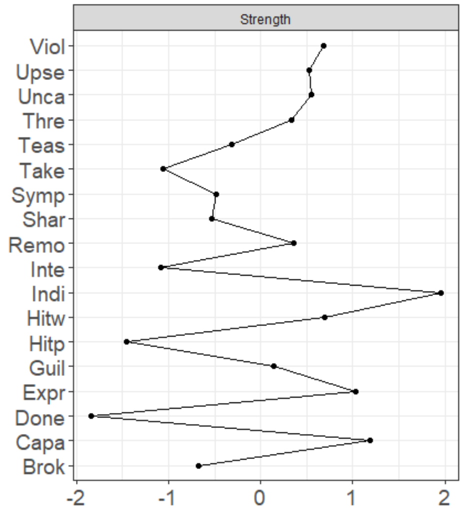

Girls

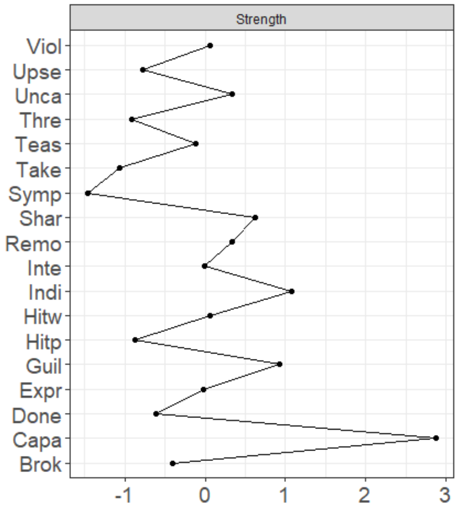

Figure. S2 (Continued)

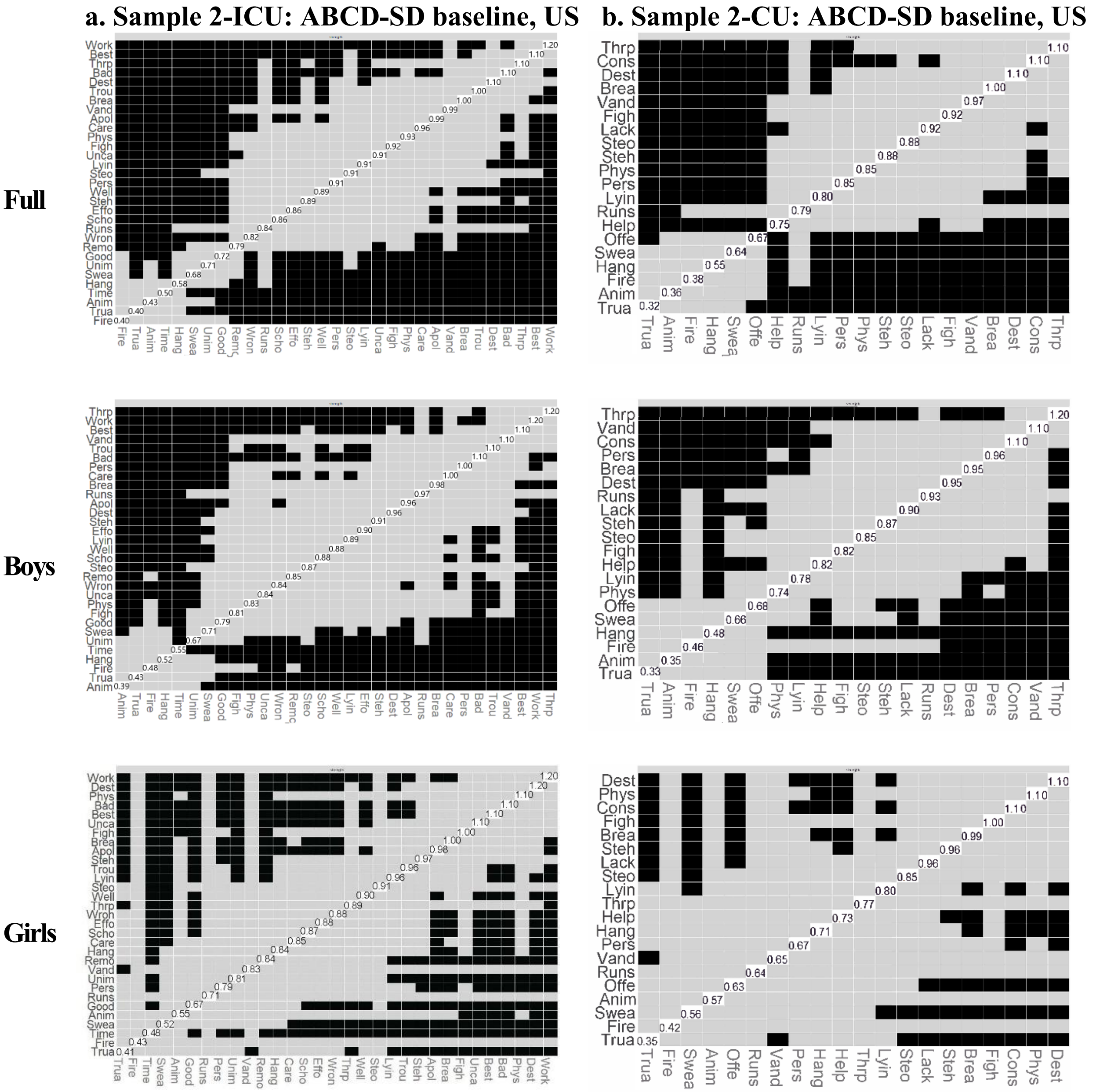

Figure. S3 Bootstrapped differences of centrality strength for (a) Sample 2-ICU: ABCD-SD baseline, US (b) Sample 2-CU: ABCD-SD baseline, US, and (c) Sample 3a: ELISA wave 6, Spain, shown for the full sample, as well as separately for boys and girls. Black boxes denote significant differences (*p*<.05), gray boxes indicate nonsignificant ones, and white diagonal boxes display the corresponding values.

c. Sample 3a: ELISA wave 6, Spain

Full

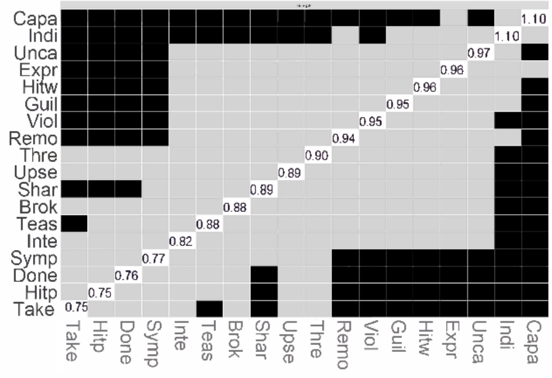

Boys

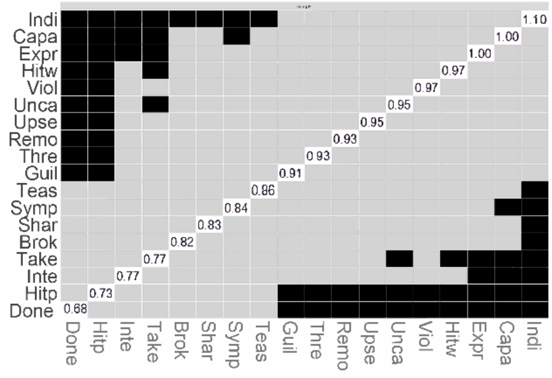

Girls

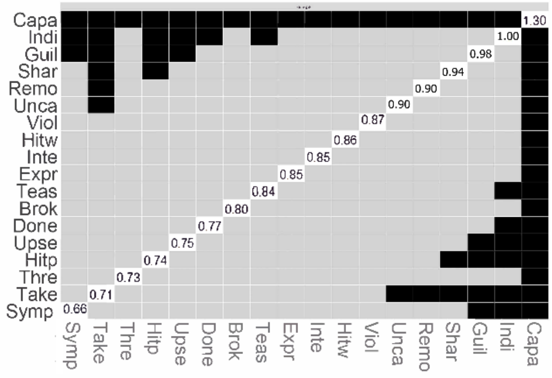

Figure. S3 (Continued)

a. Sample 2-ICU: ABCD-SD baseline, US **b. Sample 2-CU: ABCD-SD baseline. US**

Full

Boys

Girls

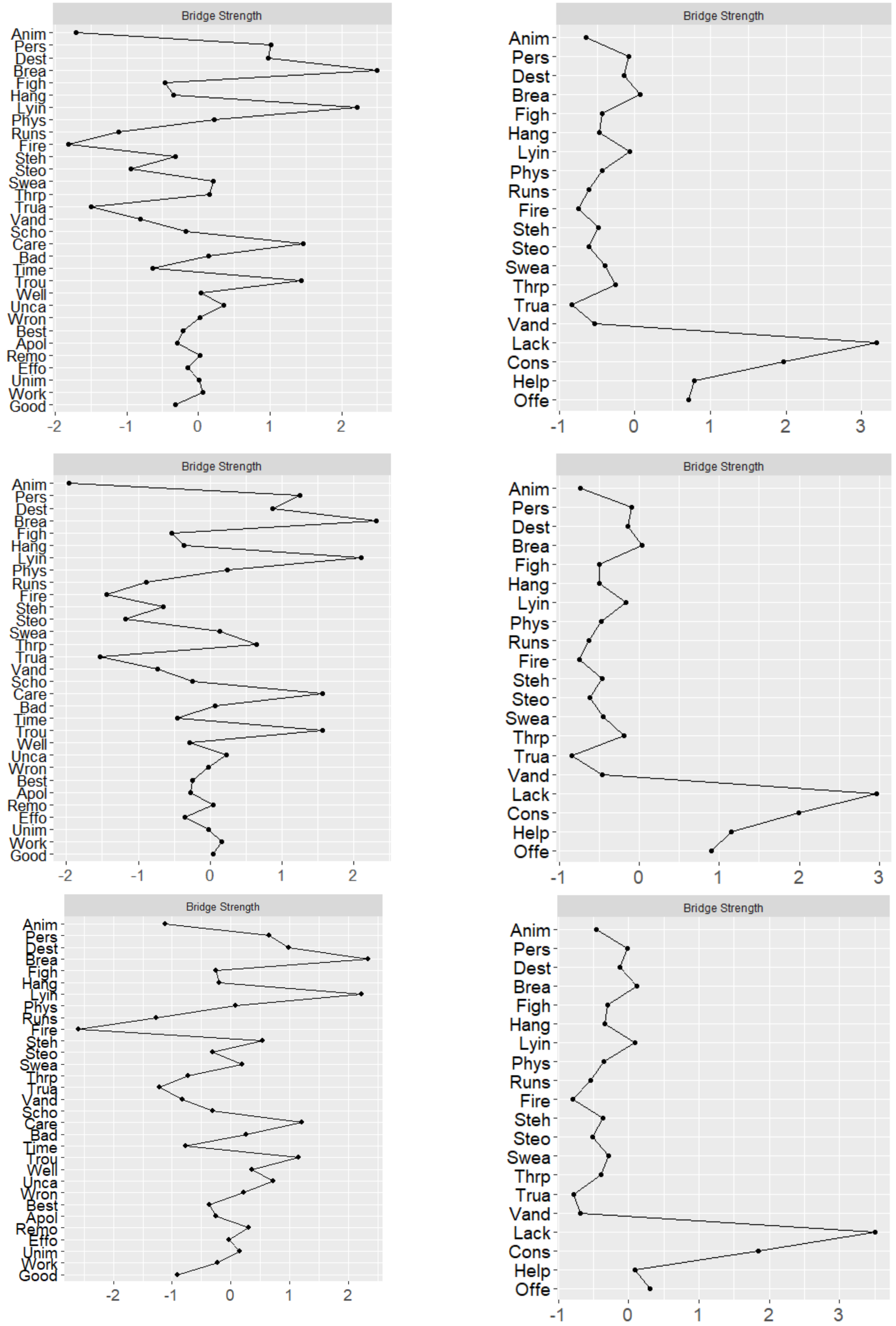

Figure. S4 Bridge strength for (a) Sample 2-ICU: ABCD-SD baseline, US, (b) Sample 2-CU: ABCD-SD baseline, US, and (c) Sample 3a: ELISA wave 6, Spain**,** shown for the full sample, as well as separately for boys and girls. The items are listed alphabetically, with those categorized as CP presented first, followed by those classified as CU traits.

Sample 3a: ELISA wave 6, Spain

Full

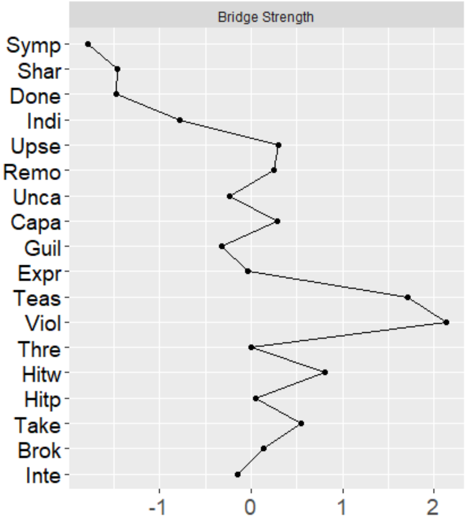

Boys

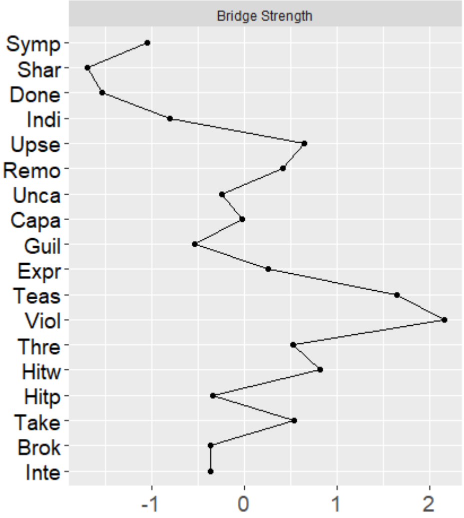

Girls

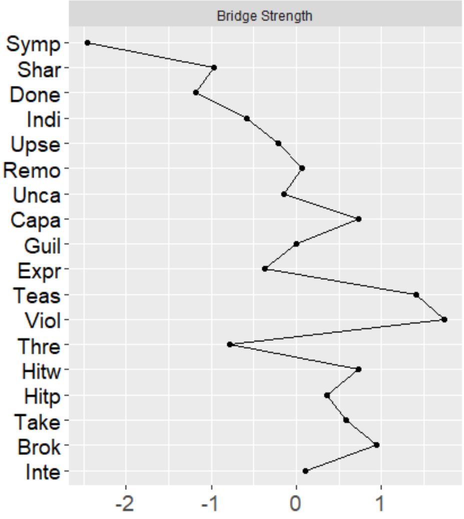

Figure. S4 (Continued)

8. Network Stability Strength across all Samples

Sample 1: ABCD baseline, US

CS = .75

Full

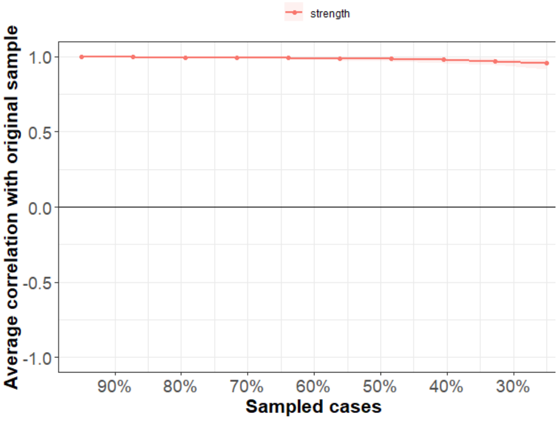

CS = .75

Boys

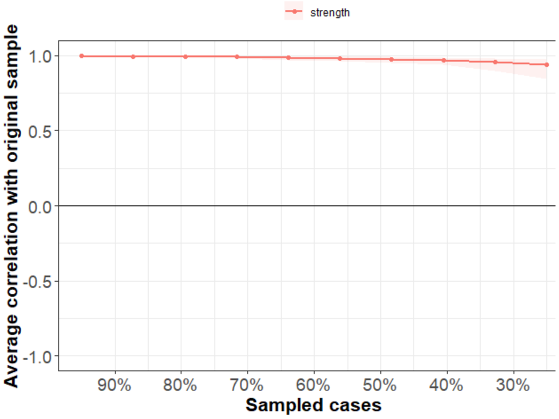

CS = .75

Girls

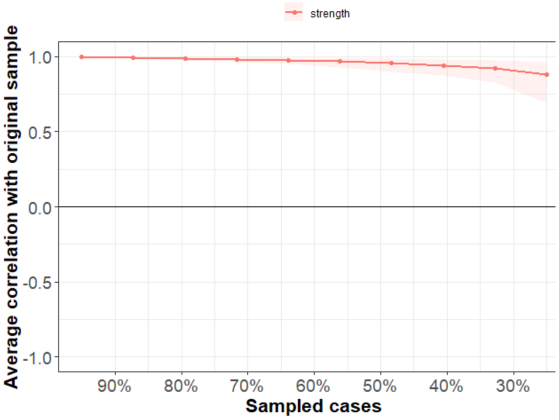

Figure. S5 Stability strength for Sample 1: ABCD baseline, US presented for the full sample, as well as separately for boys and girls. CS = Coefficient of Stability.

Sample 2-ICU: ABCD-SD baseline, US

CS = .75

Full

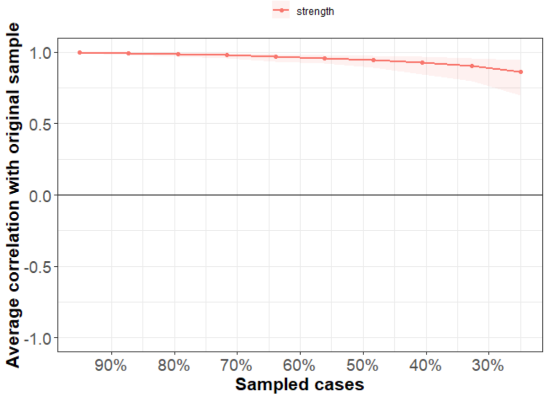

CS = .67

Boys

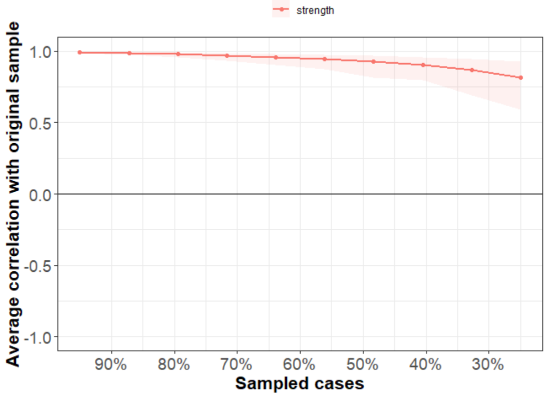

CS = .44

Girls

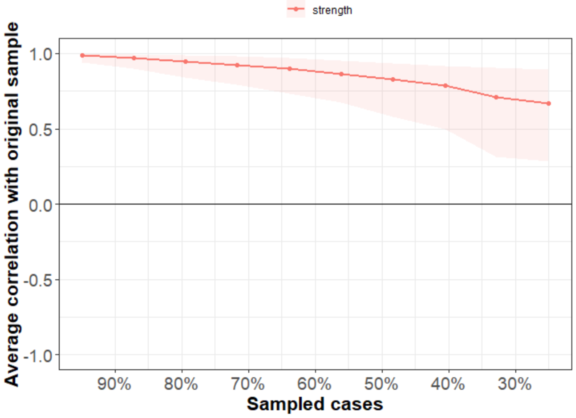

Figure. S6 Stability strength for Sample 2-ICU: ABCD-SD baseline, US presented for the full sample, as well as separately for boys and girls. CS = Coefficient of Stability.

Sample 2-CU: ABCD-SD baseline, US

CS = .67

Full

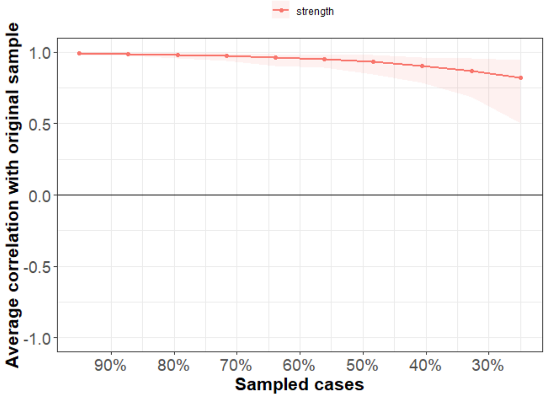

CS = .60

Boys

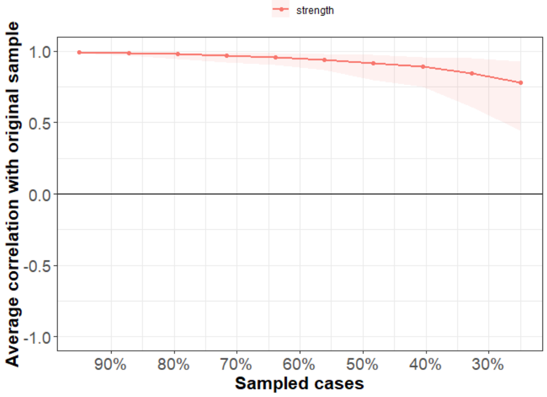

CS = .28

Girls

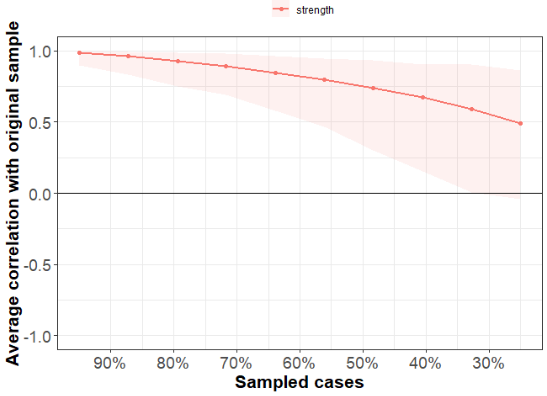

Figure. S7 Stability strength for Sample 2-CU: ABCD-SD baseline, US presented for the full sample, as well as separately for boys and girls. CS = Coefficient of Stability.

Sample 3a: ELISA wave 6, Spain

CS = .67

Full

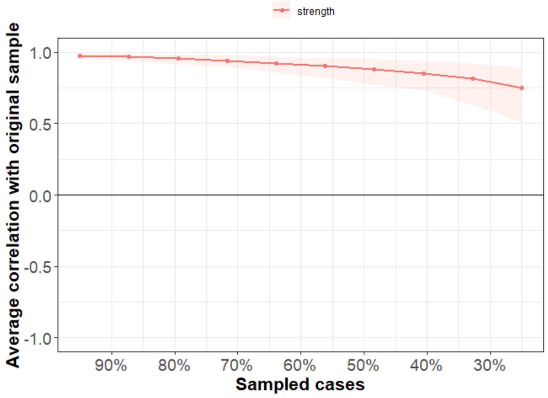

CS = .44

Boys

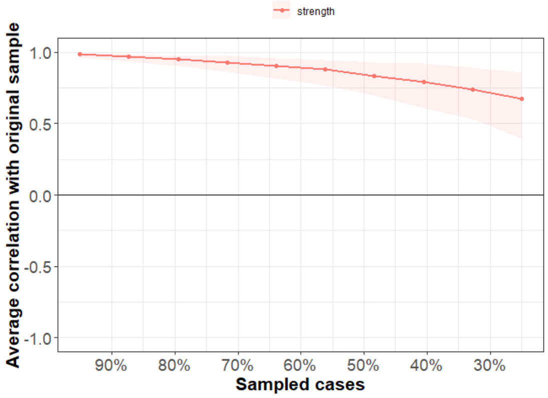

CS = .52

Girls

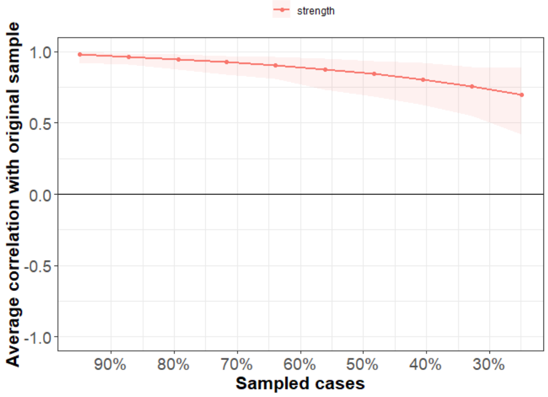

Figure. S8 Stability strength for Sample 3a: ELISA wave 6, Spain presented for the full sample, as well as separately for boys and girls. CS = Coefficient of Stability.

Sample 3b: ELISA wave 7, Spain

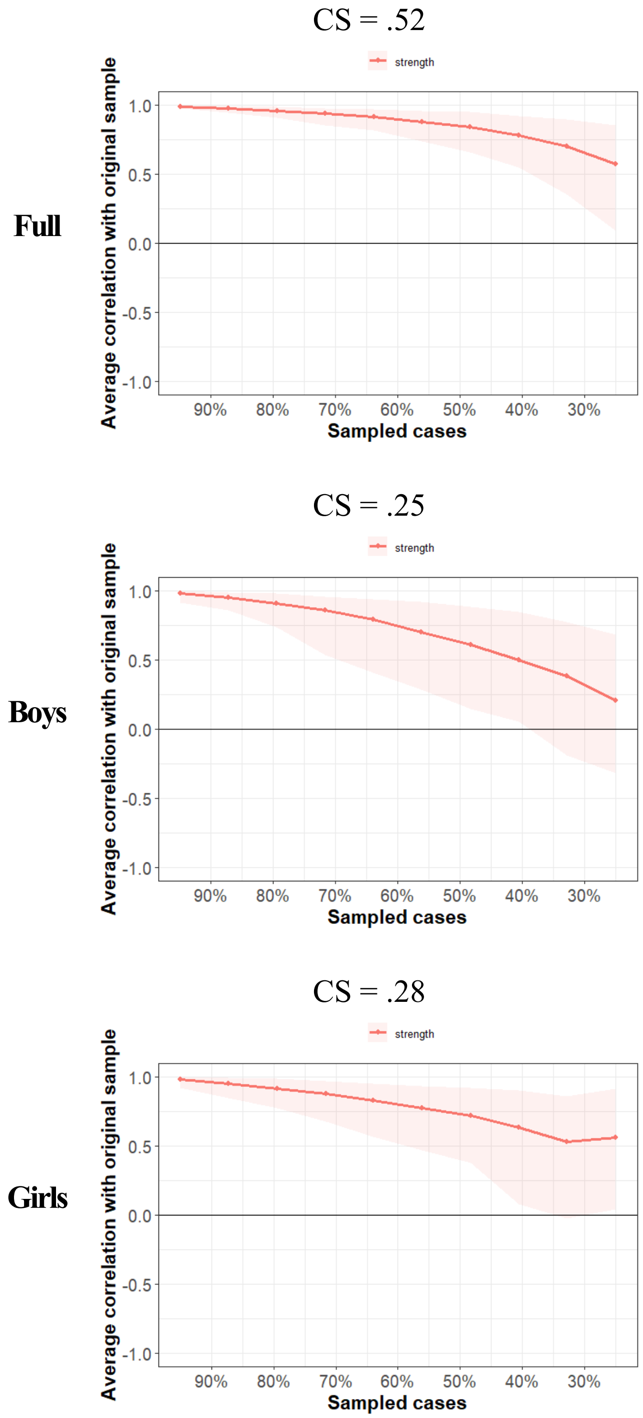

Figure. S9 Stability strength for Sample 3b: ELISA wave 7, Spain presented for the full sample, as well as separately for boys and girls. CS = Coefficient of Stability.

References

Achenbach, T. & Rescorla, L. (2001). *The Manual for the ASEBA School-Age Forms & Profiles.* University of Vermont, Research Center for Children, Youth, and Families.

Albores-Gallo, L., Lara-Muñoz, C., Esperón-Varas, C., Cárdenas Zetina, J. A., Pérez Soriano, A. M., & Villanueva Colin, G. (2007). Validez y fiabilidad del CBCL/6-18. Incluye las escalas del DSM [Validity and reliability of the CBCL/6-18. Includes the DSM scales]. *Actas Españolas de Psquiatría*, *35*(6), 393–399.

Álvarez de la Granja, M., Parafita Couto, M. C., Sá-Leite, A. R., Fraga, I., Duñabeitia, J. A., Pliatsikas, C., & Comesaña, M. (2025). The Galician MultiPic: a picture dataset that captures lexical variation. *Frontiers in Psychology*, *16*, 1551000. https://doi.org/10.3389/fpsyg.2025.1551000

Álvarez-Voces, M., & Romero, E. (2025). Gender differences in the development of children's conduct problems: A four-year longitudinal study. *Journal of Child and Family Studies, 34*(2), 518–531. https://doi.org/10.1007/s10826-024-02999-5

Barrau, V., López-Romero, L., Bosch, R., Torrubia, R., Casas, M., & Molinuevo, B. (2022). Further validation of the Spanish parent-reported Child Problematic Traits Inventory: discriminant validity for distinguishing children vulnerable to externalizing and other psychopathology conditions. *Journal of Psychopathology and Behavioral Assessment*, 44(1), 245–260. https://doi.org/10.1007/s10862-021-09921-z

Colins, O. F., Andershed, H., Frogner, L., Lopez-Romero, L., Veen, V., & Andershed, A. K. (2014). A new measure to assess psychopathic personality in children: The Child Problematic Traits Inventory. *Journal of Psychopathology and Behavioral*

Assessment, *36*(1), 4–21. https://doi.org/10.1007/s10862-013-9385-y

Fanti, K. A., Mavrommatis, I., Colins, O., & Andershed, H. (2023). Fearlessness as an Underlying Mechanism Leading to Conduct Problems: Testing the Intermediate Effects of Parenting, Anxiety, and callous-unemotional Traits. Research on Child and Adolescent Psychopathology, 51(8), 1115–1128. https://doi.org/10.1007/s10802-023-01076-7

Figueiredo, P., Moreira, D., Ramião, E., Barroso, R., & Barbosa, F. (2022). Psychometric properties of the Portuguese teacher-version of the Inventory of Callous-Unemotional Traits. *Clinical Child Psychology and Psychiatry*, *27*(3), 852–869. https://doi.org/10.1177/13591045211070168

Frick, P. J. (2004). Inventory of callous–unemotional traits. *PLoS One*.

Gao, Y., & Zhang, W. (2016). Confirmatory factor analyses of self- and parent-report inventory of Callous-Unemotional Traits in 8- to 10-year-olds. Journal of Psychopathology and Behavioral Assessment, 38(3), 331–340. https://doi.org/10.1007/s10862-015-9527-5

García, P., Mazaira, J. A. ., & Goodman, R. (2000). Validación inicial de la versión gallega del Cuestionario de Capacidades y Dificultades (SDQ) [Initial validation of the Galician version of the Strength and Difficulties Questionnaire.]. *Revista De Psiquiatría Infanto-Juvenil*, (2), 95–100. https://aepnya.eu/index.php/revistaaepnya/article/view/467

Goodman, R. (1997). The Strengths and Difficulties Questionnaire: a research note. *Journal of Child Psychology and Psychiatry, and Allied Disciplines*, *38*(5), 581–586. https://doi.org/10.1111/j.1469-7610.1997.tb01545.x

He, J. P., Burstein, M., Schmitz, A., & Merikangas, K. R. (2013). The Strengths and Difficulties Questionnaire (SDQ): the factor structure and scale validation in U.S. adolescents.

Journal of Abnormal Child Psychology, 41(4), 583–595. https://doi.org/10.1007/s10802-012-9696-6

Kimonis, E. R., Branch, J., Hagman, B., Graham, N., & Miller, C. (2013). The psychometric properties of the inventory of callous-unemotional traits in an undergraduate sample. *Psychological Assessment*, *25*(1), 84–93. https://doi.org/10.1037/a0029024

Lacalle Sisteré, M., Domènech Massons, J. M., Granero Pérez, R., & Ezpeleta Ascaso, L. (2014). Validity of the DSM-Oriented scales of the Child Behavior Checklist and Youth Self-Report. Psicothema, 26(3), 364–371. https://doi.org/10.7334/psicothema2013.342

Little, R. J. (1988). A test of missing completely at random for multivariate data with missing values. *Journal of the American Statistical Association, 83*, 1198–1202. https://doi.org/10.2307/2290157

López-Romero, L., Molinuevo, B., Bonillo, A., Andershed, H., Colins, O. F., Torrubia, R., & Romero, E. (2019). Psychometric properties of the Spanish version of the Child Problematic Traits Inventory in 3-to 12-year-old Spanish children. European Journal of Psychological Assessment, 35(6), 842–854. https://doi.org/10.1027/1015-5759/a000458

McDonald, S. E., Ma, L., Green, K. E., Hitti, S. A., Cody, A. M., Donovan, C., Williams, J. H., & Ascione, F. R. (2018). Evaluation of the parent-report Inventory of Callous-Unemotional Traits in a sample of children recruited from intimate partner violence services: A Multidimensional Rasch Analysis. Journal of Clinical Psychology, 74(3), 418–441. https://doi.org/10.1002/jclp.22497

Ortuño-Sierra, J., Aritio-Solana, R., & Fonseca-Pedrero, E. (2018). Mental health difficulties in children and adolescents: The study of the SDQ in the Spanish National Health Survey

2011-2012. Psychiatry Research, 259, 236–242. https://doi.org/10.1016/j.psychres.2017.10.025

Rodríguez-Hernández, P. J., Betancort, M., Ramírez-Santana, G. M., García, R., Sanz-Álvarez, E. J., & De las Cuevas-Castresana, C. (2012). Psychometric properties of the parent and teacher versions of the Strength and Difficulties Questionnaire (SDQ) in a Spanish sample. International Journal of Clinical and Health Psychology, 12(2), 265–279.

Ullman, J. B., Tabachnick, B. G., & Fidell, L. S. (2001). Structural equation modeling. In B. G. Tabachnick & L. S. Fidell (Eds.), *Using Multivariate Statistics* (4th ed., pp. 653–771). Pearson Education.

Venta, A., Cuervo, M., Bautista, A., & Walker, J. (2022). A strong contender for mental health screening in Latinx immigrant youth: Psychometric properties of the Strengths and Difficulties Questionnaire. Psychiatry Research, 316, 114780. https://doi.org/10.1016/j.psychres.2022.114780

Van Borkulo, C. D., Epskamp, S., Jones, P., Haslbeck, J., & Millner, A. (2019). *NetworkComparisonTest: Statistical comparison of two networks based on threeinvariance measures* (2.2.1) [Computer software]. https://CRAN.Rproject.org/package=NetworkComparisonTest

van Borkulo, C. D., van Bork, R., Boschloo, L., Kossakowski, J. J., Tio, P., Schoevers, R. A., Borsboom, D., & Waldorp, L. J. (2023). Comparing network structures on three aspects: A permutation test. *Psychological Methods*, *28*(6), 1273–1285. https://doi.org/10.1037/met0000476

Yoshida, S., Adachi, M., Takahashi, M., Takanyanagi, N., Yasuda, S., Osada, H., & Nakamura, K. (2019). The factor structure and construct validity of the parent-reported Inventory of Callous-Unemotional Traits among school-aged children and adolescents. *PloS one*, *14*(8), e0221046. https://doi.org/10.1371/journal.pone.0221046
